# Development and validation of a lung biological equivalent dose-based multiregional radiomic model for predicting symptomatic radiation pneumonitis after SBRT in lung cancer patients

**DOI:** 10.3389/fonc.2024.1489217

**Published:** 2024-12-06

**Authors:** Yuxin Jiao, Aihui Feng, Shihong Li, Yanping Ren, Hongbo Gao, Di Chen, Li Sun, Xiangpeng Zheng, Guangwu Lin

**Affiliations:** ^1^ Department of Radiation Oncology, Huadong Hospital, Fudan University, Shanghai, China; ^2^ Department of Radiation Oncology, Shanghai Chest Hospital, Shanghai Jiao Tong University School of Medicine, Shanghai, China; ^3^ Department of Radiology, Huadong Hospital, Fudan University, Shanghai, China

**Keywords:** SBRT, symptomatic radiation pneumonitis, radiomics, machine learning, lung cancer

## Abstract

**Background:**

This study aimed to develop and validate a multiregional radiomic-based composite model to predict symptomatic radiation pneumonitis (SRP) in non-small cell lung cancer (NSCLC) patients treated with stereotactic body radiation therapy (SBRT).

**Materials and methods:**

189 patients from two institutions were allocated into training, internal validation and external testing cohorts. The associations between the SRP and clinic-dosimetric factors were analyzed using univariate and multivariate regression. Radiomics features were extracted from seven discrete and three composite regions of interest (ROIs), including anatomical, physical dosimetry, and biologically equivalent dose (BED) dimensions. Correlation filters and Lasso regularization were applied for feature selection and five machine learning algorithms were utilized to construct radiomic models. Multiregional radiomic models integrating features from various regions were developed and undergone performance test in comparison with single-region models. Ultimately, three models—a radiomic model, a dosimetric model, and a combined model—were developed and evaluated using receiver operating characteristic (ROC) curve, model calibration, and decision curve analysis.

**Results:**

V_BED70_ (α/β = 3) of the nontarget lung volume was identified as an independent dosimetric risk factor. The multiregional radiomic models eclipsed their single-regional counterparts, notably with the incorporation of BED-based dimensions, achieving an area under the curve (AUC) of 0.816 [95% CI: 0.694–0.938]. The best predictive model for SRP was the combined model, which integrated the multiregional radiomic features with dosimetric parameters [AUC=0.828, 95% CI: 0.701–0.956]. The calibration and decision curves indicated good predictive accuracy and clinical benefit, respectively.

**Conclusions:**

The combined model improves SRP prediction across various SBRT fractionation schemes, which warrants further validation and optimization using larger-scale retrospective data and in prospective trials.

## 1 Introduction

Lung cancer remains the most common malignant tumor worldwide. Nearly 20% of older patients with early-stage non-small cell lung cancer (NSCLC) may not be able to tolerate surgery due to concomitant comorbidities. Hypofractionated stereotactic body radiation therapy (SBRT) has been proven to be more effective than conventional radiotherapy (1–3), significantly improving local control and overall survival (2, 4). Despite of the favorable safety profile of SBRT, symptomatic radiation pneumonitis (SRP) is the most common adverse reaction, with a reported incidence ranging from 9% to 28% (5), and may lead to treatment failure or even death due to progressive fibrosing interstitial reaction and pulmonary dysfunction (6, 7). Therefore, accurate prediction of SRP and associated factors is important for treatment plan optimization and prophylactic management to minimize the incidence and severity of SRP.

Many dosimetric parameters, including the gross target volume (GTV), planning target volume (PTV), and internal target volume (ITV), the mean lung dose (MLD), Vx ranging from 2.5 to 50, and maximal dose, are reportedly associated with the probability of SRP (5, 8, 9). Due to individual variations in radiosensitivity, the risk of developing SRP varies even using the same treatment protocol. However, these aforementioned dosimetric indicators only reflect the characteristics of the treatment protocol itself and lack information related to the heterogeneity of radiosensitivity of normal lung tissue.

Radiomics is a high-throughput analytical technique that can extract many quantitative features from medical images, and increasing attention has been given to radiotherapy efficacy and adverse reaction prediction (10, 11). In a recent study on conventional intensity-modulated radiotherapy (IMRT), researchers reported that the combination of dose distribution and radiomic features within different anatomical regions of interest (ROIs) of nontarget lungs improved the accuracy of SRP prediction in lung cancer patients (9). In another study, an incremental-dose-interval-based lung subregion segmentation (IDLSS) method, which defined ROIs corresponding to different dose coverage areas, was shown to be of value of predicting SRP (12). These studies have proven that radiomic features from multiregional ROIs in radiotherapy images can provide additional information beyond the single regional ROI, as well as dosimetric and traditional clinical parameters, and thus may better reflect the normal lung tissue radiosensitivity of the individual patient and the risk of developing SRP.

Our previous study revealed that the biologically effective dose (BED) of normal lung tissue serves as an independent risk factor for SRP, with high consistency observed between the coverage area of BED_70Gy_ (α/β = 3) and the range of radiation pneumonitis (13). However, the majority of published radiomics studies regarding predicting SBRT-related SRP established their conclusions on traditional anatomical dimensions and standard fractionated radiation regimes, considering little the fractionation heterogeneity and the ensuing impact on the complications of the lung tissues in terms of BED (14–17). To the best of our knowledge, there are currently no radiomic studies that integrate multidimensional ROIs, particularly those incorporating the BED dimension of the nontarget lung, to predict the occurrence of SRP following SBRT.

This study introduced a sophisticated multiregional radiomic model that incorporated imaging and dose distribution data corrected for fractionation in radiotherapy planning across three dimensions (anatomy, traditional physical dose, and biologically equivalent dose) to predict SRP in patients with NSCLC treated with SBRT. Furthermore, we developed dosimetric, radiomic, and combined models using presumptive dosimetric parameters and the best radiomic features, thus revealing the great significance of the individualization of SBRT radiotherapy planning and the early diagnosis and treatment of SRP.

## 2 Materials and methods

### 2.1 Patients

**Figure 1:**
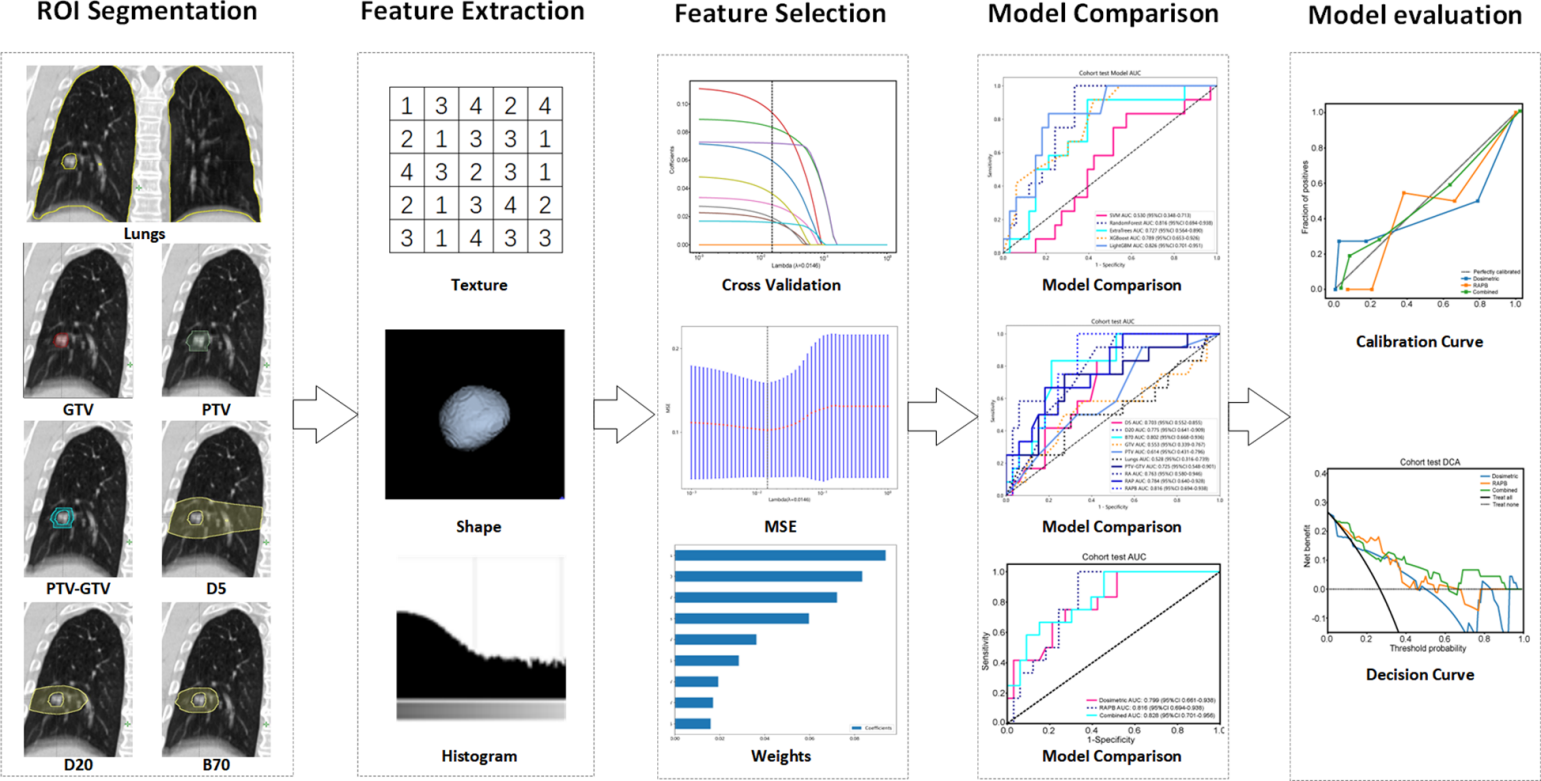
Radiomics workflow of this study. Multiregional segmentation was conducted to generate various ROIs, from which radiomic features were extracted and then selected by ten-fold cross-validation, LASSO, and mean standard error (MSE). Five machine learning algorithms were utilized to construct radiomic models, and their performance was assessed using ROC, calibration, and decision curves analysis. ROI, regions of interest.

After obtaining approval from the Institutional Review Board (IRB), we retrospectively collected data from patients with NSCLC who underwent SBRT at a single center (Huadong Hospital, Fudan University) between 2015 and 2023 as the primary cohort for model establishment, followed by the collection of data from patients at another center (Shanghai Chest Hospital, Shanghai Jiao Tong University School of Medicine) using the same procedure as the test cohort for the external validation of the model. The patients were treated with 6 MV X-rays, receiving a prescribed dose ranging from 48 to 75 Gy administered in 4 to 12 fractions at the isocenter, ensuring that all patients received radiation doses approximately equivalent to 100 Gy of a biologically effective dose with α/β = 10. We collected medical records, CT images, and radiotherapy plans from the databases for analysis. The exclusion criteria were (1) incomplete medical records (2); follow-up less than 6 months after SBRT (3); poor-quality CT images before treatment; and (4) a history of prior thoracic radiotherapy. There were 144 participants in the primary cohort and 45 participants in the test cohort. The entire workflow of our radiomic analysis is detailed in **Figure 1**, providing a thorough overview of the methodologies applied in this research.

### 2.2 CT imaging and treatment planning

CT simulation was conducted using a 4-dimensional computed tomography (4D-CT) acquisition protocol (SOMATOM Definition AS, Siemens Healthineers Corporation, Germany) 3 to 7 days before SBRT in the two cohorts. The CT scans for all patients were conducted using the following imaging parameters: voltage at 120 kV, tube current at 26-140 mAs, pixel size of 0.98 mm × 0.98 mm, and slice thickness of 2-3 mm (13, 18). Subsequently, reconstructed images with a slice thickness of 1 mm were generated and imported into the Pinnacle (v9.1, Philips Medical Systems, USA) or Eclipse (v8.6, Varian Medical Systems, USA) treatment planning system (TPS) workstation for contouring and treatment planning purposes. The GTV was delineated on each of the ten-phase CT imaging series using a lung window setting without any expansion. Then, the ITV was calculated by summing the GTVs from all respiratory phases. To account for daily motion variations, the PTV was generated by expanding the ITV with margins of 3 mm in the posteroanterior/lateral planes and 5 mm in the craniocaudal plane (13, 19). All patients received linear accelerator therapy (Trilogy/Vital Beam/Edge, Varian Medical Systems, USA) and all cone beam computed tomography (CBCT) images were reviewed online by the attending physician to verify tumor locations and correct errors before each fraction. The dose distribution was calculated by Collapsed cone Convolution Superposition (CCCs) or Acuros XB algorithm on the TPS, with the grid size being 2.5 mm × 2.5 mm× 2.5 mm in all three dimensions.

Following the completion of treatment, patients underwent monthly follow-up assessments for a duration of 6 months, after which follow-up appointments were scheduled every 3 months. During routine follow-up, based on the clinical symptoms, signs, and imaging findings of the patients, RP was graded by two physicians according to the Common Terminology Criteria for Adverse Events 5.0 (CTCAE 5.0). If there was any difference, it was determined by a third joint consultation. RP of grade ≥2 is considered SRP.

### 2.3 Clinical parameters and DVH metrics

In our prior research, lung BED was identified as an independent predictor for SRP. In this study, employing the same procedure, we converted physical doses to BED values using the following formula derived from the linear-quadratic (LQ) model: BED (Gy) = n*d* [1 + *d*/(α/β)], where *n* and *d* are the number and size of the dose fractions, and the ratio of α/β is assumed as 10Gy for normal lung tissue, and then derived V_BEDx_ (x ranges from 10 to 200 in intervals of 10) from DVH, representing the percentage of normal lung volume receiving a dose over x Gy of BED. Furthermore, we gathered additional clinical and dosimetric parameters as analytical variables, including sex, age, tumor location, histology, tumor diameter, GTV, PTV, Eastern Cooperative Oncology Group performance status (ECOG PS), chronic obstructive pulmonary disease (COPD), V_5_, V_20_, MLD, and tumor BED. The clinical and DVH metrics for modeling were evaluated using univariate and multivariate analyses.

### 2.4 Regions of interest definition and data preprocessing

**Figure 2:**
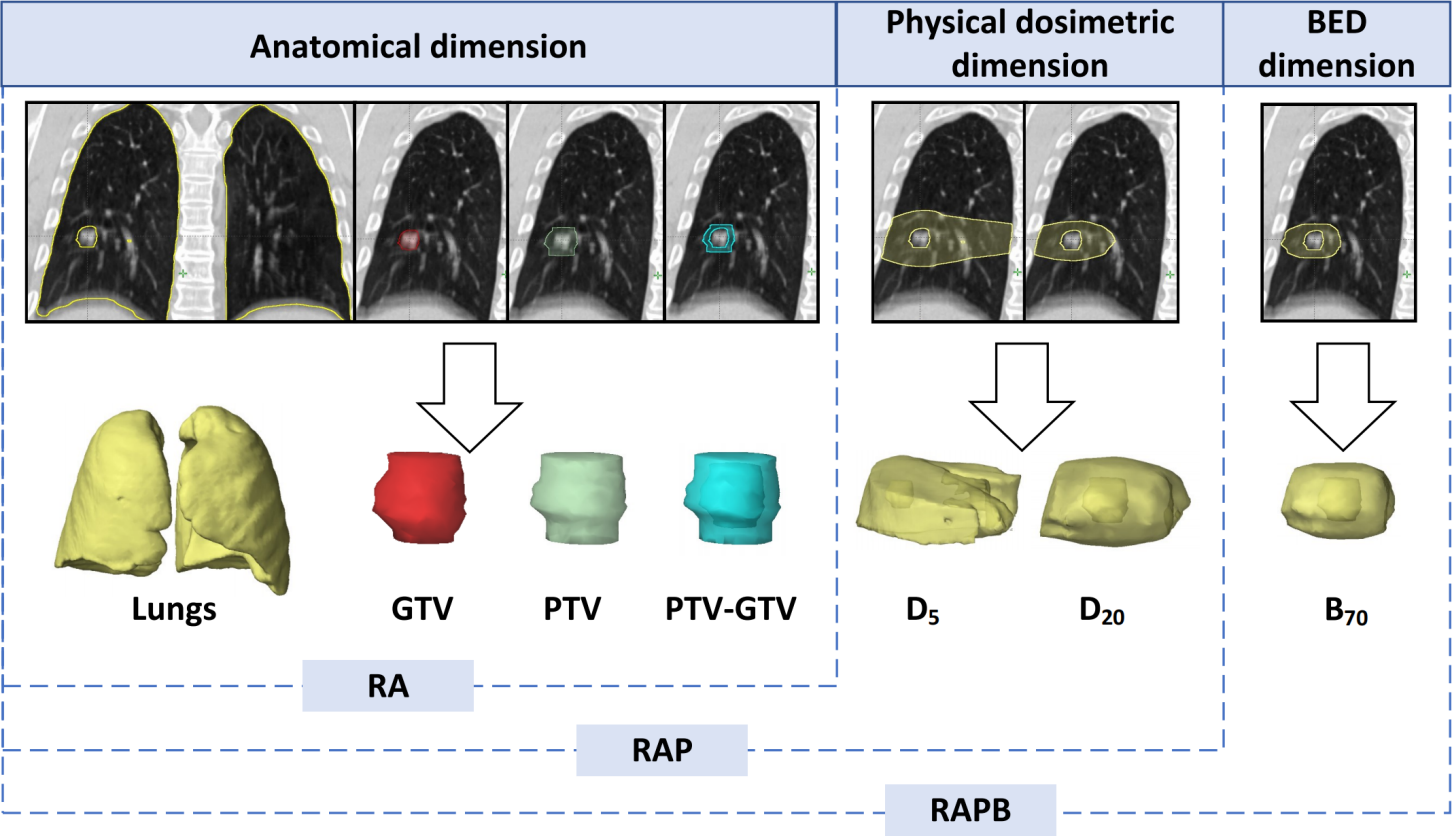
Illustration of the multiregional segmentation. The upper row shows representative coronal CT images of 7 ROIs spanning 3 dimensions, with the corresponding VOIs below from left to right: Lungs, GTV, PTV, PTV-GTV, D5, D20, B70. BED, biologically effective dose; GTV, gross tumor volume; PTV, planning target volume; RA, regions of anatomical dimension; RAP, regions of anatomical dimension combined with physical dosimetric dimension; RAPB, regions of anatomical dimension combined with physical dosimetric and biologically effective dose dimension.

The planning CT series of average image set from the 4DCT, along with corresponding RT structure delineations, were extracted from TPS in the standard Digital Imaging and Communications in Medicine (DICOM) format. Subsequently, the initial bilateral lung outlines, both manually and automatically generated, underwent meticulous scrutiny by a seasoned radiation oncologist. Following this, two additional radiation oncologists independently evaluated the lung organ segmentation. Any disparities were resolved through direct consultation involving all three authors. The GTV and PTV, as previously delineated in the treatment planning section, denote the ROIs. Specifically, PTV-GTV represents the discrepancy between the PTV and GTV, while Lungs signifies the bilateral lung excluding the GTV. Additionally, D_5_ and D_20_ designate the volumes of normal lung tissue receiving doses of 5 Gy and 20 Gy, respectively. B_70_ denotes the volume of normal lung tissue encompassed by a biologically effective dose of 70 Gy with an α/β ratio of 3. To further validate whether increasing the dimensions of ROIs can enhance the performance of radiomic models, this study combined ROIs across three dimensions, which were named as follows: anatomical dimension, designated as RA, comprising GTV, PTV, PTV-GTV, and Lungs; anatomical dimension combined with physical dosimetric dimension, termed RAP, including GTV, PTV, PTV-GTV, Lungs, D_5_, and D_20_; anatomical dimension combined with physical dosimetric and biological equivalent dose dimension, labeled as RAPB, encompassing GTV, PTV, PTV-GTV, Lungs, D_5_, D_20_, and B_70_ (**Figure 2**).

Volumes of interest (VOIs) often exhibit variability in voxel spacing, a phenomenon attributed to the use of diverse scanners or acquisition protocols. Voxel spacing is defined as the physical distance between adjacent pixels in an image. To mitigate this variability, our study implemented spatial normalization, adopting a standardized resampling approach with a fixed resolution of 1 mm×1 mm×1 mm. This method was consistently applied across all experiments to ensure uniformity in voxel spacing.

### 2.5 Feature extraction and selection

In this research, we used the PyRadiomics tool (version 3.0.1) to extract radiomic features (Supplementary Materials A) from ten different regions: GTV, PTV, PTV-GTV, Lungs, D_5_, D_20_, B_70_, RA, RAP, and RAPB. The details of the tool can be found at http://pyradiomics.readthedocs.io.

To achieve standardization and a normal distribution, all features were normalized using the Z score method. The significance of each imaging feature was determined using the *t* test, with only those features exhibiting a *p* value less than 0.05 considered for further analysis. High-repeatability features were rigorously analyzed using Pearson’s correlation coefficient, as depicted in **Figure 1**. This process aimed to identify features that demonstrated a high degree of correlation. When the correlation coefficient between any two features exceeded 0.9, only one feature was retained to avoid redundancy. A greedy recursive deletion strategy was implemented to optimize the feature representation, systematically removing the most redundant feature in each iteration. Additionally, we utilized the minimum redundancy maximum relevance (mRMR) model to further refine the feature set, limiting it to ten (one-tenth of the sample size), thereby reducing the feature count and preventing overfitting.

The final feature selection for the radiomics model construction was performed using the least absolute shrinkage and selection operator (LASSO) regression model, which was tailored to address three distinct tasks. LASSO effectively minimizes regression coefficients, rendering many irrelevant feature coefficients as zero, dependent on the regularization weight λ. The optimal λ value was determined through 10-fold cross-validation, following the minimum criterion, which involves selecting the λ associated with the lowest mean standard error.

### 2.6 Model building and calibration

This study established three models in total: a radiomic model, a clinical model, and a combined model integrating both. For the radiomics model, after feature selection using LASSO, we utilized various machine learning models to construct the risk model. These included linear models, support vector machines (SVMs), and tree-based models, such as random forest (RF) and LightGBM. We conducted thorough comparisons of each model’s performance. To assess the efficacy of the multiregional model, we performed feature fusion on the features extracted from each single region, resulting in a set of fused features. These features were then applied to the multiregional model to compare the performance differences between multiregional fusion and single-regional approaches. With respect to the dosimetric model, we established the model by numerically mapping all clinical and dosimetric parameters (mentioned in Section 2.4) and selecting the most significant ones. Finally, to enhance the clinical applicability, we integrated the radiomic and dosimetric models, resulting in a combined model. The diagnostic effectiveness of our proposed method was evaluated in the test cohort by constructing receiver operating characteristic (ROC) curves. Calibration curves were also generated to assess the calibration performance of the radiomic model. The Hosmer‒Lemeshow (HL) goodness-of-fit test was applied to evaluate its calibration ability. In addition, decision curve analysis (DCA) was utilized to determine the clinical utility of the predictive models.

### 2.7 Statistical analysis

The 144 datasets in the primary cohort were randomly divided, with 70% of the samples allocated to the training group and the remaining 30% to the internal validation group. Additionally, 45 datasets from an external center were used as a test set. To compare the clinical and dosimetric characteristics of patients, various statistical tests were conducted, including the independent sample *t* test, Mann‒Whitney U test, and chi‒squared test, each tailored to the type of variable analyzed. The chi‒squared test was used for categorical variables. To identify significant clinical and dosimetric features, both univariate and stepwise multiple regression analyses were performed for feature selection. Statistical analyses were conducted using Python version 3.7.12 and SPSS version 19.0. Additionally, the development of machine learning models was conducted using the scikit-learn version 1.0.2 interface. Variables with *p* < 0.05 were considered to indicate statistical significance.

## 3 Results

### 3.1 Patient baseline characteristics

**Table 1** summarizes the characteristics of the study participants from the two institutions. The median follow-up time for the patients in our study was 15.3 months. During the follow-up, in the training and validation cohorts, 21 instances of SRP following SBRT was documented, yielding an incidence proportion of 14.6%. In the external validation cohort, 14 cases of SRP were recorded, corresponding to an incidence proportion of 26.7%. No grade 4 or 5 RP was observed. Univariate analysis revealed that none of the baseline clinical characteristics demonstrated statistical significance in relation to the risk of SRP (**Supplementary Materials B Table S1**). Among the tumor-related and dosimetric factors, diameter, PTV, fractions, MLD, V_5_, V_20_, and V_BED10−200_ exhibited significant correlations with SRP (*p* < 0.01), leading to their inclusion in the subsequent multivariate analysis, while GTV, dose per fraction, total dose, and tumor BED did not show significant associations. The subsequent multivariate analysis identified V_BED70_ as the sole significant factor, consistent with previous report (OR=4.84, *p* < 0.001) (13).

**Table 1:** Demographic and disease characteristics of the enrolled patients.

Characteristics	Overall (n=144) Counts (%) or Mean ± SD	Training (n=100) Counts (%) or Mean ± SD	Validation (n=44) Counts (%) or Mean ± SD	*P* value	Testing (n=45) Counts (%) or Mean ± SD
Age	75.5 (39–94)	76 (41–94)	71 (39–88)	0.070	64 (33–78)
Gender				0.082	
Male	86 (59.7)	64 (64.0)	22 (50.0)		12 (26.7)
Female	58 (40.3)	36 (36.0)	22 (50.0)		33 (73.3)
ECOG PS				0.154	
0	26 (18.1)	14 (14.0)	12 (27.3)		6 (13.3)
1	73 (50.7)	54 (54.0)	19 (43.2)		30 (66.7)
2	45 (31.3)	32 (32.0)	13 (29.5)		9 (20.0)
COPD				0.328	
No	97 (67.4)	69 (69.0)	28 (63.6)		24 (53.3)
Yes	47 (32.6)	31 (31.0)	16 (36.4)		21 (46.67)
Histology				0.696	
Adenocarcinoma	38 (26.4)	27 (27.0)	11 (25.0)		16 (35.6)
Squamous carcinoma	85 (59.0)	57 (57.0)	28 (64.6)		25 (55.6)
Others	21 (14.6)	16 (16.0)	5 (11.4)		4 (8.8)
Tumor Location				0.942	
RUL	39 (27.1)	29 (29.0)	10 (22.7)		10 (22.2)
RML	14 (9.7)	10 (10.0)	4 (9.1)		6 (13.3)
RLL	25 (17.4)	17 (17.0)	8 (18.2)		3 (6.7)
LUL	46 (31.9)	31 (31.0)	15 (34.1)		19 (42.2)
LLL	20 (13.9)	13 (13.0)	7 (15.9)		7 (15.6)
Diameter (mm)	30.5 ± 13.5	30.9 ± 13.3	29.4 ± 13.9	0.520	30.6 ± 12.1
GTV (cm^3^)	11.1 ± 15.6	11.5 ± 16.7	10.3 ± 12.9	0.680	6.9 ± 7.5
PTV (cm^3^)	25.8 ± 25.3	26.1 ± 24.7	25.0 ± 26.9	0.811	26.5 ± 18.2
Dose per fraction (Gy)	6.0 (6.0-12.0)	6.0 (6.0-12.0)	6.0 (6.0-10.0)	0.196	10.0 (7.5-12.5)
Fractions (Fx)	10.0 (4.0-12.0)	10.0 (4.0-10.0)	10.0 (5.0-12.0)	0.130	5.0 (4.0-8.0)
Total dose (Gy)	59.8 ± 3.8	59.9 ± 3.4	59.7 ± 4.5	0.750	51.8 ± 3.9
Tumor BED (Gy)	100.7 ± 6.5	101.0 ± 6.6	100.0 ± 6.5	0.401	102.6 ± 4.4
MLD (cGy)	323.6 ± 158.8	322.7 ± 145.9	325.7 ± 186.8	0.917	301.12 ± 114.9

BED, biologically effective dose; COPD, chronic obstructive pulmonary disease; ECOG PS, Eastern Cooperative Oncology Group performance status; GTV, gross tumor volume; LLL, left lower lobe; LUL, left upper lobe; MLD, mean lung dose; PTV, planning target volume; RLL, right lower lobe; RML, right middle lobe; RUL, right upper lobe; SD, standard deviation.

### 3.2 Feature selection

**Figure 3:**
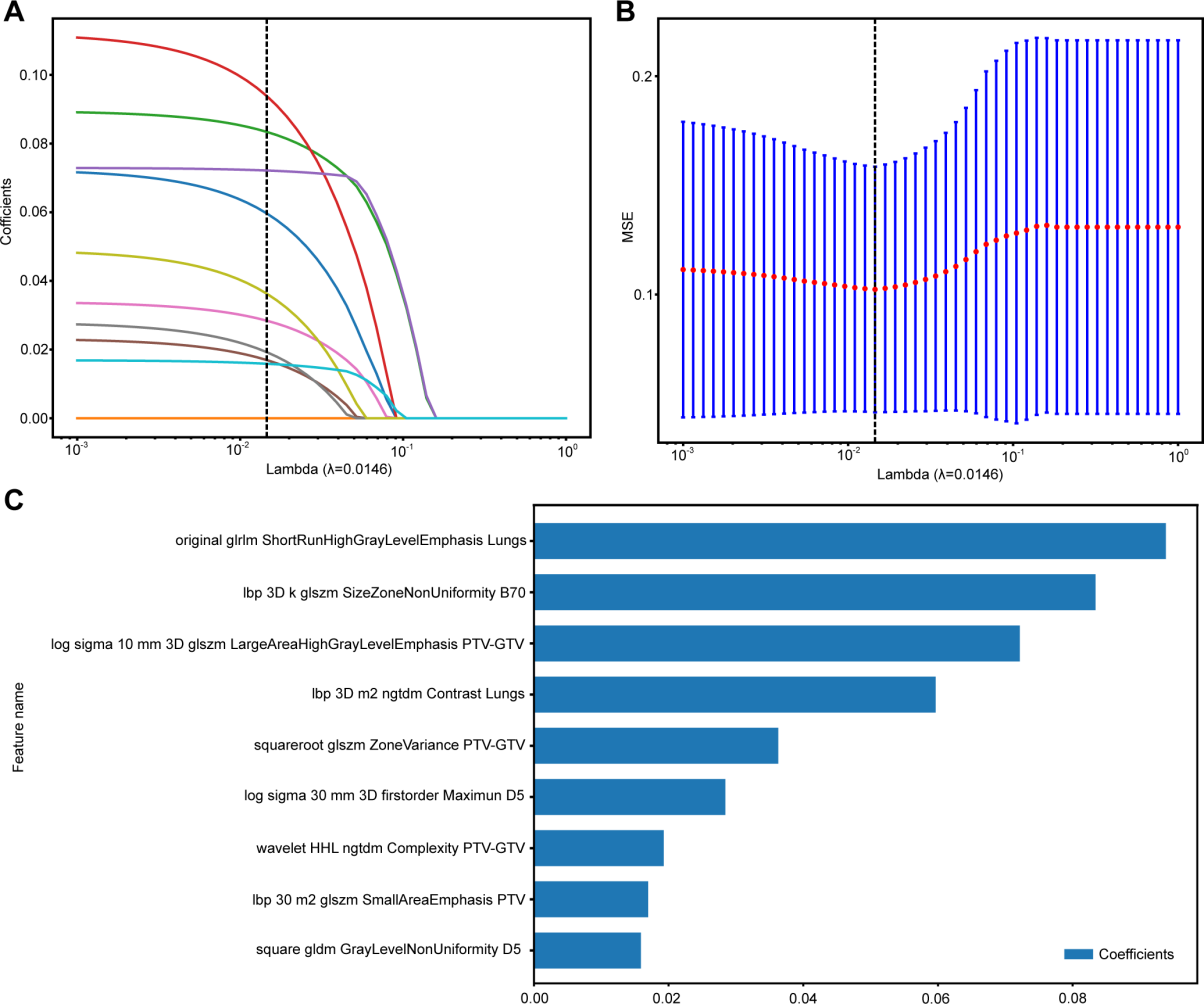
The process of feature selection for RAPB model using the LASSO regression model. **(A)** LASSO coefficients for a range of λ values, with vertical dashed lines indicating the features number at the optimal λ value of 0.0146. **(B)** Optimal λ values are chosen based on 10-fold cross-validation and MSE. **(C)** The selected radiomics features and their coefficients. MSE, mean standard error.

A comprehensive set of 1,834 handcrafted radiomic features was extracted from each plan CT image for each region, resulting in the cumulative acquisition of 18,340 features by combining the radiomic features from 10 different regions. These features were divided into three primary categories: shape, first-order, and texture. Specifically, the dataset included 14 shape features and 360 first-order features, along with a diverse array of texture features. The distribution of these handcrafted features across different categories was visually represented in **Supplementary Materials B Figure S1**. In the final stage of feature analysis, features with nonzero coefficients were selected using a LASSO logistic regression model, resulting in 12, 10, 16, 13, 10, 8, 9 features for GTV, PTV, PTV-GTV, Lungs, D_5_, D_20_, B_70_, and 24, 27, 9 features for RA, RAP, and RAPB, ensuring that only the most relevant features were included in our model (**Supplementary Materials A Figures S2**–**S10**). The coefficients derived from this process, along with the MSE from 10-fold validation for the multiregional radiomic signatures of RAPB, are displayed as an example in **Figure 3**. These figures provide a detailed overview of the model’s performance and the significance of each feature within the model.

### 3.3 Construction and validation of machine learning models

During the training phase of this study, specific machine learning models were strategically selected for each region, catering to three clinical tasks. This deliberate model selection aimed to enhance the predictive accuracy for each modality, showcasing our focused approach to optimizing model efficacy. We employed 5-fold cross-validation and utilized the Grid Search algorithm to optimize the hyperparameters of the model. The optimal model parameters were selected based on their performance metrics in the test set. After establishing these hyperparameters, we trained the model using the entire training dataset to enhance its robustness and accuracy. The specific hyperparameters of the final model are detailed in Supplementary Materials A. For comparative analysis, we selected the most proficient machine learning model for each region in the test set. The chosen models include ExtraTrees for B_70_, GTV, and Lungs; RF for D_5_, PTV, RA and RAPB; LightGBM for D_20_ and RAP; and SVM for PTV-GTV. Detailed performance metrics of these models can be found in **Supplementary Material A Figures S2**–**S11** and **Supplementary Tables S2**–**S11**.

### 3.4 Comparison of radiomic models

**Figure 4:**
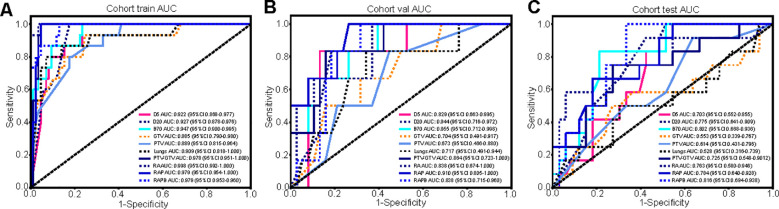
The comparison of the single- and multiregional radiomics models in three cohorts. **(A-C)** The ROC curves of different radiomics models in the training **(A)**, validation **(B)**, and testing **(C)** cohorts, and RAPB model achieved the highest AUC value.

The radiomics features extracted from different ROIs showed different performances, with AUC values ranging from 0.553 to 0.998 in all cohorts. In terms of single regions, B_70_ exhibited better predictive value than the others for predicting SRP [AUC: 0.802 (95% CI: 0.668-0.936)]. Compared with the single-regional models, combination models of different dimensional regions had higher AUC values. Specifically, the RA model outperformed the individual anatomical region models, and the RAP model outperformed the individual physical dosimetry models. In particular, the addition of B_70_ to the RAPB model improved its prediction ability compared to that of RAP alone, suggesting that multiregional fusion radiomic models prevailed any single or other combined regional radiomic models in terms of performance. The effectiveness of the single- and multiregional radiomics models is comprehensively illustrated in **Figure 4** and **Table 2**. The radar chart can be found in **Supplementary Material A Figures S12** (20).

**Table 2:** Prediction performance of different radiomics models for predicting SRP in the training, validation and testing cohorts.

Signature	Accuracy	AUC	95% CI	Sensitivity	Specificity	PPV	NPV	Cohort
D_5_	0.790	0.922	0.868 - 0.977	0.753	1.000	1.000	0.417	Train
D_20_	0.830	0.927	0.878 - 0.976	0.800	1.000	1.000	0.469	Train
B_70_	0.890	0.947	0.900 - 0.995	0.894	0.867	0.974	0.591	Train
GTV	0.770	0.885	0.790 - 0.980	0.741	0.933	0.984	0.389	Train
PTV	0.810	0.889	0.815 - 0.964	0.812	0.800	0.958	0.429	Train
Lungs	0.900	0.909	0.818 - 0.989	0.918	0.800	0.963	0.632	Train
PTV-GTV	0.950	0.978	0.951 - 0.998	0.941	1.000	1.000	0.750	Train
RA	0.960	0.998	0.992 - 0.998	0.953	1.000	1.000	0.789	Train
RAP	0.950	0.979	0.955 - 1.000	0.941	1.000	1.000	0.750	Train
RAPB	0.880	0.979	0.953 - 1.000	0.859	1.000	1.000	0.556	Train
D_5_	0.841	0.829	0.663 - 0.995	0.842	0.833	0.970	0.455	Val
D_20_	0.727	0.844	0.716 - 0.972	0.711	0.833	0.964	0.312	Val
B_70_	0.636	0.855	0.712 - 0.998	0.579	1.000	1.000	0.273	Val
GTV	0.636	0.704	0.491 - 0.917	0.632	0.667	0.923	0.222	Val
PTV	0.568	0.673	0.466 - 0.881	0.526	0.833	0.952	0.217	Val
Lungs	0.636	0.717	0.491 - 0.944	0.605	0.833	0.958	0.250	Val
PTV-GTV	0.818	0.864	0.723 - 1.000	0.816	0.833	0.969	0.417	Val
RA	0.614	0.838	0.674 - 1.000	0.553	1.000	1.000	0.261	Val
RAP	0.750	0.910	0.805 - 1.000	0.711	1.000	1.000	0.353	Val
RAPB	0.705	0.838	0.715 - 0.960	0.658	1.000	1.000	0.316	Val
D_5_	0.578	0.703	0.552 - 0.855	0.424	1.000	1.000	0.387	Test
D_20_	0.622	0.775	0.641 - 0.909	0.515	0.917	0.944	0.407	Test
B_70_	0.778	0.802	0.668 - 0.936	0.758	0.833	0.926	0.556	Test
GTV	0.644	0.553	0.339 - 0.768	0.697	0.500	0.793	0.375	Test
PTV	0.489	0.614	0.431 - 0.796	0.333	0.917	0.917	0.333	Test
Lungs	0.622	0.528	0.317 - 0.739	0.667	0.500	0.786	0.353	Test
PTV-GTV	0.711	0.725	0.548 - 0.902	0.697	0.750	0.885	0.474	Test
RA	0.822	0.763	0.579 - 0.946	0.909	0.583	0.857	0.700	Test
RAP	0.756	0.784	0.640 - 0.929	0.788	0.667	0.867	0.533	Test
RAPB	0.733	0.816	0.694 - 0.938	0.636	1.000	1.000	0.500	Test

SRP, symptomatic radiation pneumonitis; AUC, areas under the curve; CI, confidence interval; PPV, positive predictive value; NPV, negative predictive value; GTV, gross tumor volume; PTV, planning target volume; RA, regions of anatomical dimension; RAP, regions of anatomical dimension combined with physical dosimetric dimension; RAPB, regions of anatomical dimension combined with physical dosimetric and biologically effective dose.

### 3.5 Model comparison, calibration, and DCA

**Figure 5:**
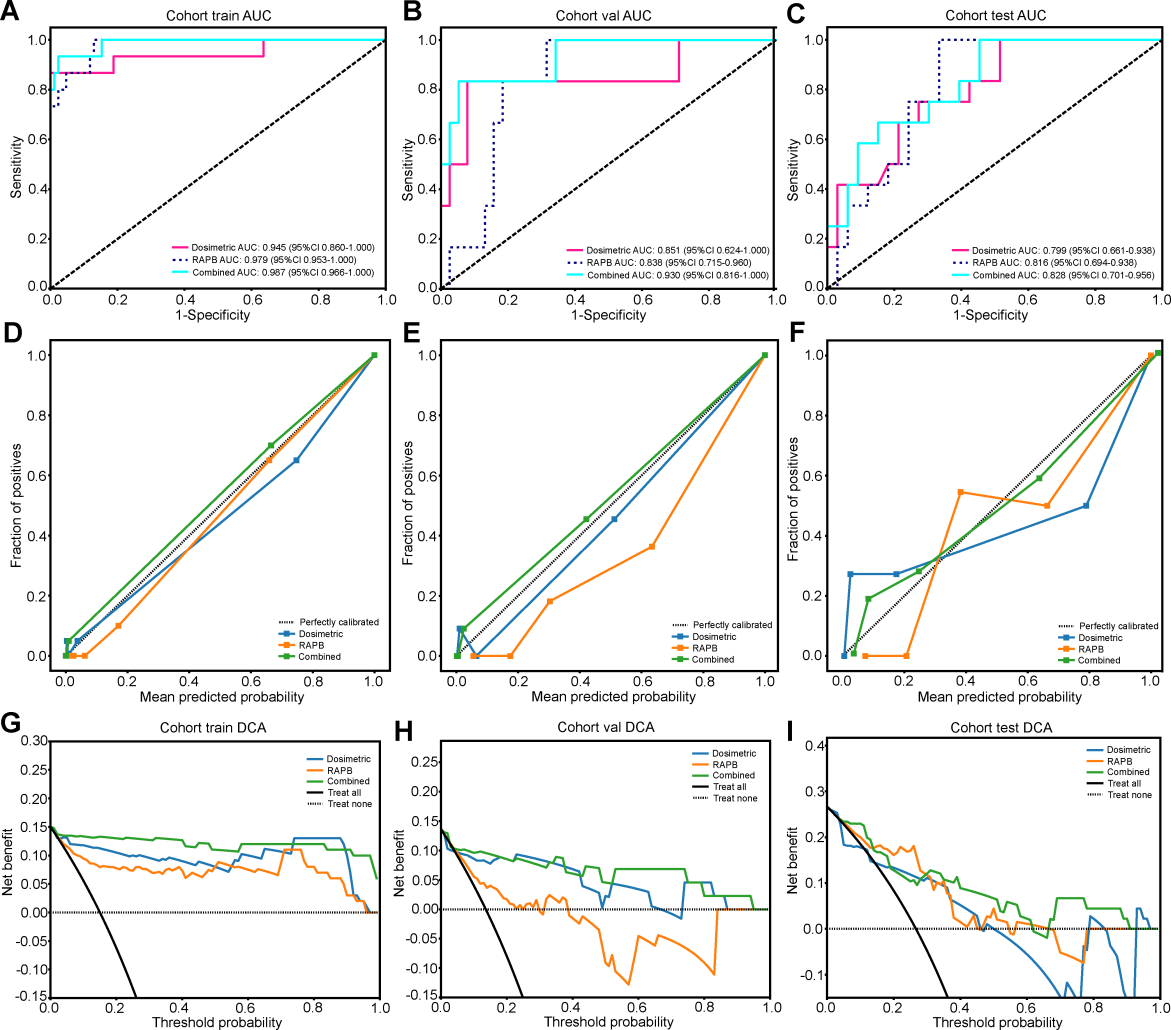
The results and evaluation of SRP prediction models in three cohorts. **(A-C)** ROC curves of the dosimetric model, RAPB model, and Combined model for evaluating SRP differentiation in the training **(A)**, validation **(B)**, and testing **(C)** cohorts. **(D-F)** The calibration curves of three models for evaluating SRP differentiation in the training **(D)**, validation **(E)**, and testing **(F)** cohorts. **(G-I)** The DCA curves of the different models for evaluating SRP differentiation in the training **(G)**, validation **(H)**, and testing **(I)** cohorts.

The predictive performance of combined dosimetric-radiomic features was evaluated in an independent validation cohort. For predicting SRP, models based solely on dosimetric factors (V_BED70_) exhibited reduced performance compared with RAPB radiomic model (dosimetric: AUC = 0.799, 95% CI: 0.661–0.938; RAPB: AUC = 0.816, 95% CI: 0.694–0.938). Based on the training and testing sets, the combined model, which incorporated both dosimetric and RAPB results, demonstrated superior model performance (AUC = 0.828, 95% CI: 0.701–0.956). The ROC curves for each model in the three cohorts are shown in **Figure 5** and **Table 3** (**Supplementary Materials A Figures S13**).

**Table 3:** Prediction performance of Dosimetric model, RAPB model, and Combined model for predicting SRP in the training, validation and testing cohorts.

Model	Accuracy	AUC	95% CI	Sensitivity	Specificity	PPV	NPV	Cohort
Dosimetric	0.970	0.945	0.860 - 1.000	0.800	1.000	1.000	0.966	Train
RAPB	0.880	0.979	0.953 - 1.000	0.933	0.871	0.560	0.987	Train
Combined	0.960	0.987	0.966 - 1.000	0.867	0.976	0.867	0.976	Train
Dosimetric	0.886	0.851	0.624 - 1.000	0.667	0.921	0.571	0.946	Val
RAPB	0.705	0.838	0.715 - 0.960	0.833	0.684	0.294	0.963	Val
Combined	0.909	0.930	0.816 - 1.000	0.667	0.947	0.667	0.947	Val
Dosimetric	0.600	0.799	0.661 - 0.938	0.917	0.485	0.393	0.941	Test
RAPB	0.733	0.816	0.694 - 0.938	0.917	0.667	0.500	0.957	Test
Combined	0.644	0.828	0.701 - 0.956	0.917	0.545	0.423	0.947	Test

SRP, symptomatic radiation pneumonitis; RAPB, regions of anatomical dimension combined with physical dosimetric and biologically effective dose; AUC, areas under the curve; CI, confidence interval; PPV, positive predictive value; NPV, negative predictive value.

In the calibration curve analysis, the HL test measured the discrepancy between the predicted probabilities and the actual outcomes. The lower HL test values were preferred, denoting a closer match between the model’s predictions and the observed results. In this context, the combined model demonstrated remarkable calibration effectiveness. This was reflected in the HL test statistics, which was 0.923 for the training cohort, 0.544 for the validation cohort, and 0.374 for the test cohort (**Figure 5**). These figures, especially the lower values in the validation and test cohorts, suggested a high degree of alignment between the predicted probabilities and the actual outcomes, underscoring the model’s reliability.

## 4 Discussion

Accurate assessment of SRP occurrence risk and identification of high-risk patients are imperative to proactively prevent SRP, thereby ensuring the efficacy and safety of SBRT. The incidence of SRP (around 14.6%) at our institution was similar to that reported in previous studies (9.4-29%) (8, 21, 22). This study confirmed our previous findings that the V_BED70_ of the nontarget lung volume was an independent clinical risk factor, and further revealed that the model integrating multiregional radiomic signatures and dosimetric factors based on V_BED70_ was highly reliable for SRP predicting with better performance than conventional clinical indicators and DVH parameters derived from physical doses. Furthermore, multiregional radiomic models outperformed their single-regional counterparts, notably with the incorporation of the B_70_ area, thereby improving the predictive accuracy of SRP. To the best of our knowledge, this study represents the first utilization of multidimensional and multiregional radiomic features extracted from the BED distribution of the nontarget lung volume, empowered by diverse machine learning algorithms, to predict the risk of SRP following lung SBRT with heterogeneous fractionations in the clinical scenarios.

Various dosimetrics, such as the volume of nontarget lung tissue receiving specific radiation doses (Vx) and the MLD, have been extensively studied for their association with SRP (8, 23, 24). However, the findings have been inconclusive. Liu et al. (8) and Barriger et al. (23) identified MLD and V_20_ as significant risk factors for SRP, while Matsuo et al. (24) suggested that only the PTV, V_20_, and V_25_ were indicative of RP, with other factors such as MLD and various Vx values showing no association. In terms of normal tissue complication probability (NTCP), the lung, as a late-responding tissue (α/β = 3), is highly sensitive to dose fraction variation, characterized by BEDs. Previous studies have demonstrated that the fractionation dose is a significant factor related to the occurrence of RP (14, 25). However, the majority of previous studies used the physical doses applied to lung tissue in the DVH, considering none or few of the variations in the biological effects on normal lung tissue from different SBRT fractionation schemes. Therefore, in this study, in addition to actual physical dose, we incorporated lung BED for dose-risk analysis. In the univariate analysis, an evident association between BED-derived dosimetric variables and SRP was observed in SBRT-treated patients. Further multivariate analysis showed that lung V_BED70_ was an independent risk factor for SRP, whereas previously reported risk factors such as V_5_, V_20_, and MLD did not significantly differ between SRP and SRP-free patients in our study. This is in line with findings in the literature (26).

In radiomic researches, the accuracy of prediction models is largely subjected to the selection of ROIs and feature handling. In our study, the comparative evaluation of single radiomic models using different ROI dimensions revealed that the radiomic model based on the biologically equivalent dose dimension of lung tissue (B_70_ model) had the best performance, with the accuracy improving as the ROI dimensions increased (RAPB > RAP > RA). The results are concordant with those of previous multiregional radiomics investigations (9, 27). In the anatomical dimension, the radiomic features of GTV had the lowest predictive value (AUC: 0.553, 95% CI: 0.339-0.768) without significant correlation with SRP risk in the multiregional radiomic model RAPB, while features from PTV-GTV demonstrated superior performance (AUC: 0.725, 95% CI: 0.548-0.902), in agreement with previous studies (9). The expansion of GTV to PTV to compensate respiratory motion and uncertainties from patient setup, planning and dose delivery inevitably includes a portion of normal lung tissue into the treatment field, contributing to the occurrence of SRP. This highlights the greater representativeness and indicative nature of PTV-GTV features, emphasizing that only the radiomic characteristics of lung tissue itself, rather than those of the tumor, can quantify the spatial microstructure of lung tissues and thus more accurately reflect the sensitivity of normal lung tissue to radiation. Our multidimensional multiregional radiomic approach, which incorporates intrinsic lung tissue characteristics alongside information on physical and biologically equivalent dose distributions, enhances the predictive capability of SRP through complementary advantages, demonstrating the superiority of this method.

The comparative analysis of the dosimetric, RAPB, and combined models across the training, validation, and test cohorts demonstrated certain noteworthy trends. In the training cohort, the combined model of dosimetric and RAPB features performed better than the RAPB model alone, highlighting the benefit of data integration, while during validation, the dosimetric model’s performance significantly decreased, particularly in sensitivity and specificity, whereas the RAPB model remained resilient. Additionally, compared with other models, the DCA curves (**Figure 5**) showed that our combined model yields noticeable benefits. These findings are consistent with previous reports (28, 29). Kong et al. found that the optimal predictive performance (AUC: 0.88) was attained when dosimetric parameters were incorporated into the predictive model using features from lung-PTV (28). Our results demonstrated the advantages of the combined model, which effectively leverages the strengths of both dosimetric and radiomic features to synergistically improve predictive performance. This highlighted the necessity of integrating the features of multiple ROIs and the lung BED dose distribution in predicting the risk of SRP during the process of formulating treatment plans, optimizing personalized dose distribution and closely monitoring for high-risk populations.

To develop specialized machine learning-based radiomic models presents formidable challenges. Notably, to address concerns regarding overfitting and multicollinearity stemming from the vast array of features, we systematically employed Spearman rank correlation, LASSO, and mRMR techniques to reduce dimensionality and identify optimal features, in compliance with the established guidelines for radiomics research (30). For robustly testing the medical hypotheses regarding the comparative predictive values of different feature sets, we conducted a comparative analysis of multiple machine learning algorithms to determine the algorithm most suitable to our dataset. It was proved that the AUC values of the machine learning algorithms in the RAPB model were notably higher than those of the other models (SVM, ExtraTrees, XGBoost, and LightGBM) in their corresponding cohorts, suggesting the strong predictive capability and reliability of the RF model. Therefore, based on the AUC and CI values, the RF model was employed for the consecutive analysis. All of the prediction models underwent simultaneous training and testing using identical technical principles in an external cohort, ensuring optimal comparability and clinical applicability. This approach significantly improved the performance in predicting the occurrence of SRP.

Nonetheless, this study has certain limitations. Firstly, the sample size is relatively small and all data used for model development were retrospective, which inevitably compromises the reliability to some extent due to susceptibility to biases, including patient selection, clinical and radiological information, and other confounding factors. To address these issues, we meticulously utilized objective records and quantitative indicators as analytic variables. Furthermore, stratified analysis and multivariate logistic regression analysis were employed to mitigate confounding bias. Moreover, an external validation set using the same inclusion and exclusion criteria was assembled to validate the established model. Certainly, future well-designed prospective multicenter studies are anticipated to generate unbiased dataset to further validate and optimize the model. Secondly, this study included only clinical factors, dosimetric parameters, and imaging features, without considering other factors affecting SRP, for instance inflammatory biomarkers and pulmonary function parameters. Additionally, although previous studies indicated that reduced cardiopulmonary function exacerbates radiation-induced lung toxicity, this study did not analyze the relationship between cardiac dose and radiation pneumonitis in terms of dosimetric risk factors.

## 5 Conclusion

This study employs various machine learning methods to establish and validate a predictive model for SRP following various SBRT fractionation schemes. The results demonstrate that the combined model integrating multiregional radiomic signatures and lung tissue BED-based dosimetric parameters may be promising for SRP prediction. Specifically, the inclusion of region B_70_, which is derived from the area of lung biologically equivalent dose, significantly enhances the predictive performance of the multiregional radiomic model. For performance robustness and reliability, the combined model needs further validation and optimization using larger-scale retrospective data and in prospective trials.

## Data Availability

The original contributions presented in the study are included in the article/**Supplementary Material**. Further inquiries can be directed to the corresponding authors.
