# Supplementary material for: Development and validation of a lung biological equivalent dose-based multiregional radiomic model for predicting symptomatic radiation pneumonitis after SBRT in lung cancer patients
Source: Front Oncol. 2024 Dec 6;14:1489217. doi: 10.3389/fonc.2024.1489217 (PMC11659668; doi:10.3389/fonc.2024.1489217)
Supplement: Supplementary file 1 [file DataSheet1.docx]

Supplementary Material A

1. **Hyperparameters of Machine Learning Algorithms**

In this investigation, a variety of machine learning models were employed, each optimized with specific hyperparameters for enhanced performance. The SVM (Support Vector Machine) model incorporated an RBF (Radial Basis Function) kernel. The Random Forest Classifier was configured with 21 estimators, a maximum depth of three levels, and necessitated a minimum of two samples for splitting nodes. The XGBoost (eXtreme Gradient Boosting) Classifier was set up with five estimators, a constrained depth of two, and a minimal child weight of 0.1. The LightGBM (Light Gradient Boosting Machine) Classifier utilized 16 estimators, also limited to a depth of two. Lastly, the Extra Trees Classifier was designed with 20 estimators, a depth ceiling of two, and required a minimum of two samples for node division, ensuring reduced model complexity and overfitting.

1. **Feature extraction**

In this research, we extracted radiomic features aligned with each ROI. The handcrafted features were categorized into three distinct groups: (I) geometry, which includes the three-dimensional shape characteristics of the ROI; (II) intensity, which represents the first-order statistical distribution of voxel intensities within the ROI; and (III) texture, which details the patterns or the second- and higher-order spatial distributions of intensities. A range of methods, such as the gray-level co-occurrence matrix (GLCM), gray-level run length matrix (GLRLM), gray-level size zone matrix (GLSZM), and neighborhood gray-tone difference matrix (NGTDM), were used for texture feature extraction. All features were extracted using the PyRadiomics tool (version 3.0.1), adhering closely to the feature definitions established by the Imaging Biomarker Standardization Initiative (IBSI). This study applied consistent feature extraction methodologies to derive features from ten different regions: GTV, PTV, PTV-GTV, Lungs, D5, D20, B70, RA, RAP, and RAPB.

Supplementary Material B

Table S1. Univariable and multivariable analysis of dosimetric features.

| Characteristics | Non-SRP(n=123)  Counts(%)/Mean ± SD | SRP(n=21)  Counts(%)/Mean ± SD | *P* value  univariable | *P* value  multivariable |
| --- | --- | --- | --- | --- |
| Age | 76 (41-94) | 72 (39-85) | 0.479 | / |
| Gender |  |  | 0.825 | / |
| Male | 73(59.3) | 13(61.9) |  |  |
| Female | 50(40.7) | 8(38.1) |  |  |
| ECOG PS |  |  | 0.085 | / |
| 0 | 25(20.3) | 1(4.8) |  |  |
| 1 | 58(47.2) | 15(71.4) |  |  |
| 2 | 40(32.5) | 5(23.8) |  |  |
| COPD |  |  | 0.618 | / |
| No | 84(68.3) | 13(61.9) |  |  |
| Yes | 39(31.7) | 8(38.1) |  |  |
| Histology |  |  | 0.312 | / |
| Adenocarcinoma | 33(26.8) | 5(23.8) |  |  |
| Squamous carcinoma | 70(56.9) | 15(71.4) |  |  |
| Others | 20(16.3) | 1(4.8) |  |  |
| Tumor Location |  |  | 0.110 | / |
| RUL | 29(23.6) | 10(47.7) |  |  |
| RML | 14(11.4) | 0(0) |  |  |
| RLL | 21(17.1) | 4(19.0) |  |  |
| LUL | 42(34.1) | 4(19.0) |  |  |
| LLL | 17(13.8) | 3(14.3) |  |  |
| Diameter(mm) | 29.6 ± 13.2 | 35.9 ± 14.2 | 0.046 | 0.406 |
| GTV(cm3) | 10.33 ± 15.9 | 15.9 ± 12.7 | 0.132 | / |
| PTV(cm3) | 24.0 ± 25.3 | 36.0 ± 23.5 | 0.041 | 0.211 |
| Single Dose(Gy) | 6.0 (6.0-10.0) | 7.5 (6.0-12.0) | 0.205 | / |
| Fractions(Fx) | 10.0 (5.0-10.0) | 8.0 (4.0-12.0) | 0.015 | 0.055 |
| Total dose(Gy) | 60.1 ± 3.3 | 58.1 ± 5.7 | 0.124 | / |
| Tumor BED(Gy) | 100.6 ± 6.7 | 101.4 ± 5.9 | 0.588 | / |
| MLD(cGy) | 292.8 ± 133.5 | 504.3 ± 177.5 | ＜0.001 | 0.805 |
| V_BED10_ | 9.49 ± 4.82 | 17.30 ± 6.52 | ＜0.001 | 0.770 |
| V_BED20_ | 5.72 ± 13.22 | 11.79 ± 4.37 | ＜0.001 | 0.479 |
| V_BED30_ | 3.92 ± 2.36 | 8.86 ± 3.18 | ＜0.001 | 0.496 |
| V_BED40_ | 2.84 ± 1.77 | 6.85 ± 2.34 | ＜0.001 | 0.408 |
| V_BED50_ | 2.16 ± 1.39 | 5.31 ± 1.79 | ＜0.001 | 0.785 |
| V_BED60_ | 1.70 ± 1.10 | 4.26 ± 1.51 | ＜0.001 | 0.898 |
| V_BED70_ | 1.36 ± 0.89 | 3.48 ± 1.32 | ＜0.001 | ＜0.001 |
| V_BED80_ | 1.11 ± 0.73 | 2.87 ± 1.16 | ＜0.001 | 0.816 |
| V_BED90_ | 0.91 ± 0.61 | 2.39 ± 1.05 | ＜0.001 | 0.746 |
| V_BED100_ | 0.75 ± 0.51 | 2.00 ± 0.94 | ＜0.001 | 0.958 |
| V_BED110_ | 0.61 ± 0.44 | 1.69 ± 0.85 | ＜0.001 | 0.896 |
| V_BED120_ | 0.50 ± 0.38 | 1.42 ± 0.78 | ＜0.001 | 0.763 |
| V_BED130_ | 0.40 ± 0.33 | 1.17 ± 0.74 | ＜0.001 | 0.751 |
| V_BED140_ | 0.32 ± 0.29 | 0.96 ± 0.71 | ＜0.001 | 0.775 |
| V_BED150_ | 0.25 ± 0.25 | 0.79 ± 0.67 | 0.001 | 0.734 |
| V_BED160_ | 0.19 ± 0.21 | 0.64 ± 0.62 | 0.003 | 0.604 |
| V_BED170_ | 0.14 ± 0.18 | 0.52 ± 0.56 | 0.006 | 0.383 |
| V_BED180_ | 0.10 ± 0.15 | 0.42 ± 0.51 | 0.009 | 0.254 |
| V_BED190_ | 0.07 ± 0.13 | 0.33 ± 0.45 | 0.015 | 0.196 |
| V_BED200_ | 0.05 ± 0.11 | 0.25 ± 0.40 | 0.030 | 0.276 |
| V_5_ | 14.01 ± 7.32 | 22.62 ± 8.51 | ＜0.001 | 0.718 |
| V_20_ | 3.37 ± 2.13 | 7.46 ± 3.06 | ＜0.001 | 0.972 |

*Abbreviation: SD: standard deviation; ECOG PS: Eastern Cooperative Oncology Group performance status; COPD: chronic obstructive pulmonary disease; RUL: right upper lobe; RML: right middle lobe; RLL: right lower lobe; LUL: left upper lobe; LLL: left lower lobe; GTV: gross tumor volume; PTV: planning target volume; BED: biologically effective dose. MLD: mean lung dose.*


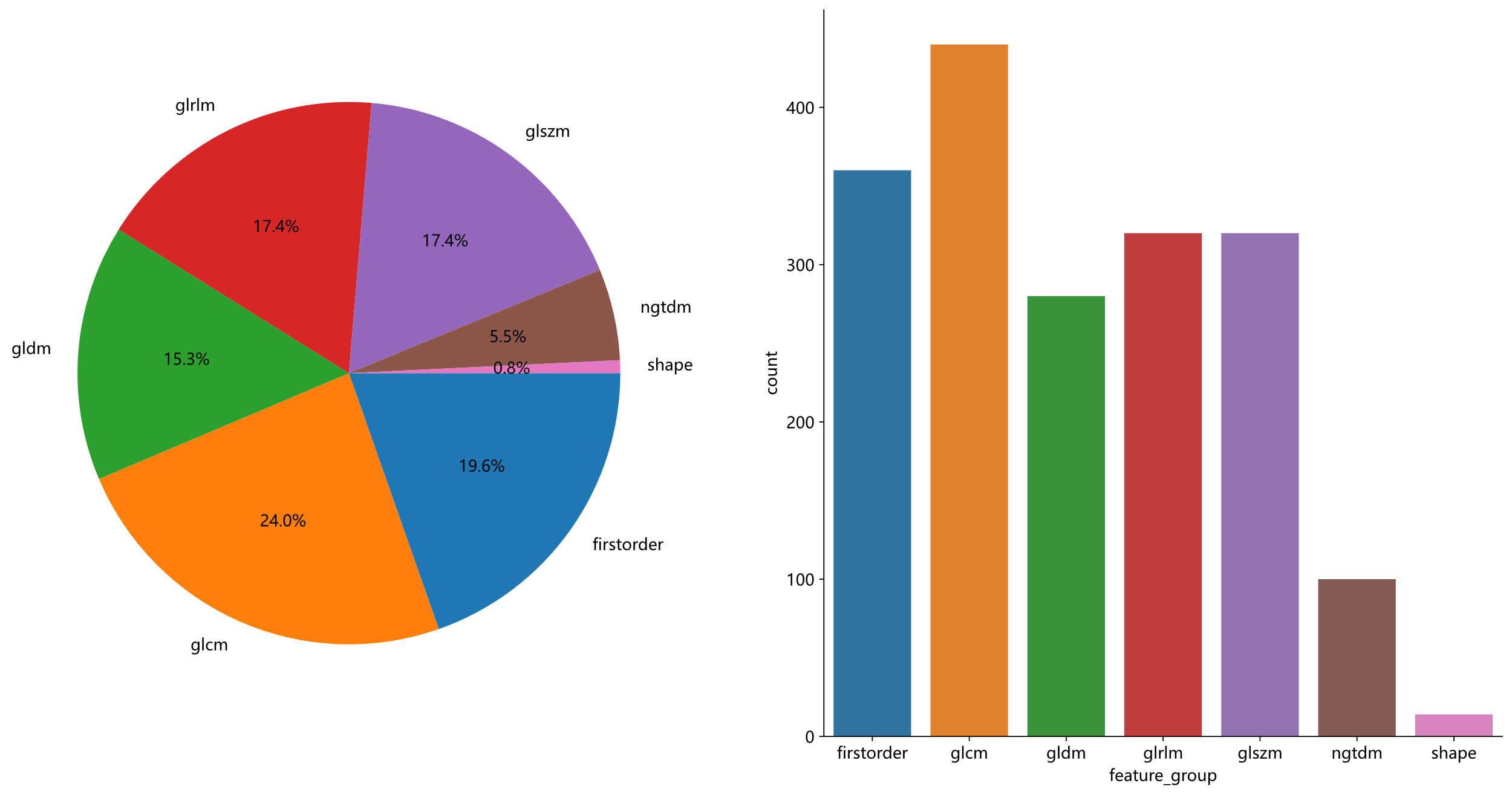


Figure S1. Number and ratio of handcrafted features

*Abbreviation: glcm:gray-level co-occurrence matrix;gldm:gray-level dependence matrix;glrlm:gray-level run length matrix;glszm:gray-level size zone matrix;ngtdm:neighborhood gray-tone difference matrix.*


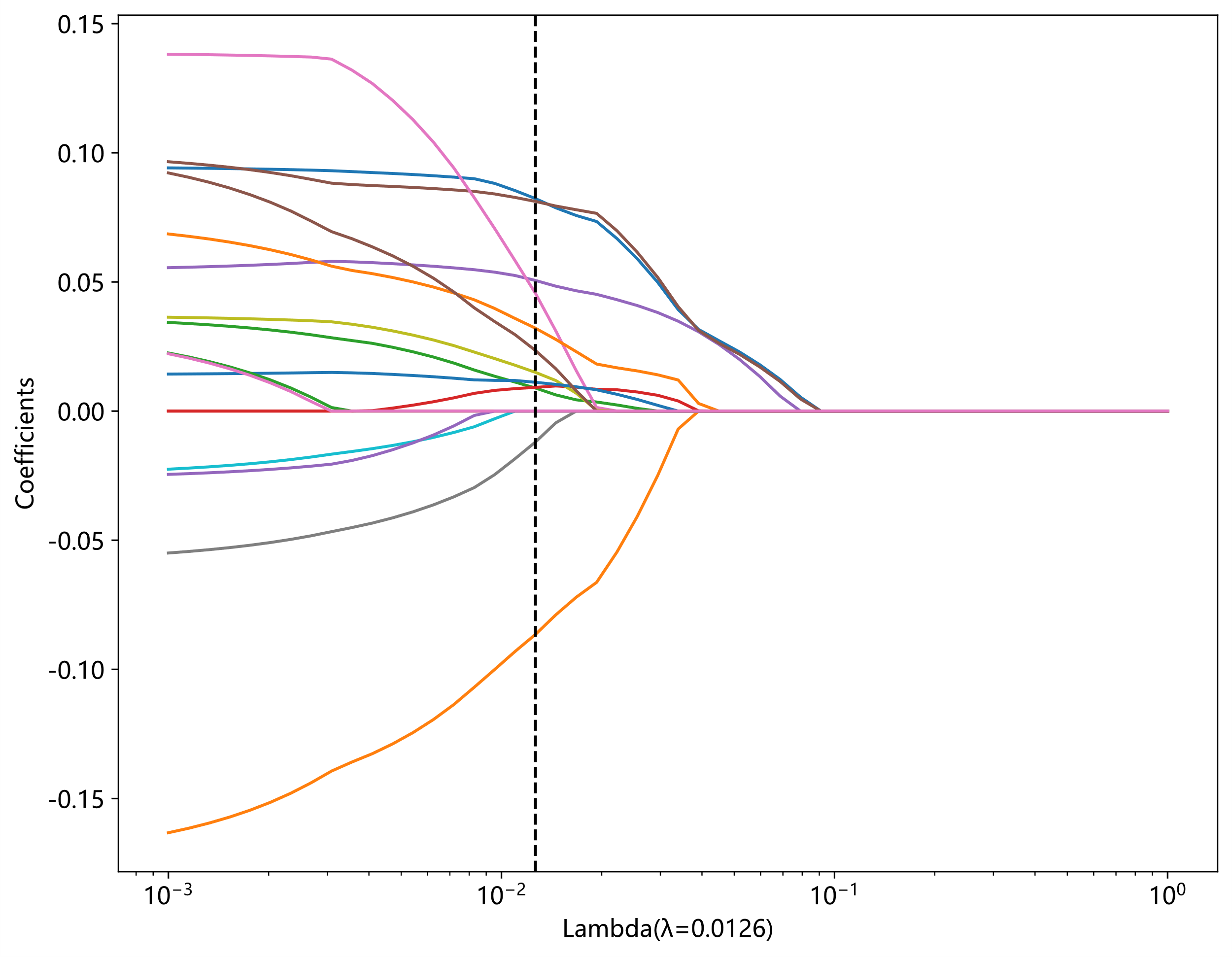


**A**


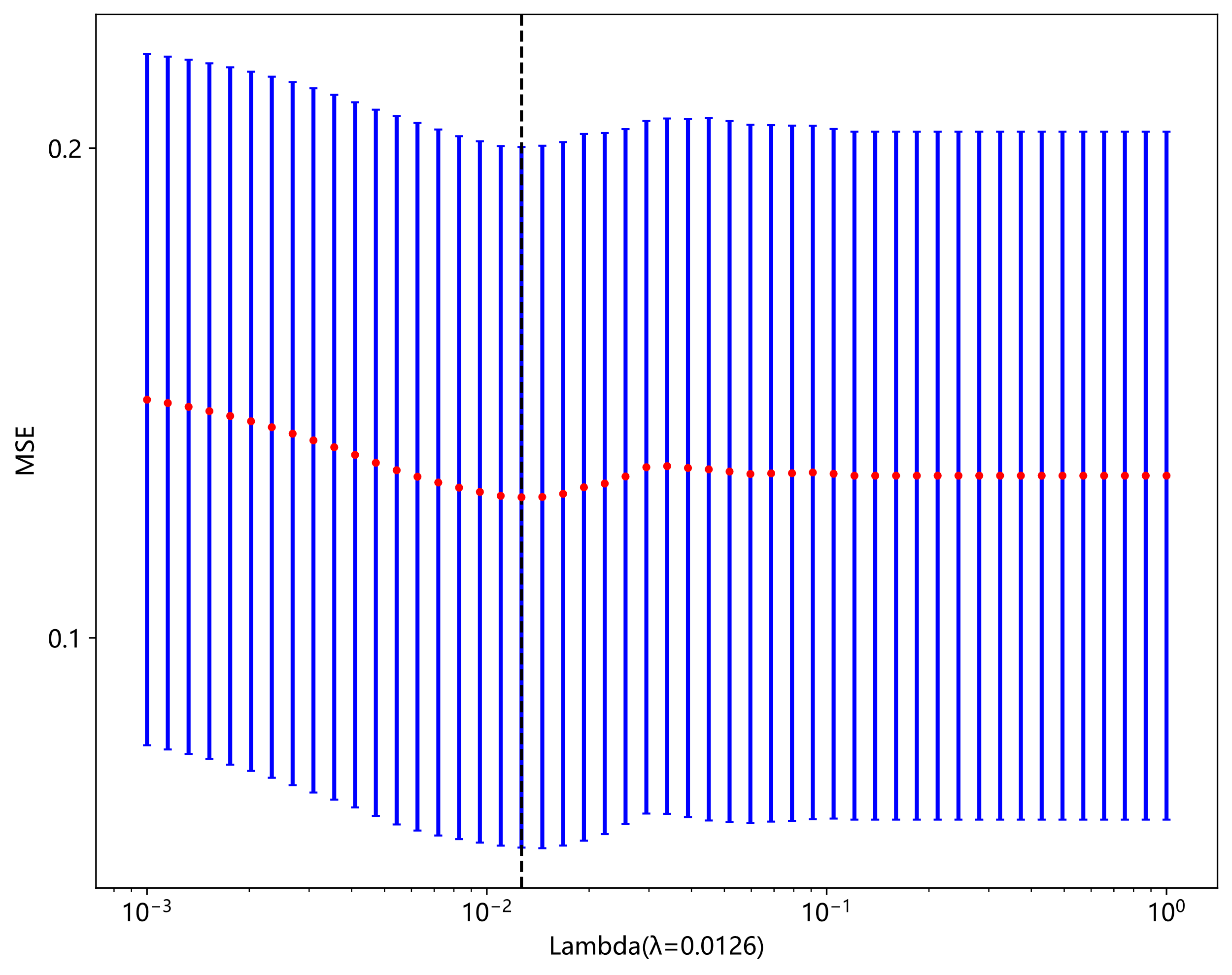


**B**


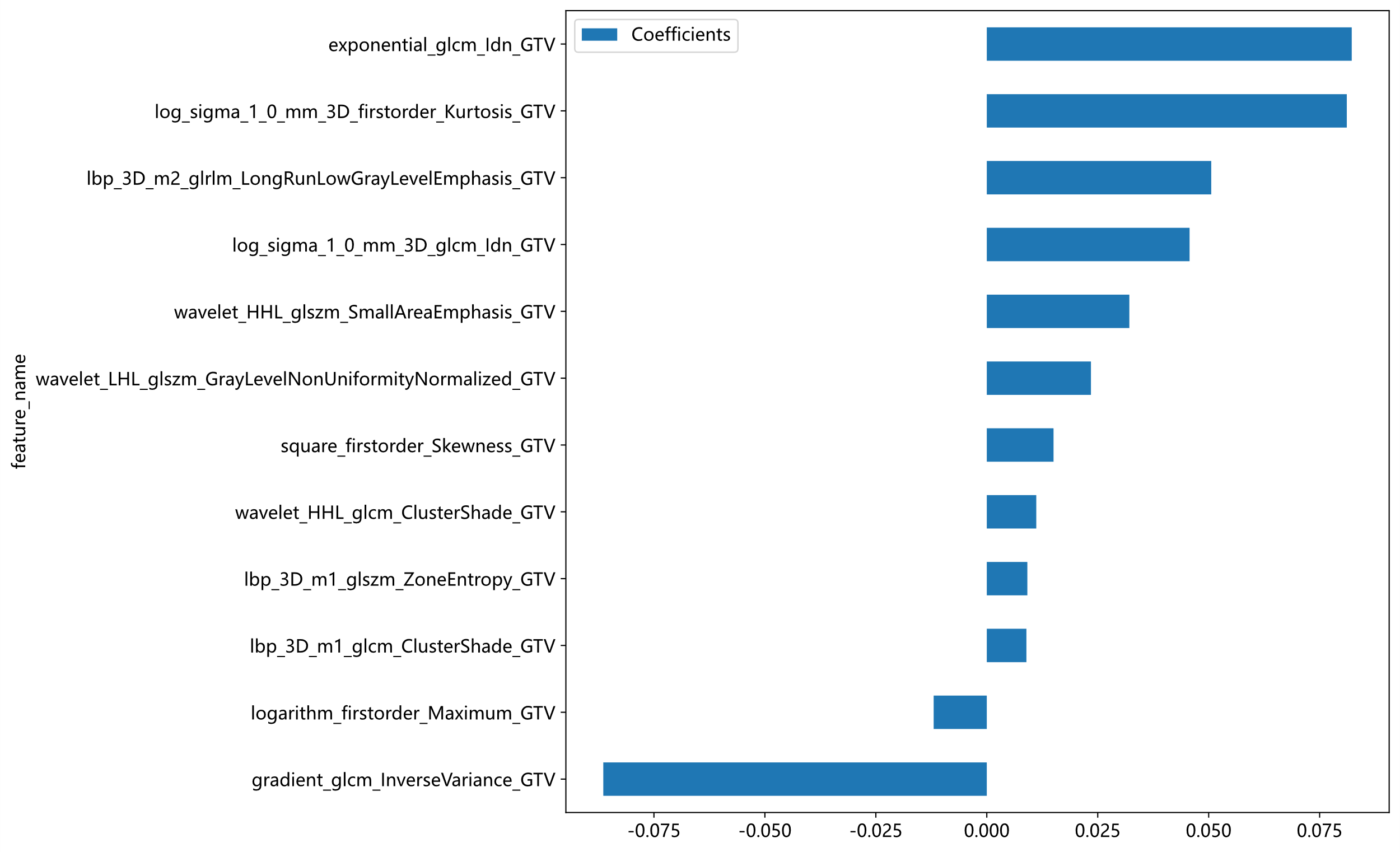


**C**


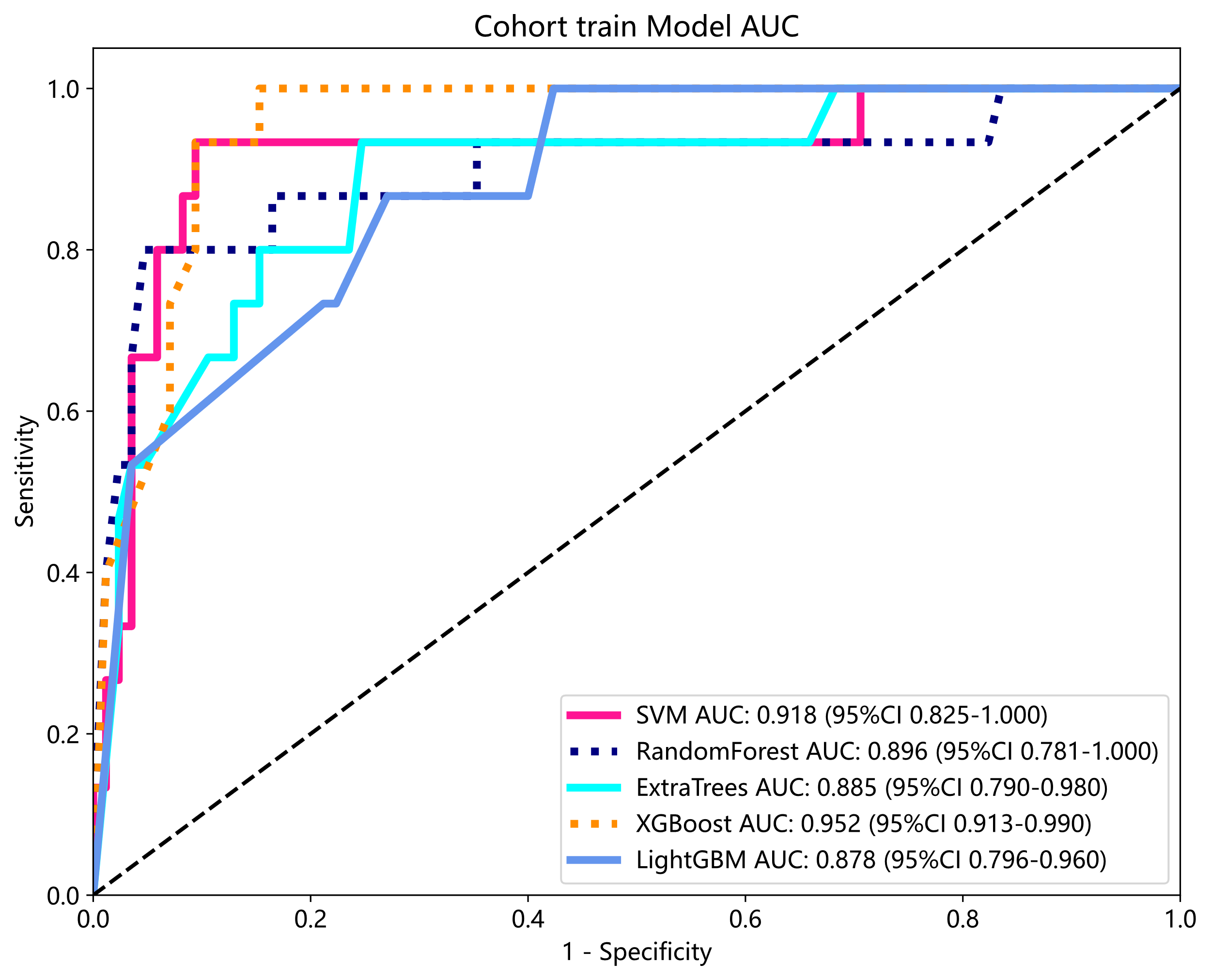


**D**


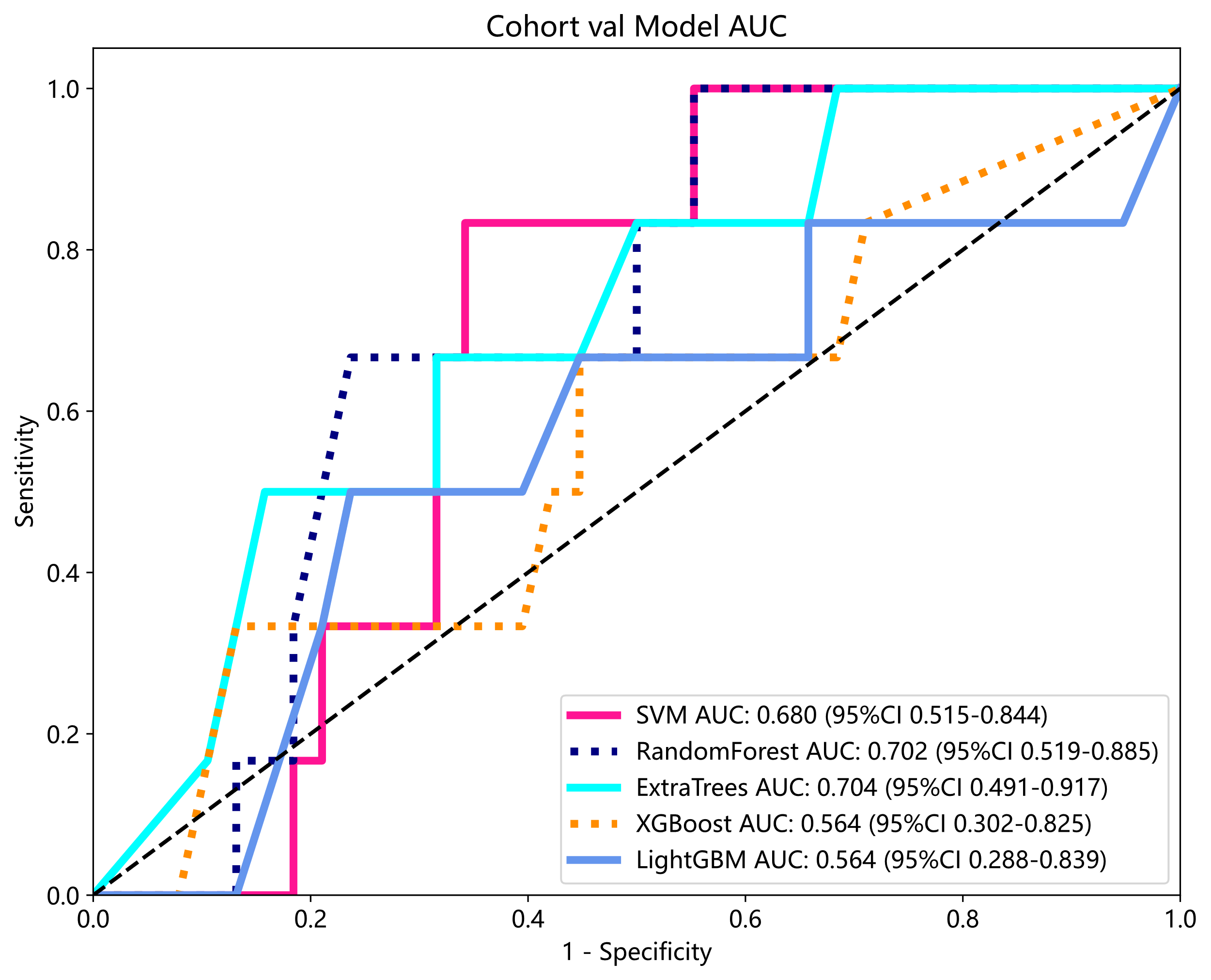


**E**


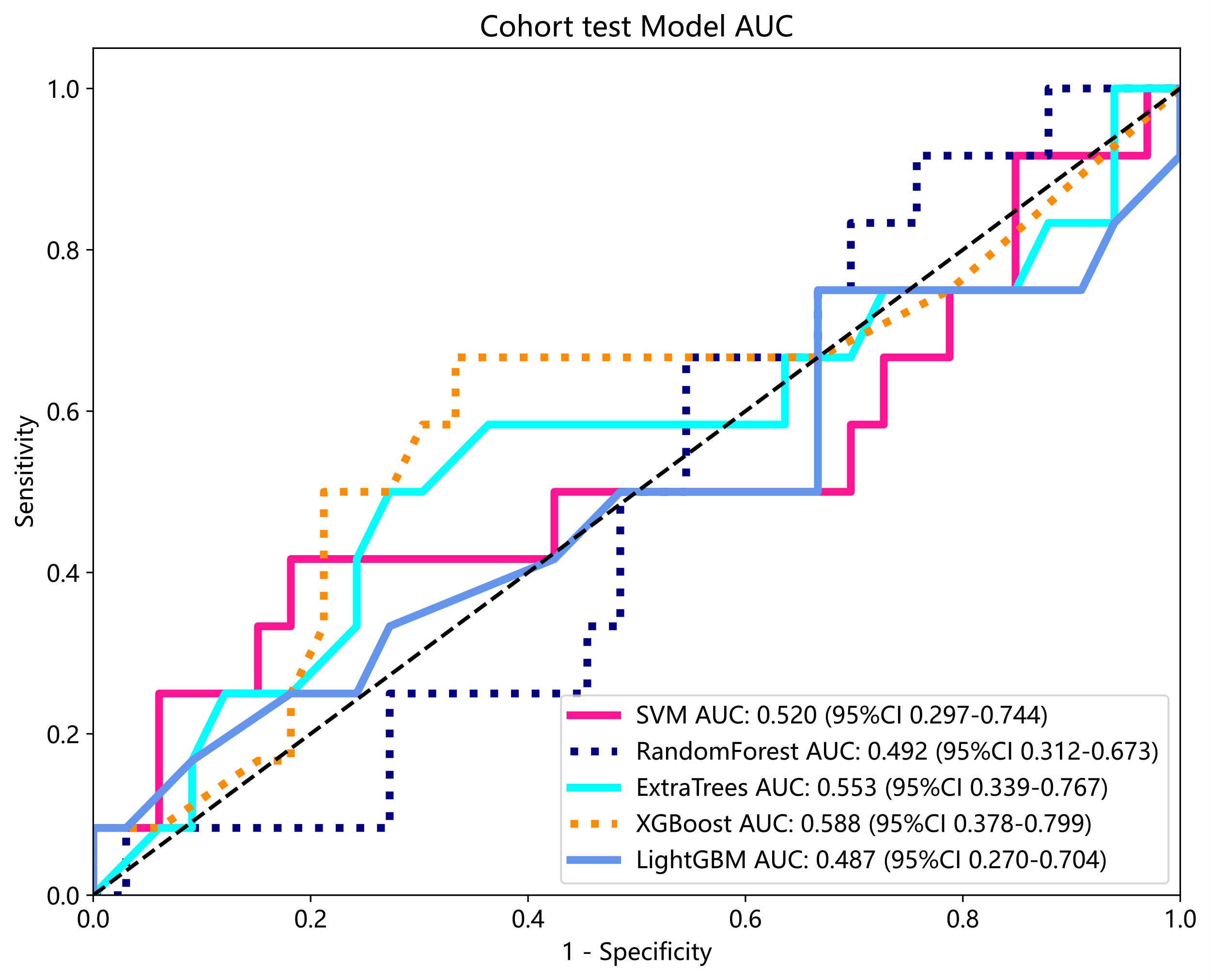


**F**

Figure S2. Coefficients(A), Mean standard error(B) and Weights(C) of 10 fold cross validation of GTV signatures, and ROC curves of machine learning models for GTV in train (D),validation(E), and testing cohort(F)

Table S2. Metrics of GTV in three different tasks

| Model_name | Accuracy | AUC | 95% CI | Sensitivity | Specificity | PPV | NPV | Task |
| --- | --- | --- | --- | --- | --- | --- | --- | --- |
| SVM | 0.900 | 0.918 | 0.8249 - 1.0000 | 0.867 | 0.906 | 0.619 | 0.975 | Train |
| SVM | 0.659 | 0.680 | 0.5154 - 0.8443 | 0.667 | 0.658 | 0.235 | 0.926 | Val |
| SVM | 0.689 | 0.520 | 0.2969 - 0.7435 | 0.333 | 0.818 | 0.400 | 0.771 | Test |
| RandomForest | 0.920 | 0.896 | 0.7813 - 1.0000 | 0.667 | 0.965 | 0.769 | 0.943 | Train |
| RandomForest | 0.500 | 0.702 | 0.5186 - 0.8849 | 0.833 | 0.447 | 0.192 | 0.944 | Val |
| RandomForest | 0.400 | 0.492 | 0.3117 - 0.6732 | 0.833 | 0.242 | 0.286 | 0.800 | Test |
| ExtraTrees | 0.770 | 0.885 | 0.7903 - 0.9799 | 0.800 | 0.765 | 0.375 | 0.956 | Train |
| ExtraTrees | 0.659 | 0.704 | 0.4905 - 0.9174 | 0.500 | 0.684 | 0.200 | 0.897 | Val |
| ExtraTrees | 0.667 | 0.553 | 0.3386 - 0.7675 | 0.417 | 0.758 | 0.385 | 0.781 | Test |
| XGBoost | 0.860 | 0.952 | 0.9131 - 0.9905 | 0.933 | 0.847 | 0.519 | 0.986 | Train |
| XGBoost | 0.545 | 0.564 | 0.3018 - 0.8254 | 0.500 | 0.553 | 0.150 | 0.875 | Val |
| XGBoost | 0.644 | 0.588 | 0.3775 - 0.7992 | 0.583 | 0.667 | 0.389 | 0.815 | Test |
| LightGBM | 0.770 | 0.878 | 0.7959 - 0.9602 | 0.733 | 0.776 | 0.367 | 0.943 | Train |
| LightGBM | 0.727 | 0.564 | 0.2880 - 0.8392 | 0.333 | 0.789 | 0.200 | 0.882 | Val |
| LightGBM | 0.422 | 0.487 | 0.2703 - 0.7044 | 0.667 | 0.333 | 0.267 | 0.733 | Test |

*Abbreviation: GTV:gross target volume;AUC:areas under the curve;CI:confidence interval;PPV:positive predictive value; NPV:negative predictive value;SVM:support vector machine.*


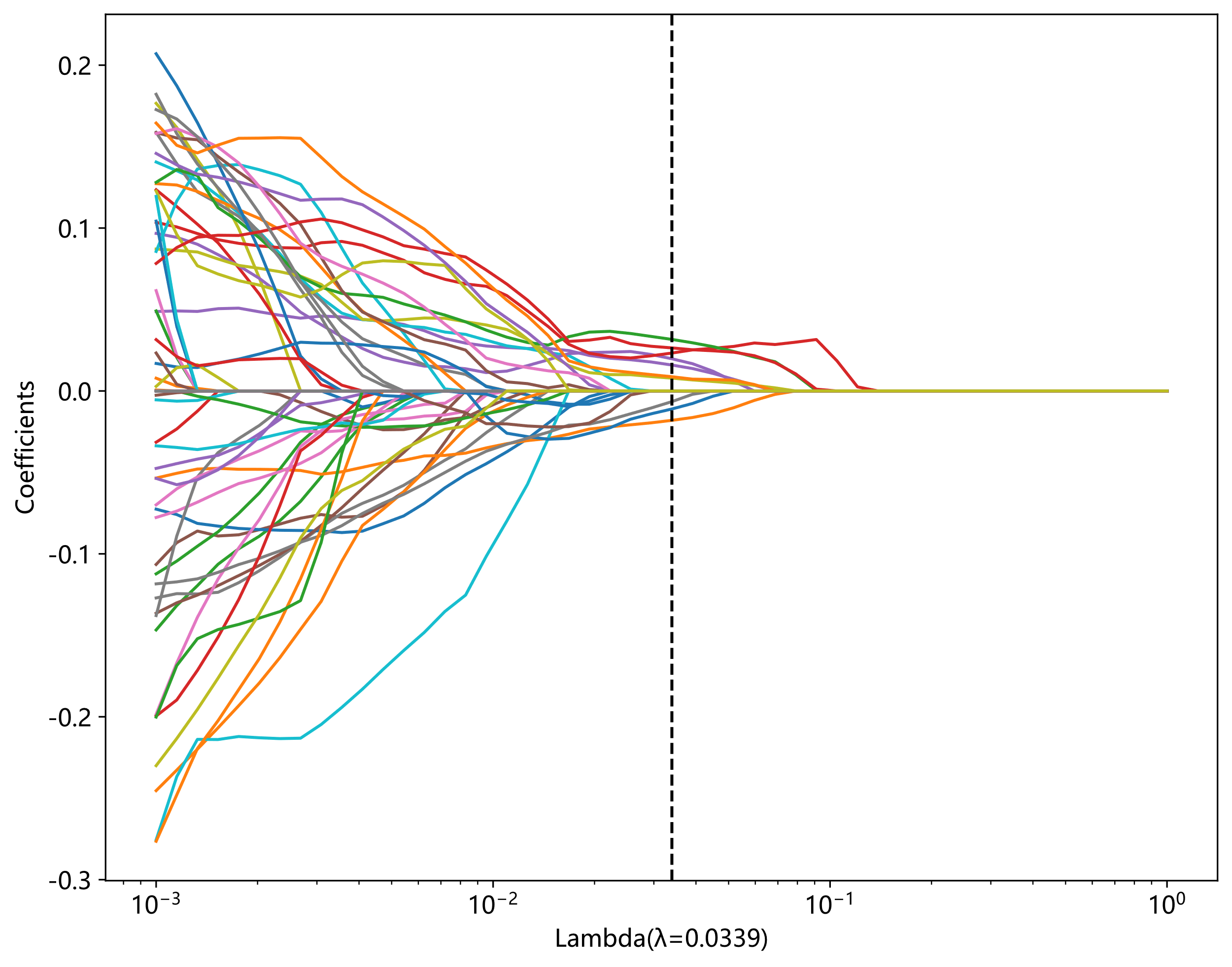


**A**


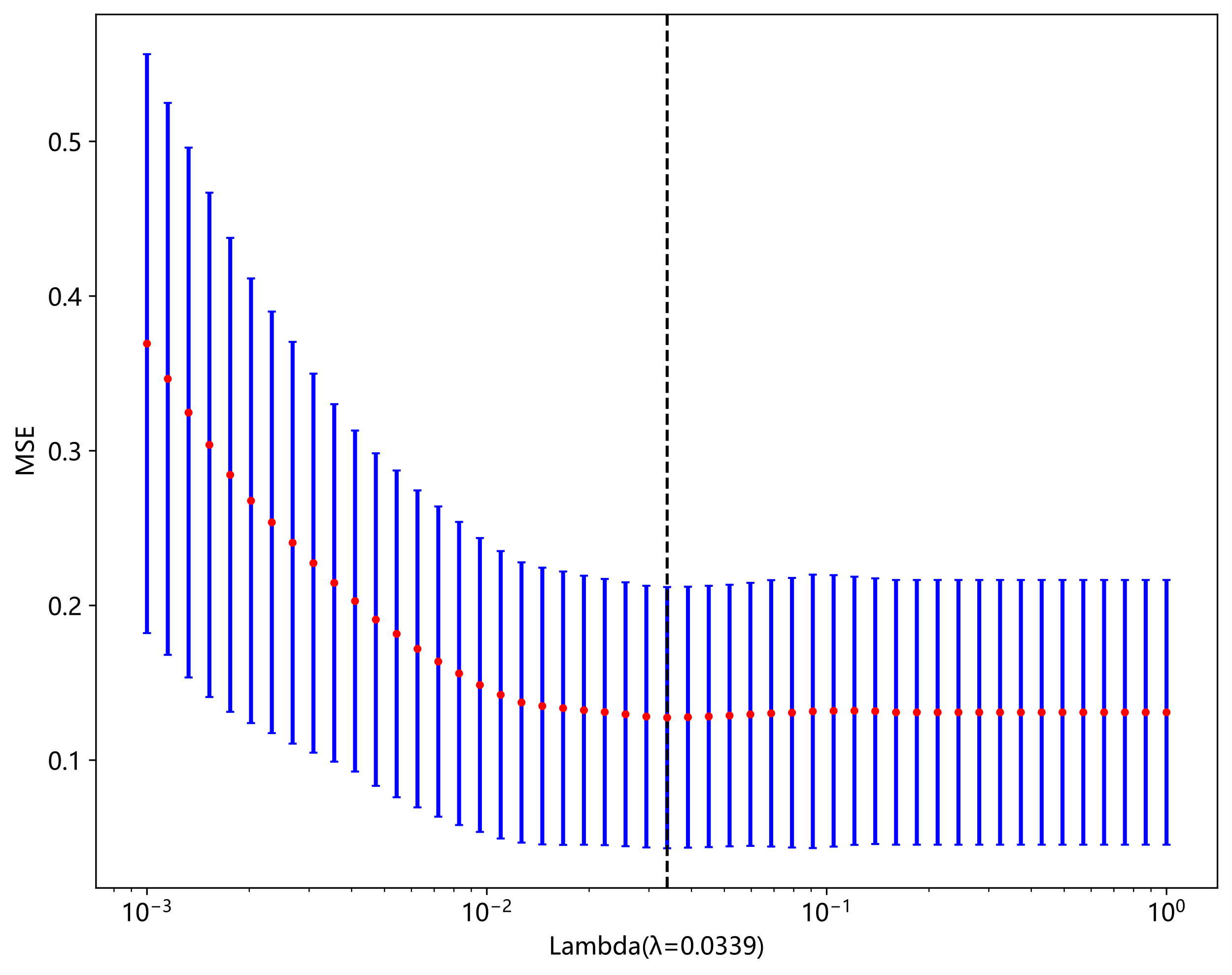


**B**


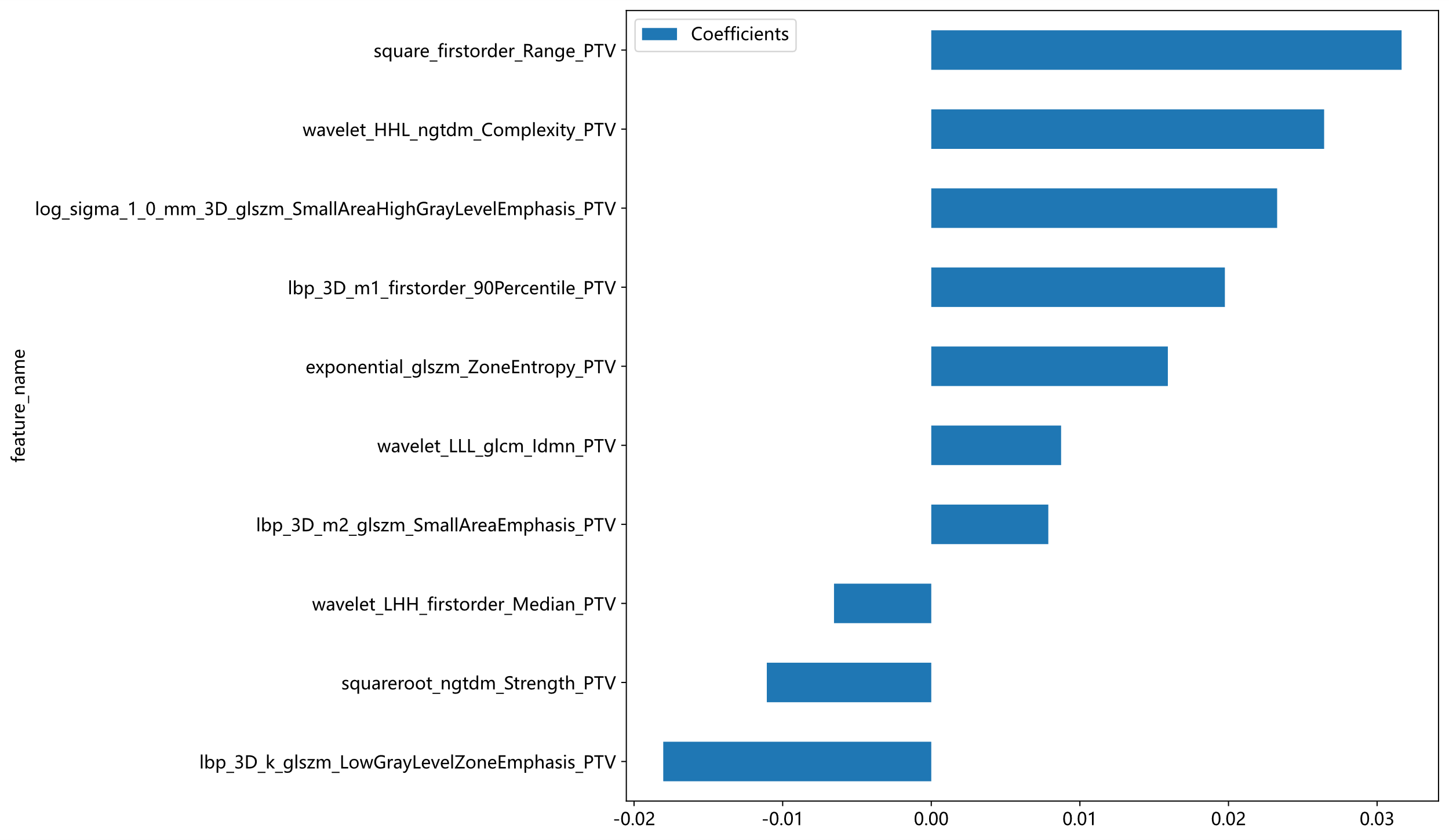


**C**


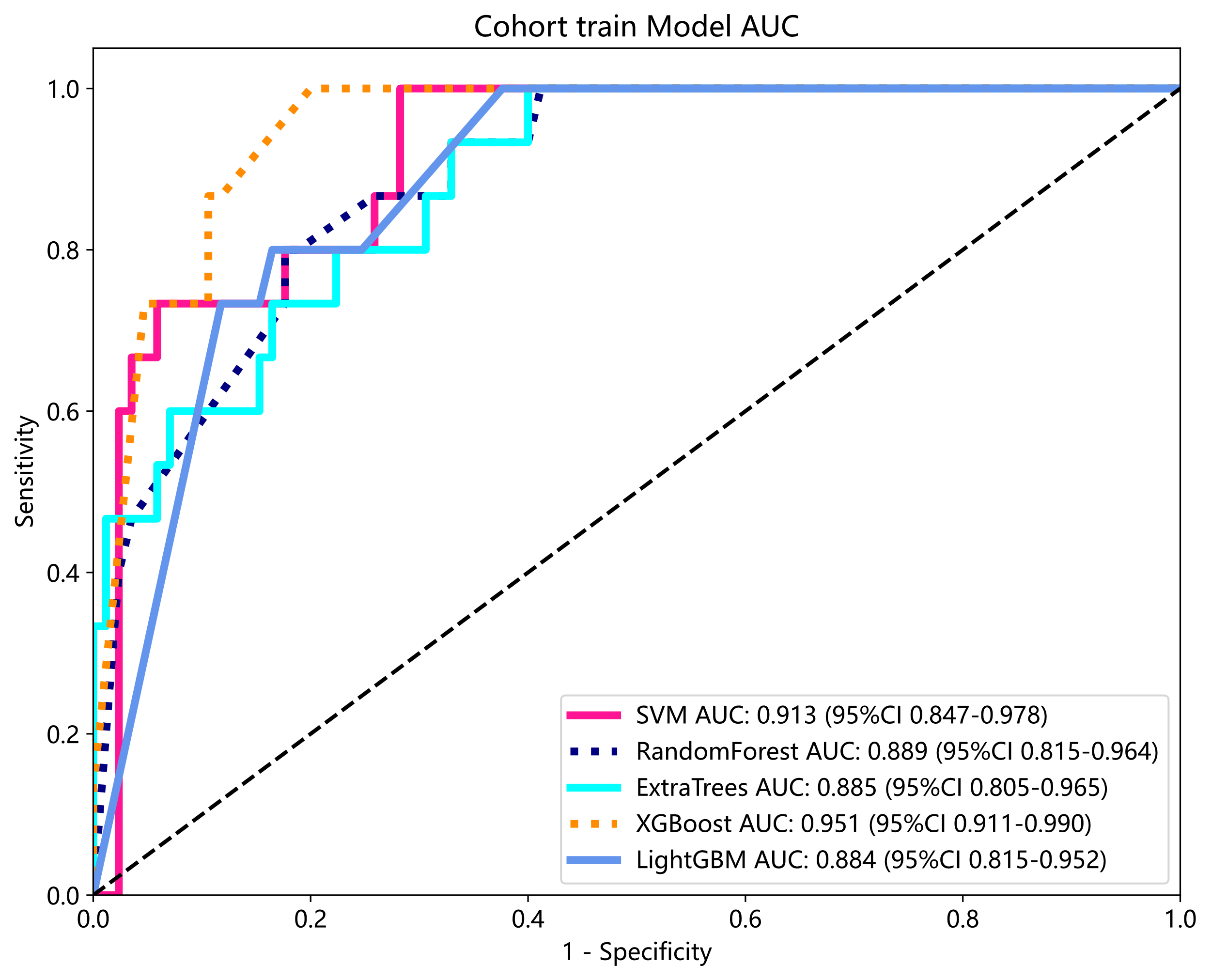


**D**


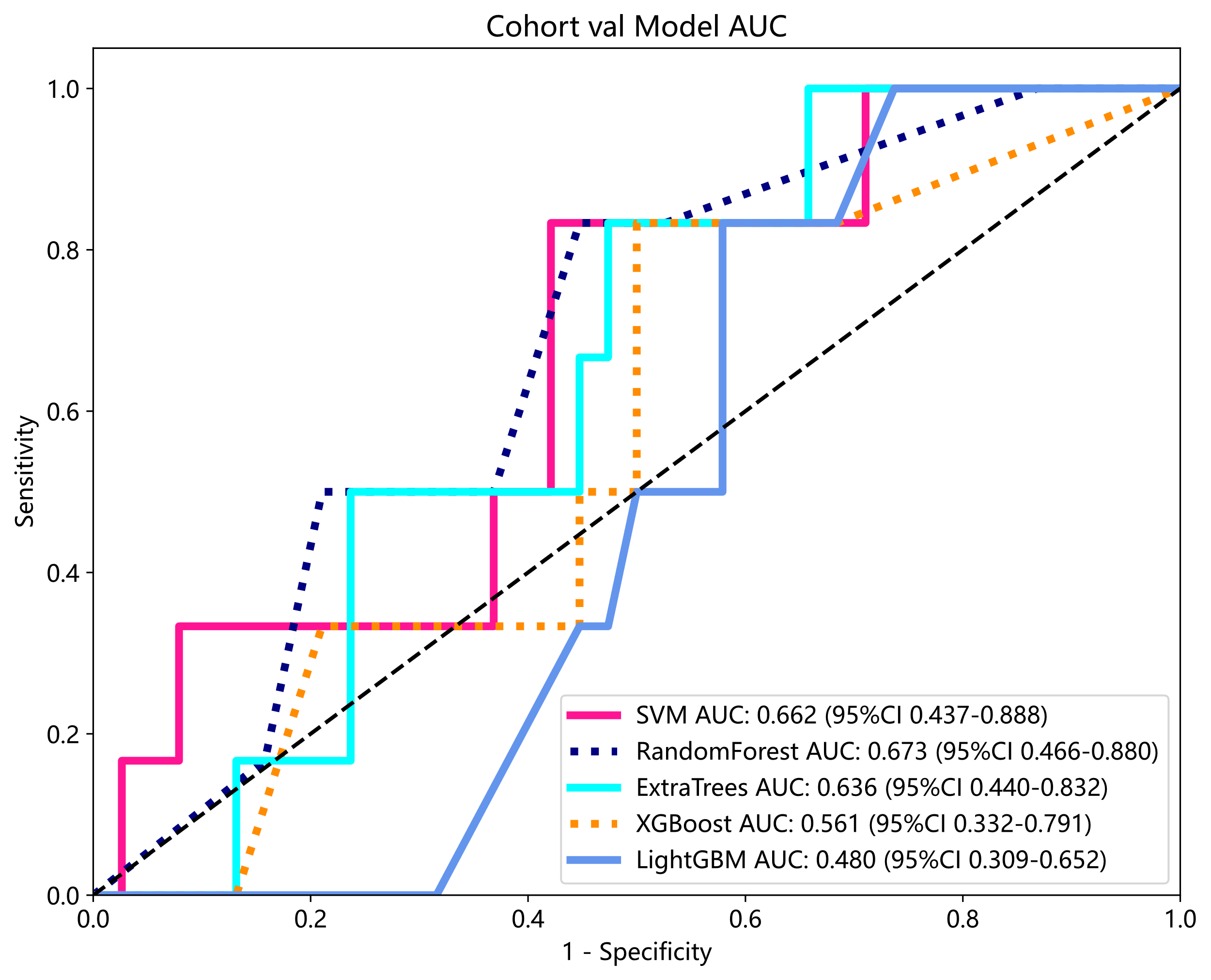


**E**


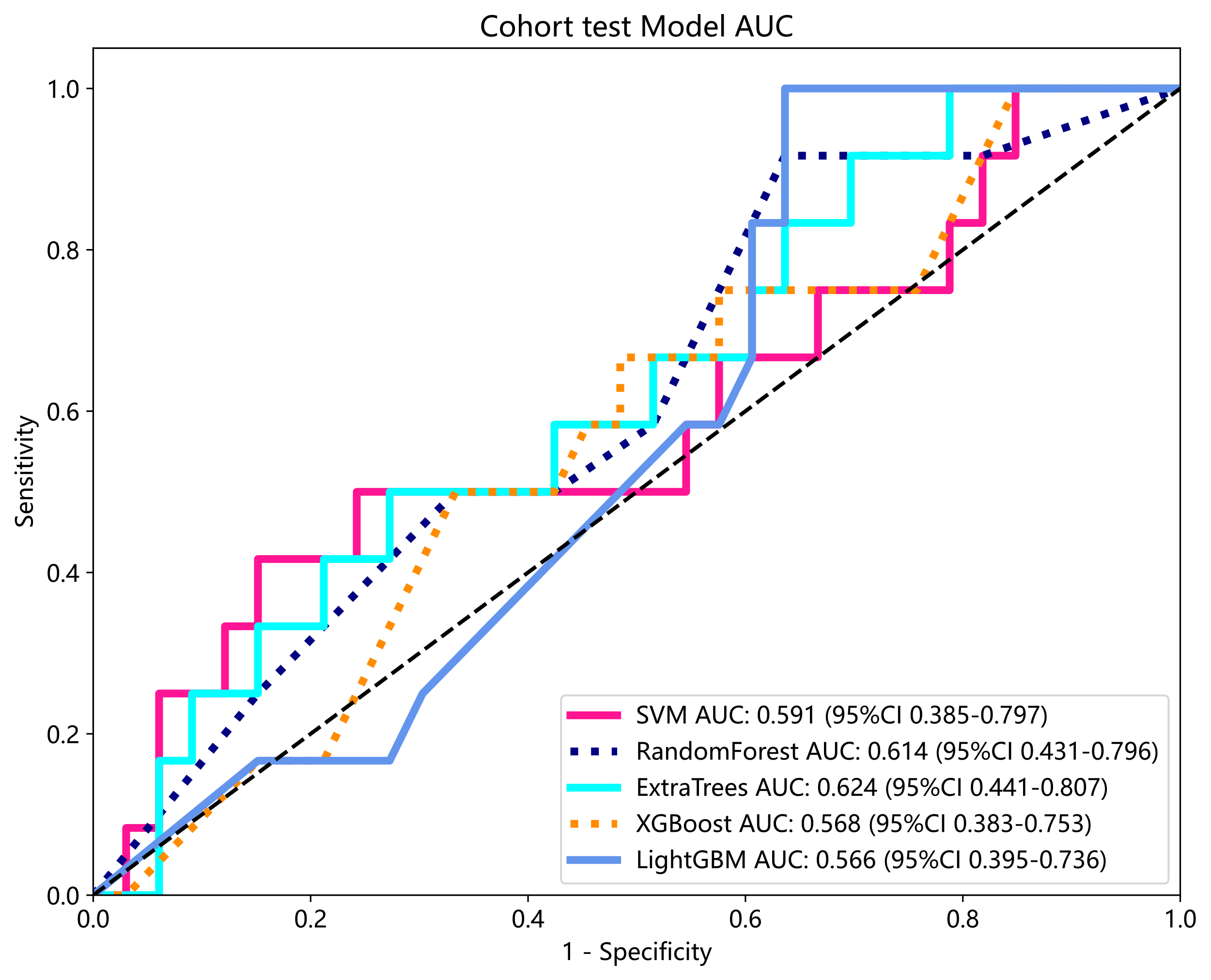


**F**

Figure S3. Coefficients(A), Mean standard error(B) and Weights(C) of 10 fold cross validation of PTV signatures, and ROC curves of machine learning models for PTV in train (D),validation(E), and testing cohort(F)

Table S3. Metrics of PTV in three different tasks

| Model_name | Accuracy | AUC | 95% CI | Sensitivity | Specificity | PPV | NPV | Task |
| --- | --- | --- | --- | --- | --- | --- | --- | --- |
| SVM | 0.750 | 0.913 | 0.8474 - 0.9785 | 0.933 | 0.718 | 0.368 | 0.984 | Train |
| SVM | 0.591 | 0.662 | 0.4366 - 0.8880 | 0.667 | 0.579 | 0.200 | 0.917 | Val |
| SVM | 0.711 | 0.591 | 0.3851 - 0.7967 | 0.333 | 0.848 | 0.444 | 0.778 | Test |
| RandomForest | 0.810 | 0.889 | 0.8153 - 0.9636 | 0.733 | 0.824 | 0.423 | 0.946 | Train |
| RandomForest | 0.614 | 0.673 | 0.4660 - 0.8805 | 0.500 | 0.632 | 0.176 | 0.889 | Val |
| RandomForest | 0.511 | 0.614 | 0.4310 - 0.7963 | 0.667 | 0.455 | 0.308 | 0.789 | Test |
| ExtraTrees | 0.700 | 0.885 | 0.8049 - 0.9645 | 0.867 | 0.671 | 0.317 | 0.966 | Train |
| ExtraTrees | 0.545 | 0.636 | 0.4402 - 0.8318 | 0.667 | 0.526 | 0.182 | 0.909 | Val |
| ExtraTrees | 0.644 | 0.624 | 0.4405 - 0.8069 | 0.417 | 0.727 | 0.357 | 0.774 | Test |
| XGBoost | 0.880 | 0.951 | 0.9112 - 0.9900 | 0.867 | 0.882 | 0.565 | 0.974 | Train |
| XGBoost | 0.500 | 0.561 | 0.3321 - 0.7907 | 0.500 | 0.500 | 0.136 | 0.864 | Val |
| XGBoost | 0.533 | 0.568 | 0.3830 - 0.7534 | 0.583 | 0.515 | 0.304 | 0.773 | Test |
| LightGBM | 0.830 | 0.884 | 0.8154 - 0.9524 | 0.733 | 0.847 | 0.458 | 0.947 | Train |
| LightGBM | 0.386 | 0.480 | 0.3090 - 0.6516 | 0.833 | 0.316 | 0.161 | 0.923 | Val |
| LightGBM | 0.489 | 0.566 | 0.3951 - 0.7362 | 0.833 | 0.364 | 0.323 | 0.857 | Test |

*Abbreviation: PTV:planning target volume;AUC:areas under the curve;CI:confidence interval;PPV:positive predictive value; NPV:negative predictive value;SVM:support vector machine.*


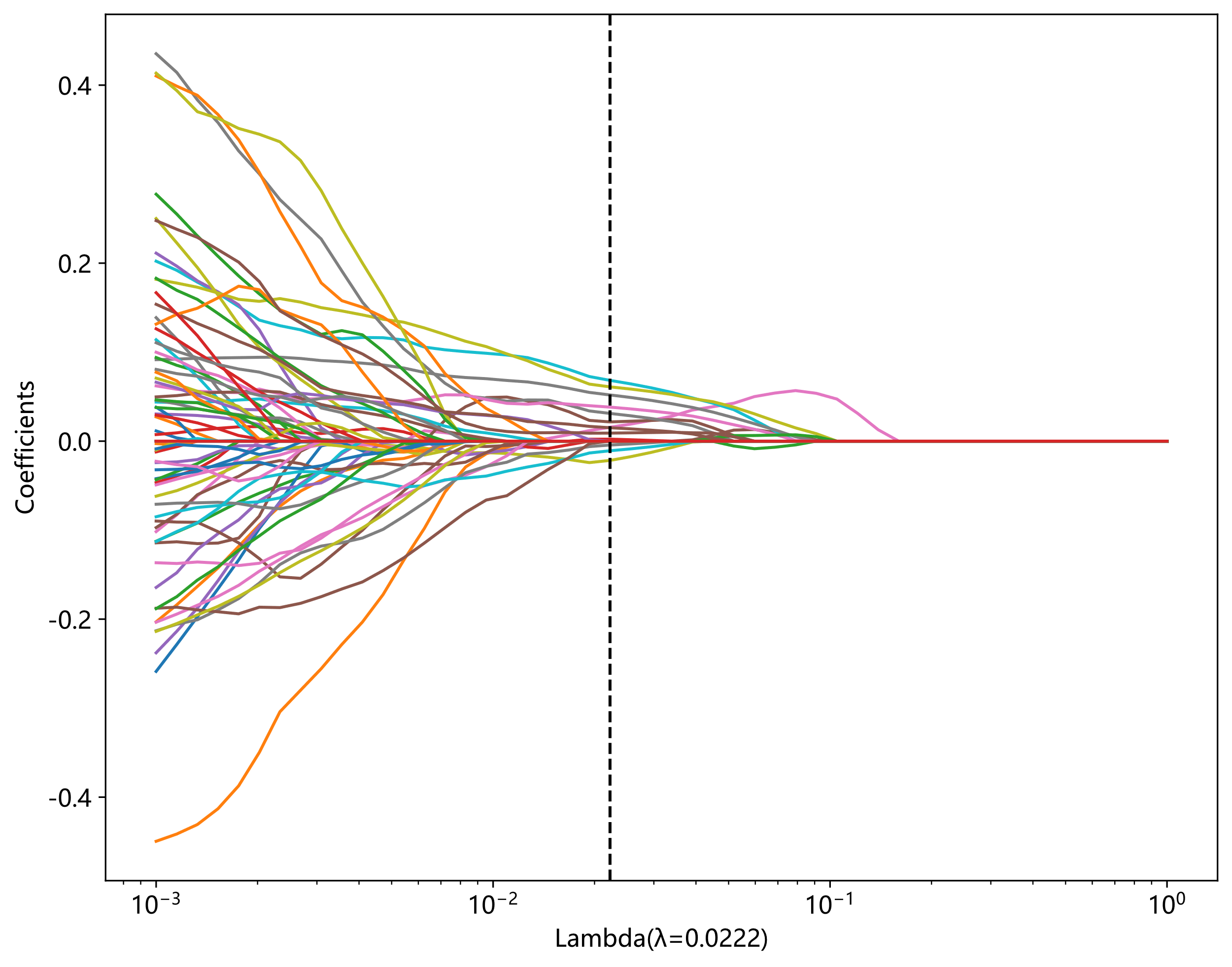


**A**


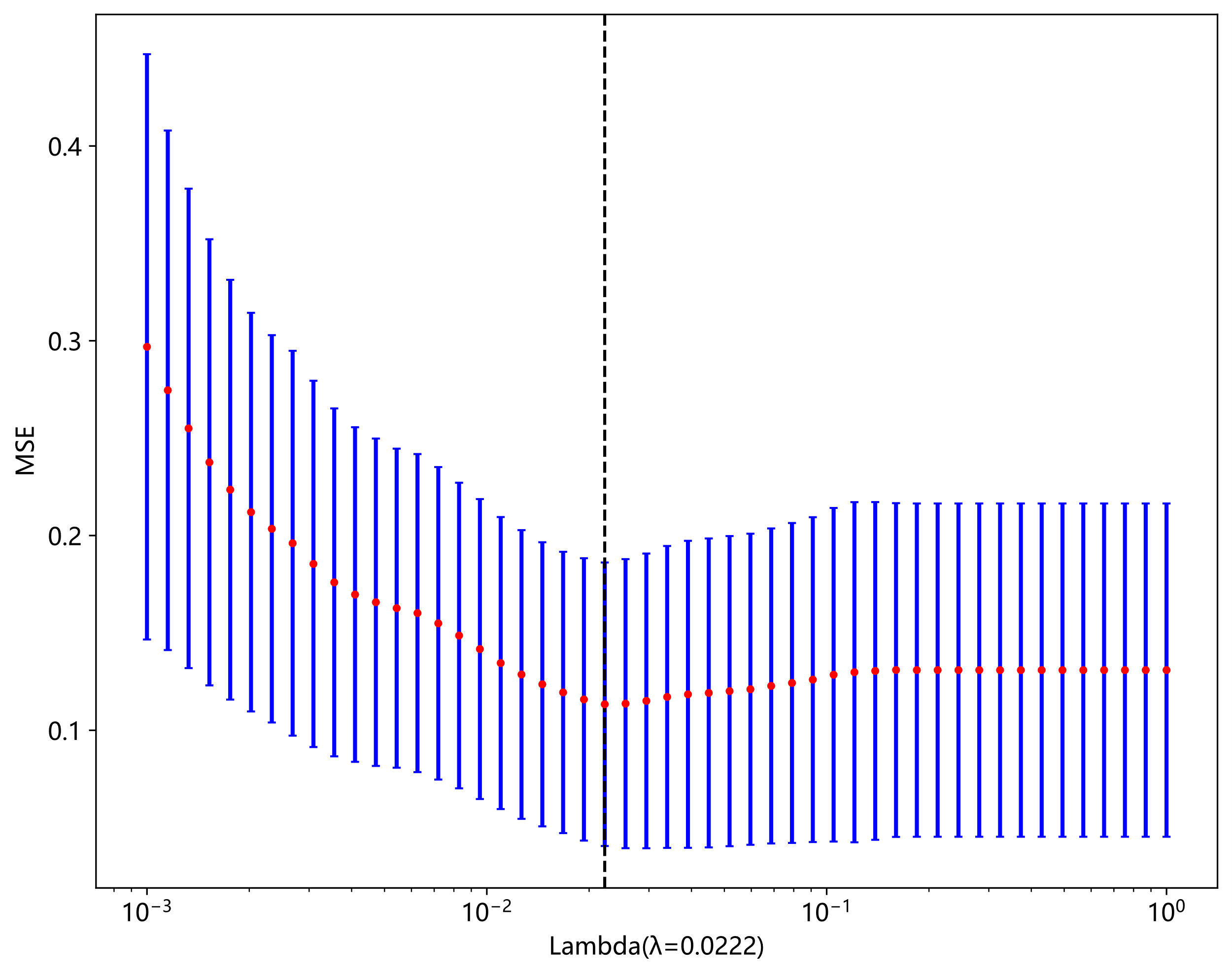


**B**


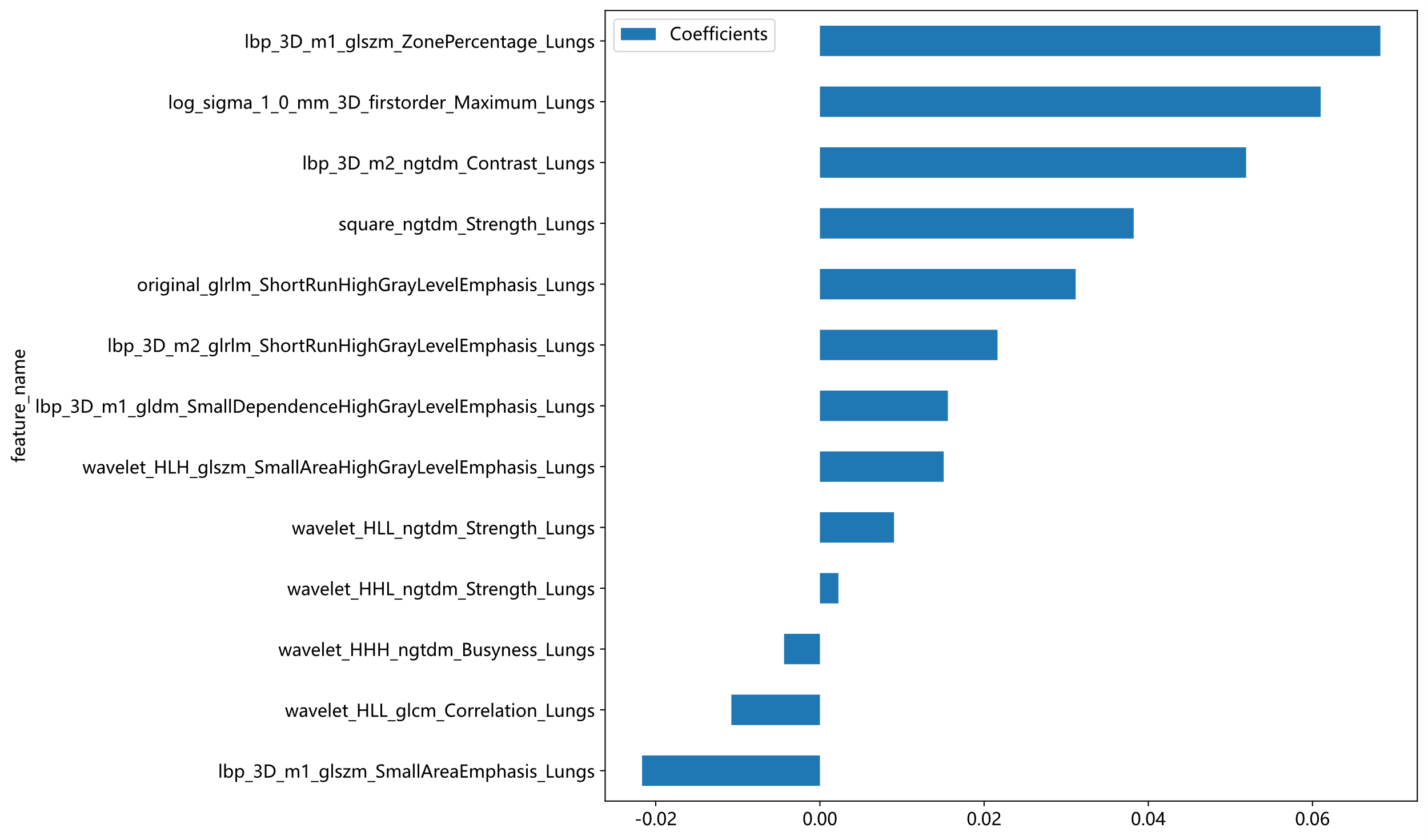


**C**


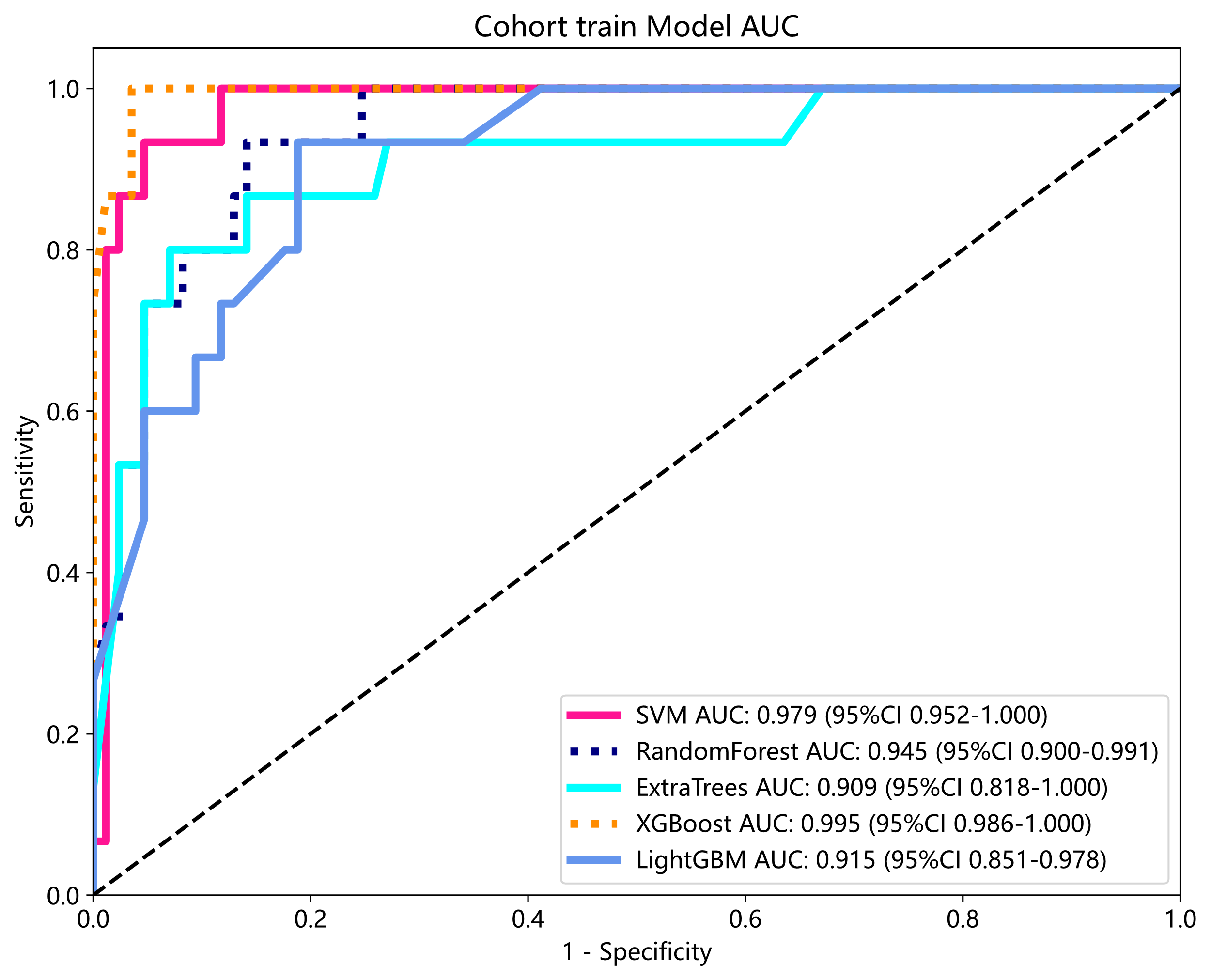


**D**


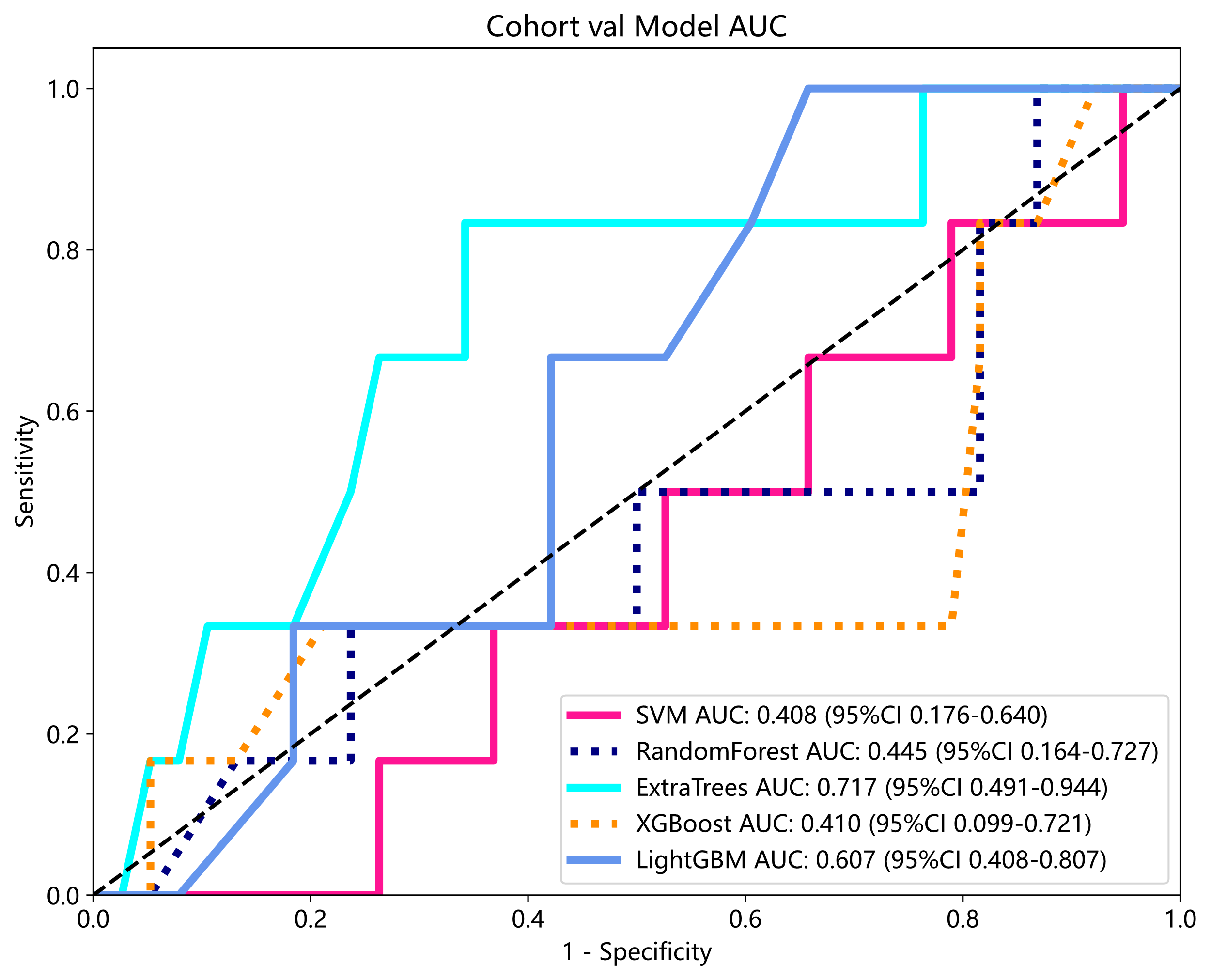


**E**


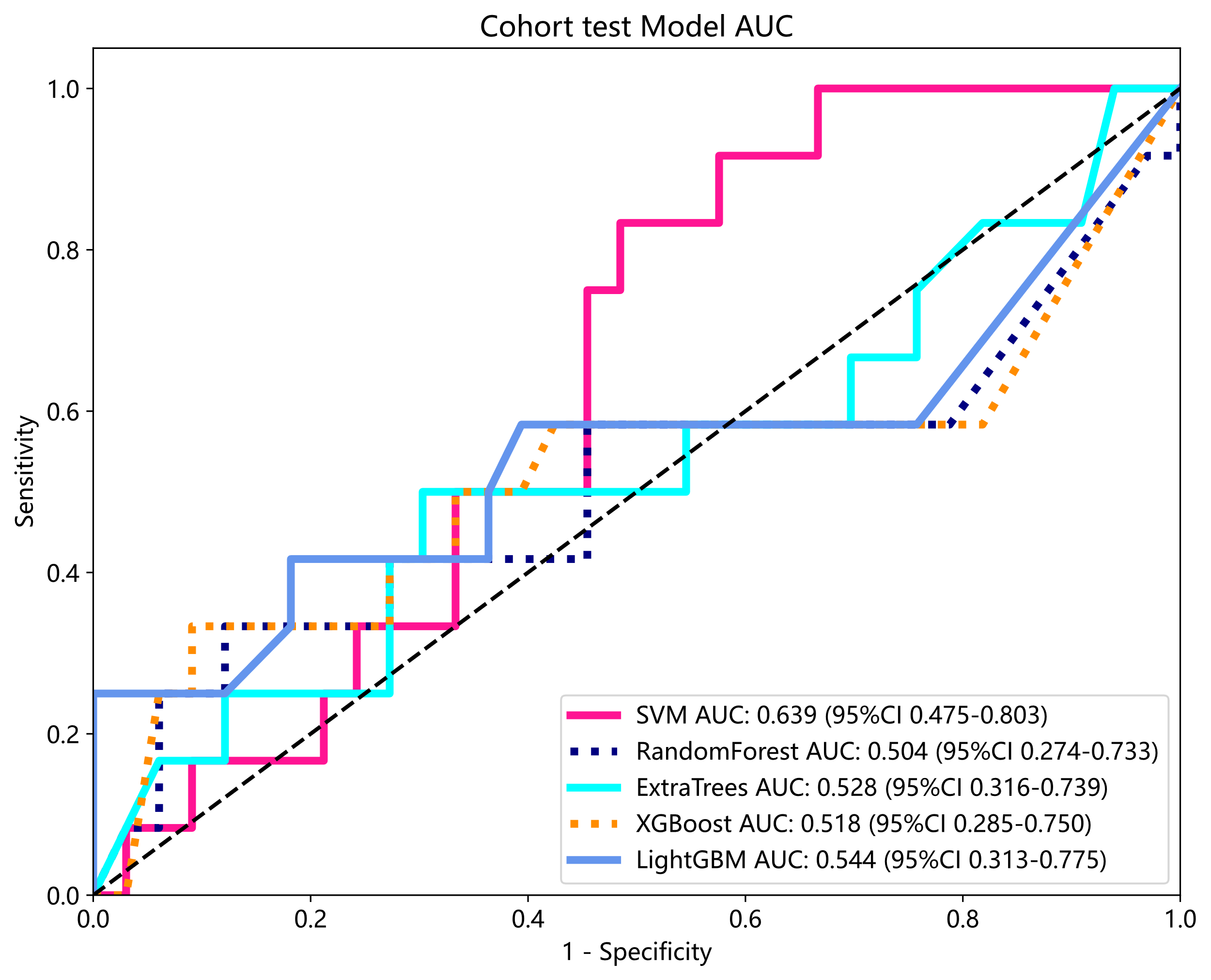


**F**

Figure S4. Coefficients(A), Mean standard error(B) and Weights(C) of 10 fold cross validation of Lungs signatures, and ROC curves of machine learning models for Lungs in train (D),validation(E), and testing cohort(F)

Table S4. Metrics of Lungs in three different tasks

| Model_name | Accuracy | AUC | 95% CI | Sensitivity | Specificity | PPV | NPV | Task |
| --- | --- | --- | --- | --- | --- | --- | --- | --- |
| SVM | 0.940 | 0.979 | 0.9521 - 1.0000 | 0.867 | 0.953 | 0.765 | 0.976 | Train |
| SVM | 0.159 | 0.408 | 0.1761 - 0.6396 | 0.833 | 0.053 | 0.122 | 0.667 | Val |
| SVM | 0.578 | 0.639 | 0.4745 - 0.8033 | 0.750 | 0.515 | 0.360 | 0.850 | Test |
| RandomForest | 0.860 | 0.945 | 0.8999 - 0.9911 | 0.867 | 0.859 | 0.520 | 0.973 | Train |
| RandomForest | 0.227 | 0.445 | 0.1637 - 0.7267 | 0.833 | 0.132 | 0.132 | 0.833 | Val |
| RandomForest | 0.711 | 0.504 | 0.2744 - 0.7332 | 0.250 | 0.879 | 0.429 | 0.763 | Test |
| ExtraTrees | 0.900 | 0.909 | 0.8182 - 0.9998 | 0.733 | 0.929 | 0.647 | 0.952 | Train |
| ExtraTrees | 0.659 | 0.717 | 0.4906 - 0.9436 | 0.667 | 0.658 | 0.235 | 0.926 | Val |
| ExtraTrees | 0.622 | 0.528 | 0.3165 - 0.7391 | 0.417 | 0.697 | 0.333 | 0.767 | Test |
| XGBoost | 0.960 | 0.995 | 0.9857 - 1.0000 | 0.933 | 0.965 | 0.824 | 0.988 | Train |
| XGBoost | 0.773 | 0.410 | 0.0992 - 0.7210 | 0.167 | 0.868 | 0.167 | 0.868 | Val |
| XGBoost | 0.733 | 0.518 | 0.2849 - 0.7504 | 0.250 | 0.909 | 0.500 | 0.769 | Test |
| LightGBM | 0.820 | 0.915 | 0.8509 - 0.9781 | 0.867 | 0.812 | 0.448 | 0.972 | Train |
| LightGBM | 0.455 | 0.607 | 0.4082 - 0.8068 | 0.833 | 0.395 | 0.179 | 0.937 | Val |
| LightGBM | 0.733 | 0.544 | 0.3131 - 0.7753 | 0.000 | 1.000 | 0.000 | 0.733 | Test |

*Abbreviation: AUC:areas under the curve;CI:confidence interval;PPV:positive predictive value; NPV:negative predictive value;SVM:support vector machine.*


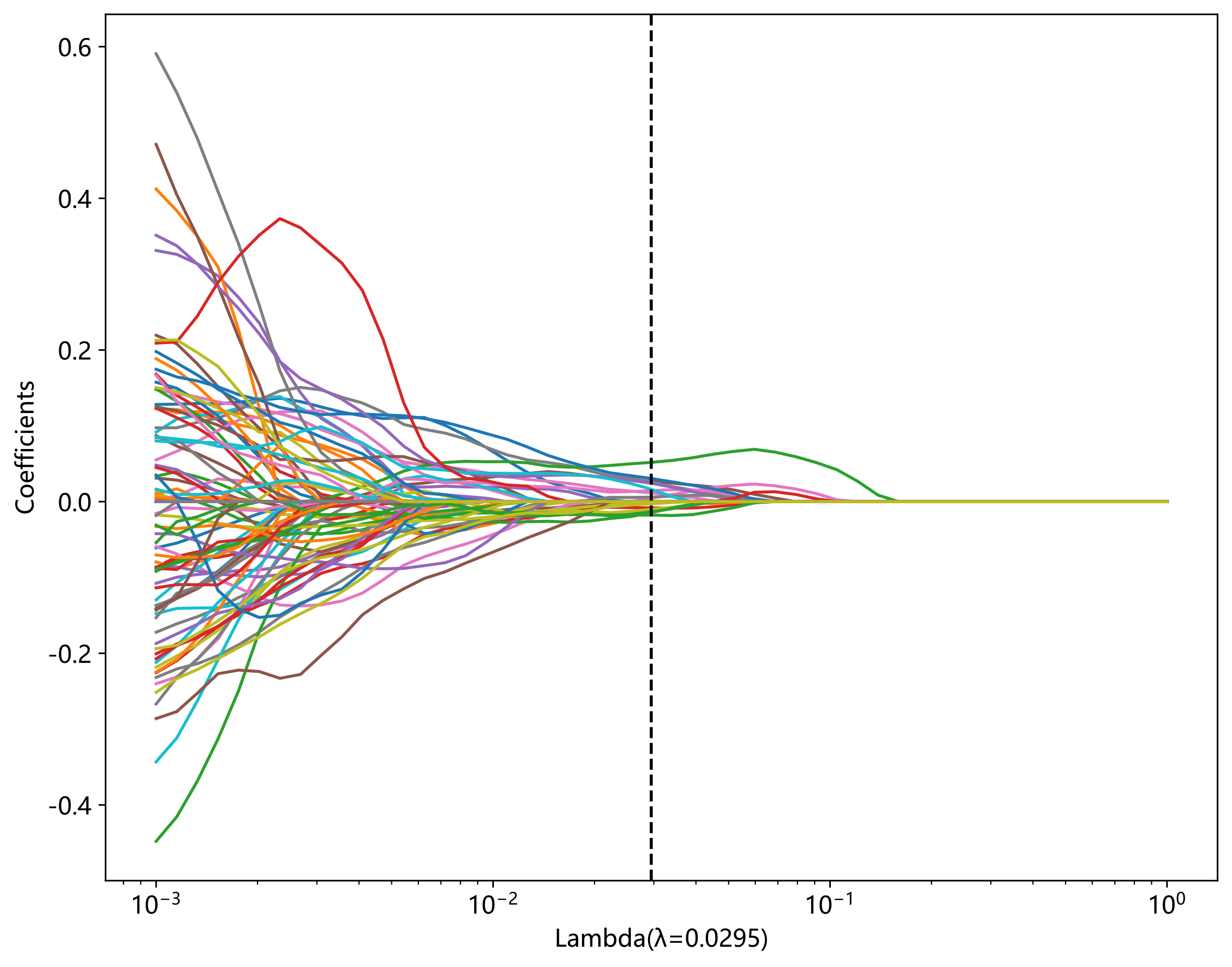


**A**


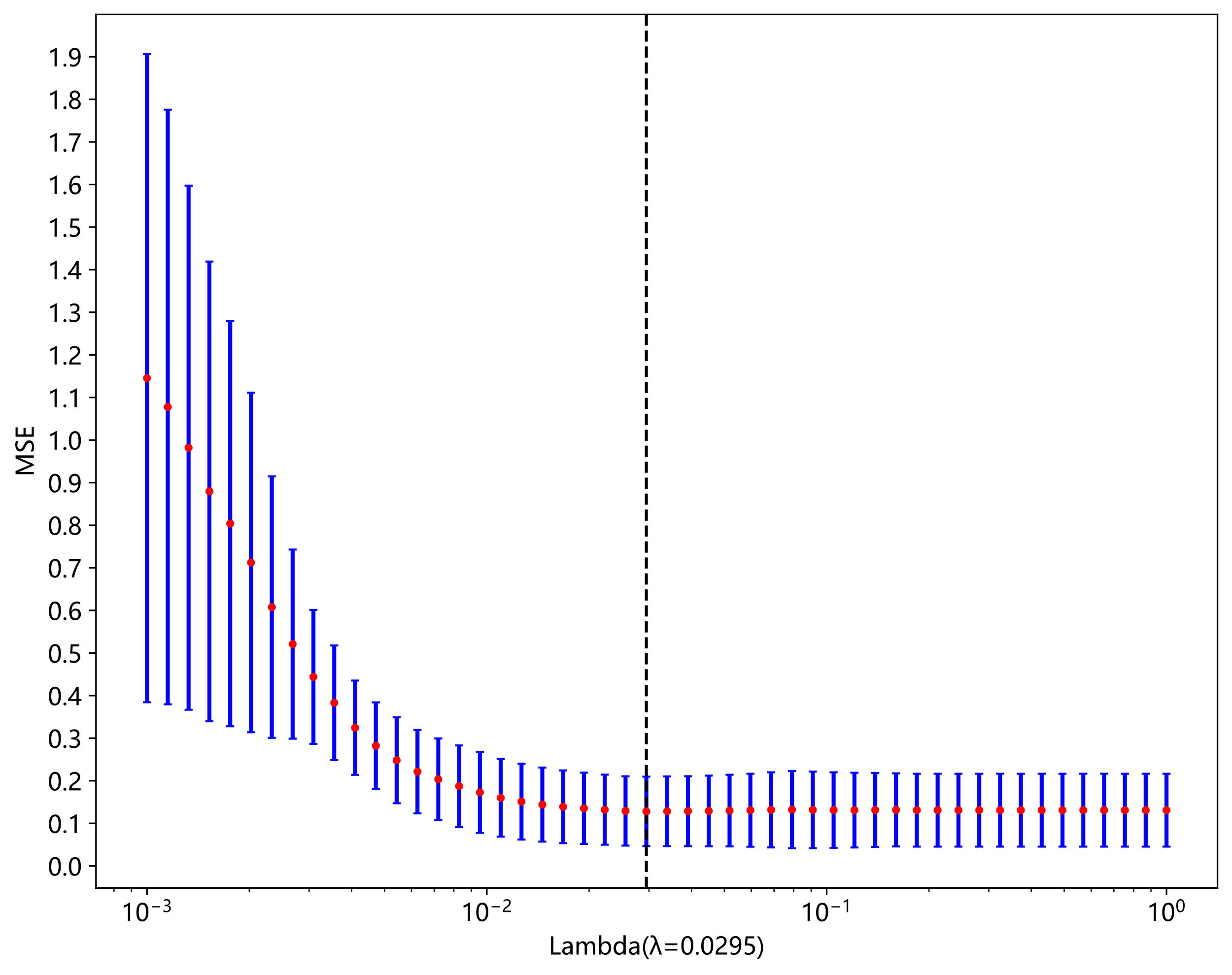


**B**


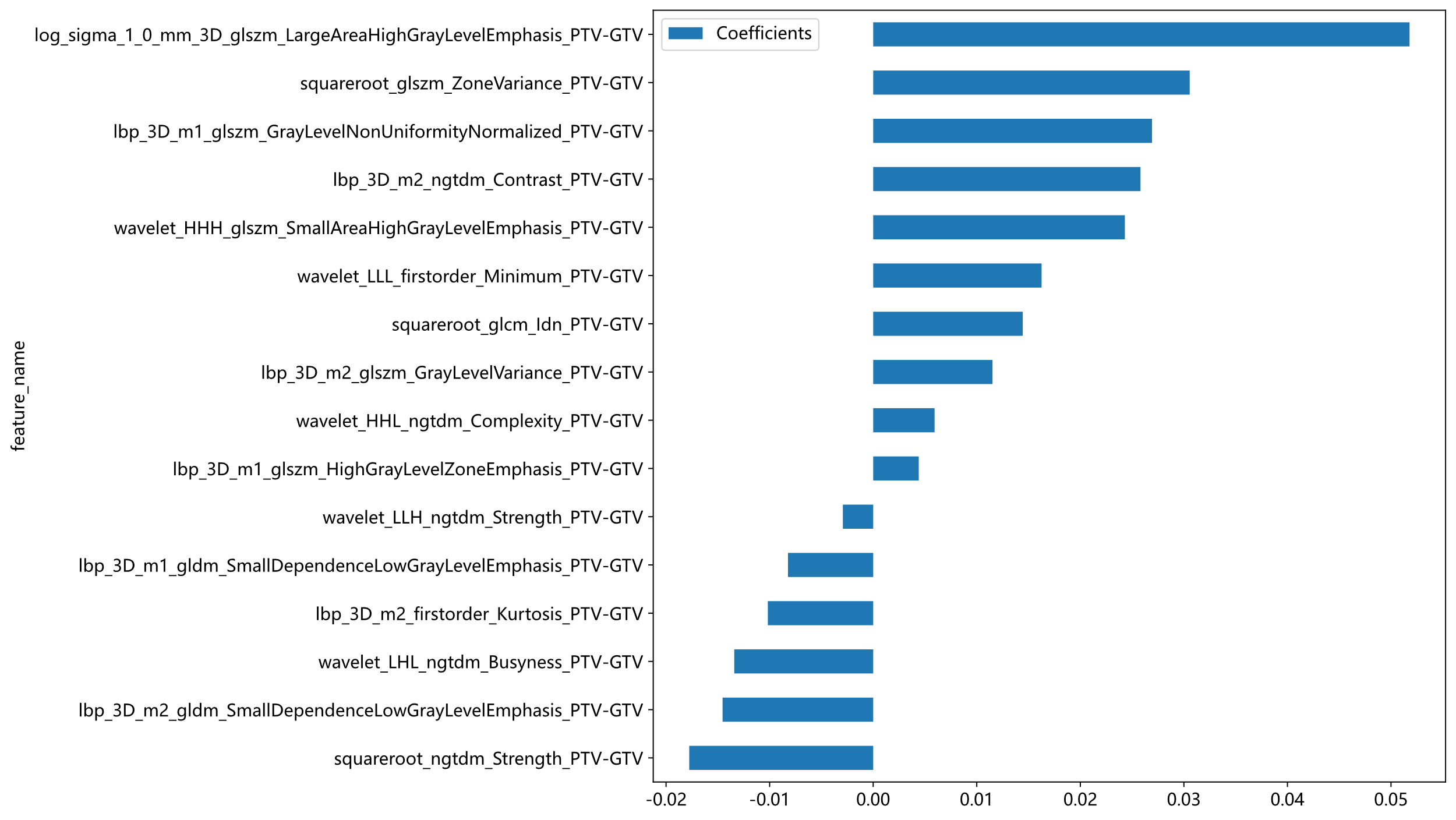


**C**


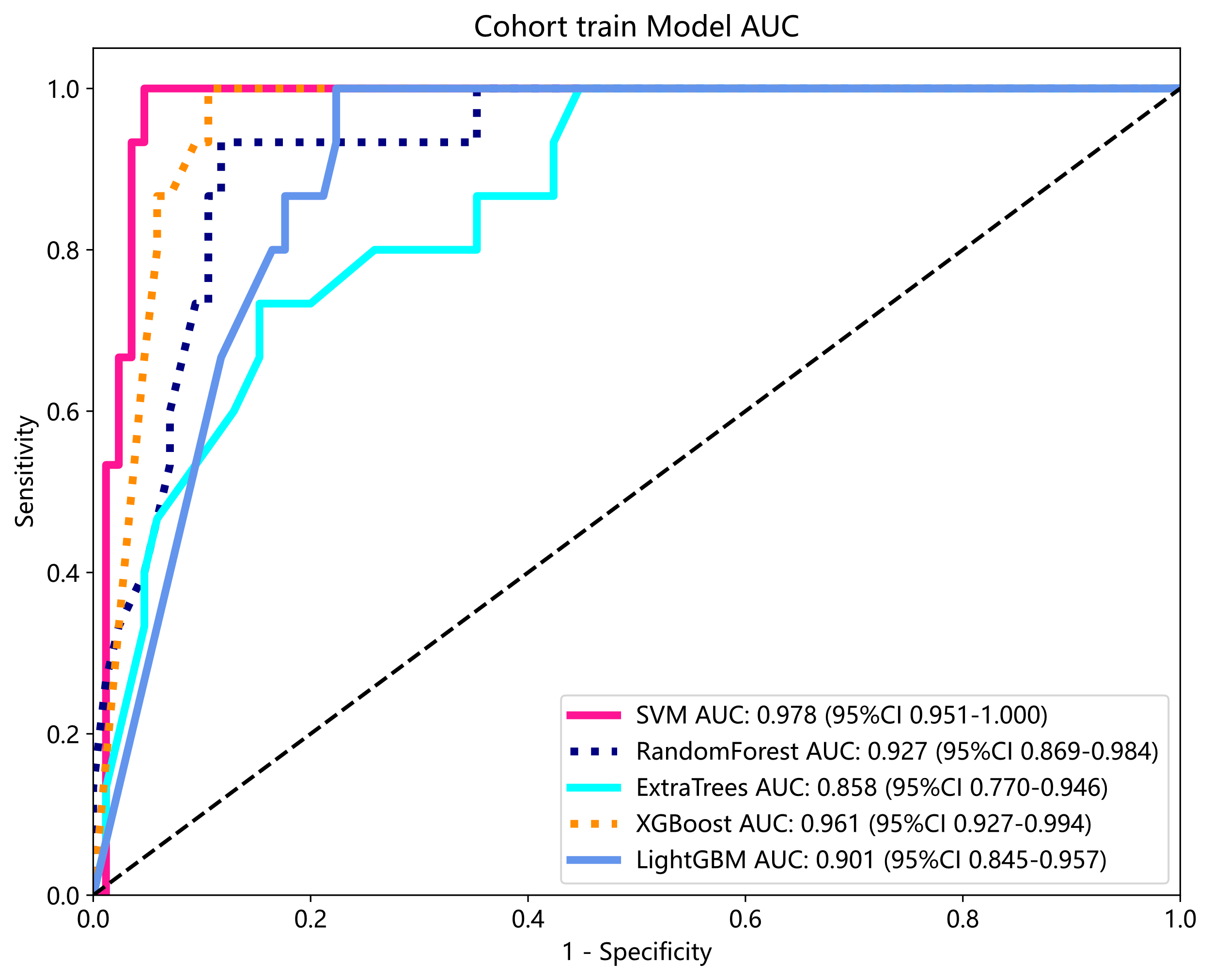


**D**


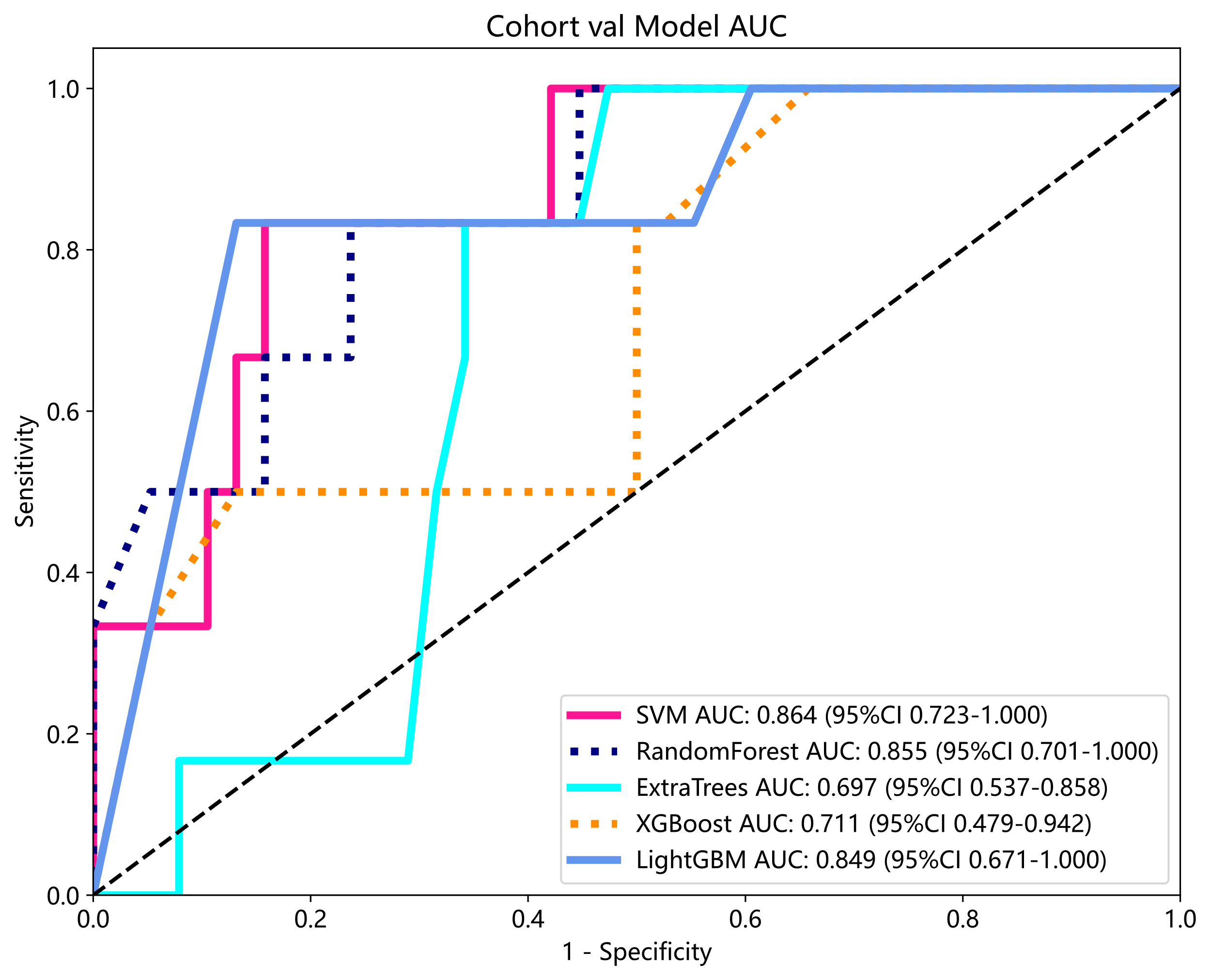


**E**


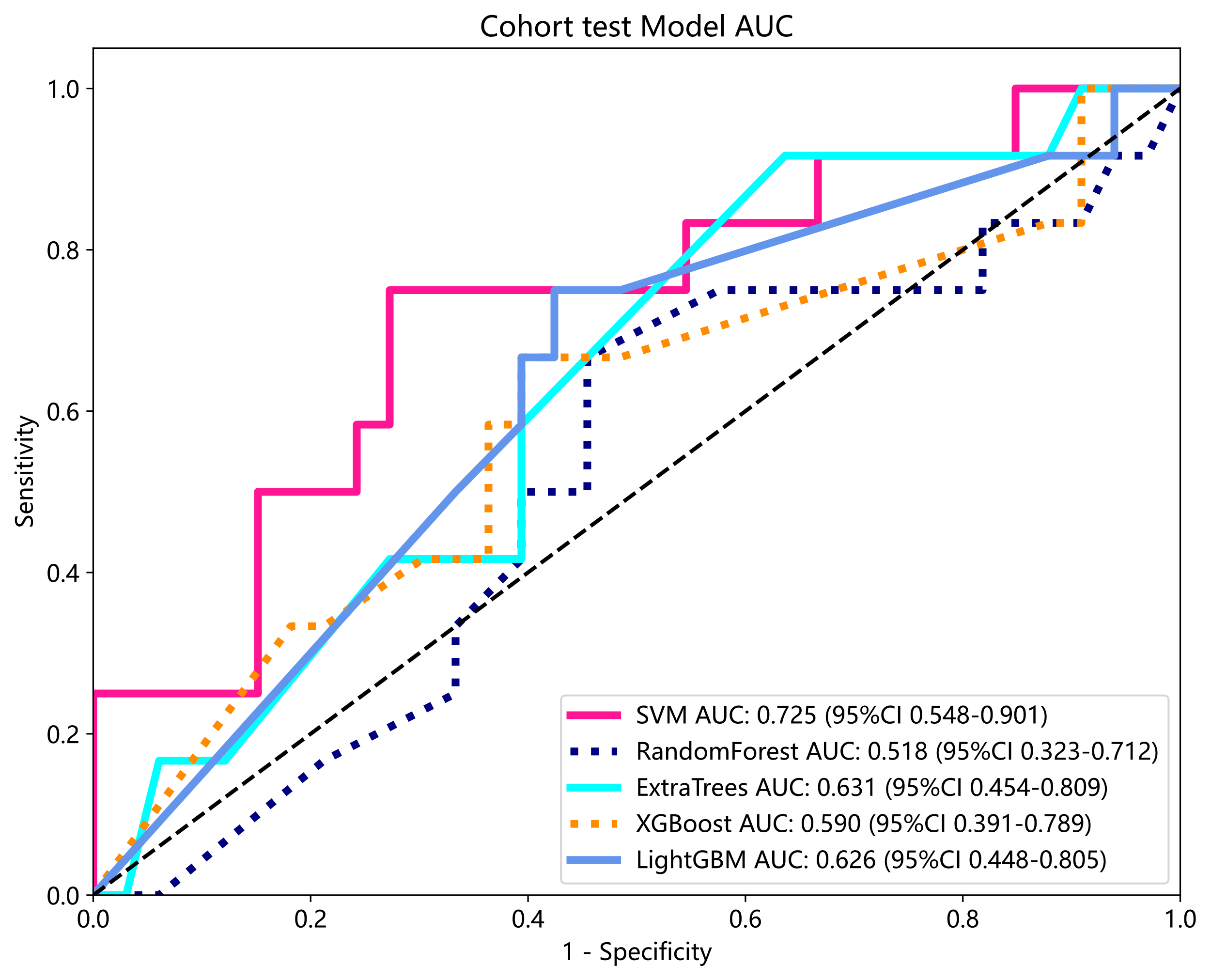


**F**

Figure S5. Coefficients(A), Mean standard error(B) and Weights(C) of 10 fold cross validation of PTV-GTV signatures, and ROC curves of machine learning models for PTV-GTV in train (D),validation(E), and testing cohort(F)

Table S5. Metrics of PTV-GTV in three different tasks

| Model_name | Accuracy | AUC | 95% CI | Sensitivity | Specificity | PPV | NPV | Task |
| --- | --- | --- | --- | --- | --- | --- | --- | --- |
| SVM | 0.950 | 0.978 | 0.9509 - 1.0000 | 0.933 | 0.953 | 0.778 | 0.988 | Train |
| SVM | 0.818 | 0.864 | 0.7226 - 1.0000 | 0.667 | 0.842 | 0.400 | 0.941 | Val |
| SVM | 0.711 | 0.725 | 0.5480 - 0.9015 | 0.667 | 0.727 | 0.471 | 0.857 | Test |
| RandomForest | 0.880 | 0.927 | 0.8694 - 0.9839 | 0.867 | 0.882 | 0.565 | 0.974 | Train |
| RandomForest | 0.750 | 0.855 | 0.7006 - 1.0000 | 0.667 | 0.763 | 0.308 | 0.935 | Val |
| RandomForest | 0.556 | 0.518 | 0.3233 - 0.7120 | 0.583 | 0.545 | 0.318 | 0.783 | Test |
| ExtraTrees | 0.820 | 0.858 | 0.7697 - 0.9463 | 0.667 | 0.847 | 0.435 | 0.935 | Train |
| ExtraTrees | 0.591 | 0.697 | 0.5369 - 0.8579 | 0.833 | 0.553 | 0.227 | 0.955 | Val |
| ExtraTrees | 0.533 | 0.631 | 0.4539 - 0.8087 | 0.833 | 0.424 | 0.345 | 0.875 | Test |
| XGBoost | 0.900 | 0.961 | 0.9273 - 0.9943 | 0.933 | 0.894 | 0.609 | 0.987 | Train |
| XGBoost | 0.864 | 0.711 | 0.4794 - 0.9417 | 0.333 | 0.947 | 0.500 | 0.900 | Val |
| XGBoost | 0.600 | 0.590 | 0.3905 - 0.7888 | 0.583 | 0.606 | 0.350 | 0.800 | Test |
| LightGBM | 0.800 | 0.901 | 0.8446 - 0.9570 | 0.933 | 0.776 | 0.424 | 0.985 | Train |
| LightGBM | 0.864 | 0.849 | 0.6709 - 1.0000 | 0.000 | 1.000 | 0.000 | 0.864 | Val |
| LightGBM | 0.600 | 0.626 | 0.4476 - 0.8049 | 0.667 | 0.576 | 0.364 | 0.826 | Test |

*Abbreviation: AUC:areas under the curve;CI:confidence interval;PPV:positive predictive value; NPV:negative predictive value;SVM:support vector machine.*


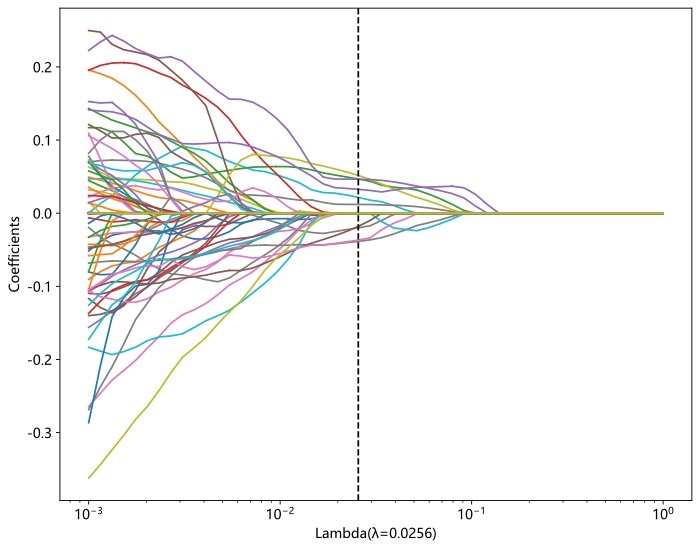


**A**


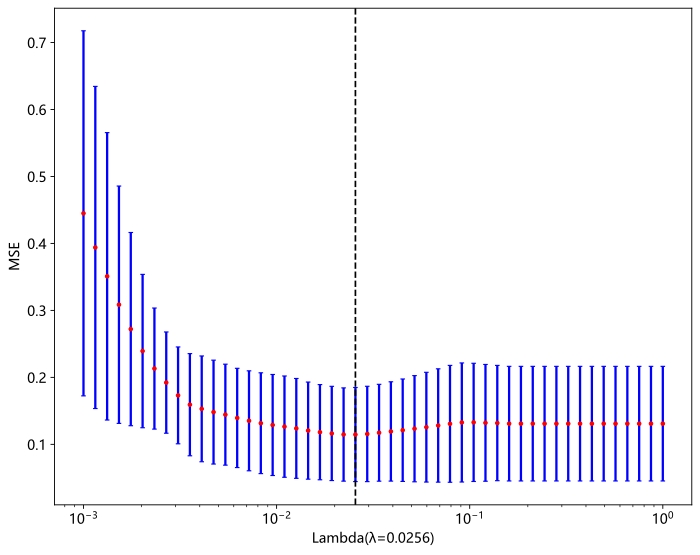


**B**


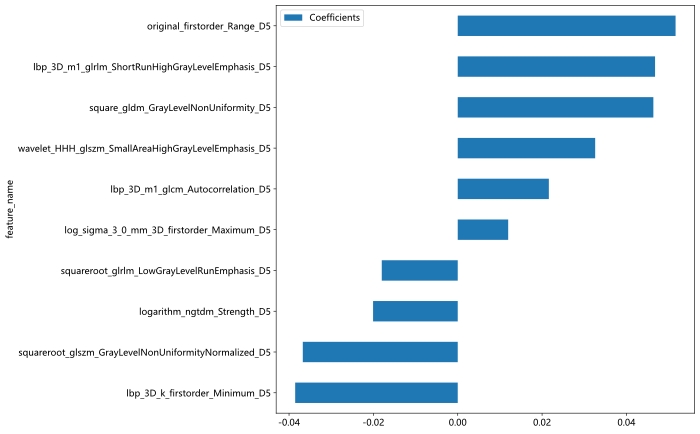


**C**


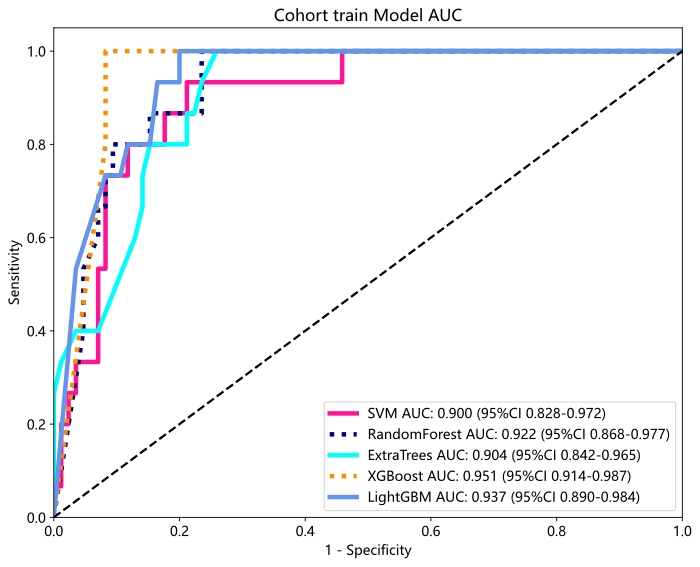


**D**


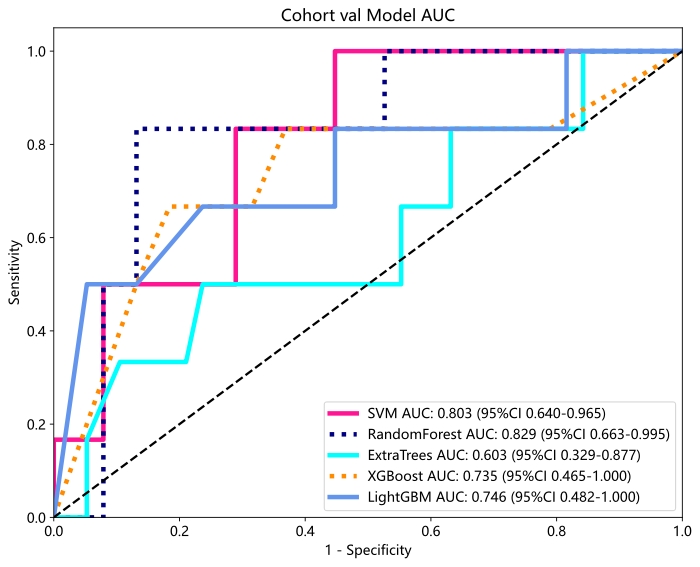


**E**


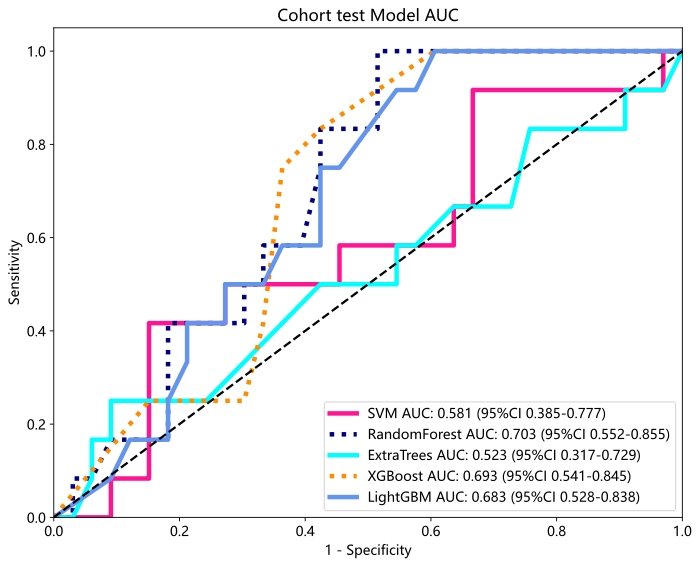


**F**

Figure S6. Coefficients(A), Mean standard error(B) and Weights(C) of 10 fold cross validation of D_5_ signatures, and ROC curves of machine learning models for D_5_ in train (D),validation(E), and testing cohort(F)

Table S6. Metrics of D_5_ in three different tasks

| Model_name | Accuracy | AUC | 95% CI | Sensitivity | Specificity | PPV | NPV | Task |
| --- | --- | --- | --- | --- | --- | --- | --- | --- |
| SVM | 0.800 | 0.900 | 0.8276 - 0.9716 | 0.867 | 0.788 | 0.419 | 0.971 | Train |
| SVM | 0.591 | 0.803 | 0.6404 - 0.9649 | 0.833 | 0.553 | 0.227 | 0.955 | Val |
| SVM | 0.711 | 0.581 | 0.3847 - 0.7769 | 0.333 | 0.848 | 0.444 | 0.778 | Test |
| RandomForest | 0.790 | 0.922 | 0.8680 - 0.9767 | 0.933 | 0.765 | 0.412 | 0.985 | Train |
| RandomForest | 0.841 | 0.829 | 0.6632 - 0.9947 | 0.667 | 0.868 | 0.444 | 0.943 | Val |
| RandomForest | 0.600 | 0.703 | 0.5517 - 0.8549 | 0.917 | 0.485 | 0.393 | 0.941 | Test |
| ExtraTrees | 0.790 | 0.904 | 0.8425 - 0.9653 | 0.933 | 0.765 | 0.412 | 0.985 | Train |
| ExtraTrees | 0.727 | 0.603 | 0.3289 - 0.8773 | 0.333 | 0.789 | 0.200 | 0.882 | Val |
| ExtraTrees | 0.711 | 0.523 | 0.3166 - 0.7288 | 0.167 | 0.909 | 0.400 | 0.750 | Test |
| XGBoost | 0.910 | 0.951 | 0.9143 - 0.9869 | 0.867 | 0.918 | 0.650 | 0.975 | Train |
| XGBoost | 0.818 | 0.735 | 0.4648 - 1.0000 | 0.500 | 0.868 | 0.375 | 0.917 | Val |
| XGBoost | 0.667 | 0.693 | 0.5409 - 0.8455 | 0.750 | 0.636 | 0.429 | 0.875 | Test |
| LightGBM | 0.820 | 0.937 | 0.8901 - 0.9836 | 0.933 | 0.800 | 0.452 | 0.986 | Train |
| LightGBM | 0.864 | 0.746 | 0.4819 - 1.0000 | 0.000 | 1.000 | 0.000 | 0.864 | Val |
| LightGBM | 0.556 | 0.683 | 0.5278 - 0.8384 | 0.917 | 0.424 | 0.367 | 0.933 | Test |

*Abbreviation: AUC:areas under the curve;CI:confidence interval;PPV:positive predictive value; NPV:negative predictive value;SVM:support vector machine.*


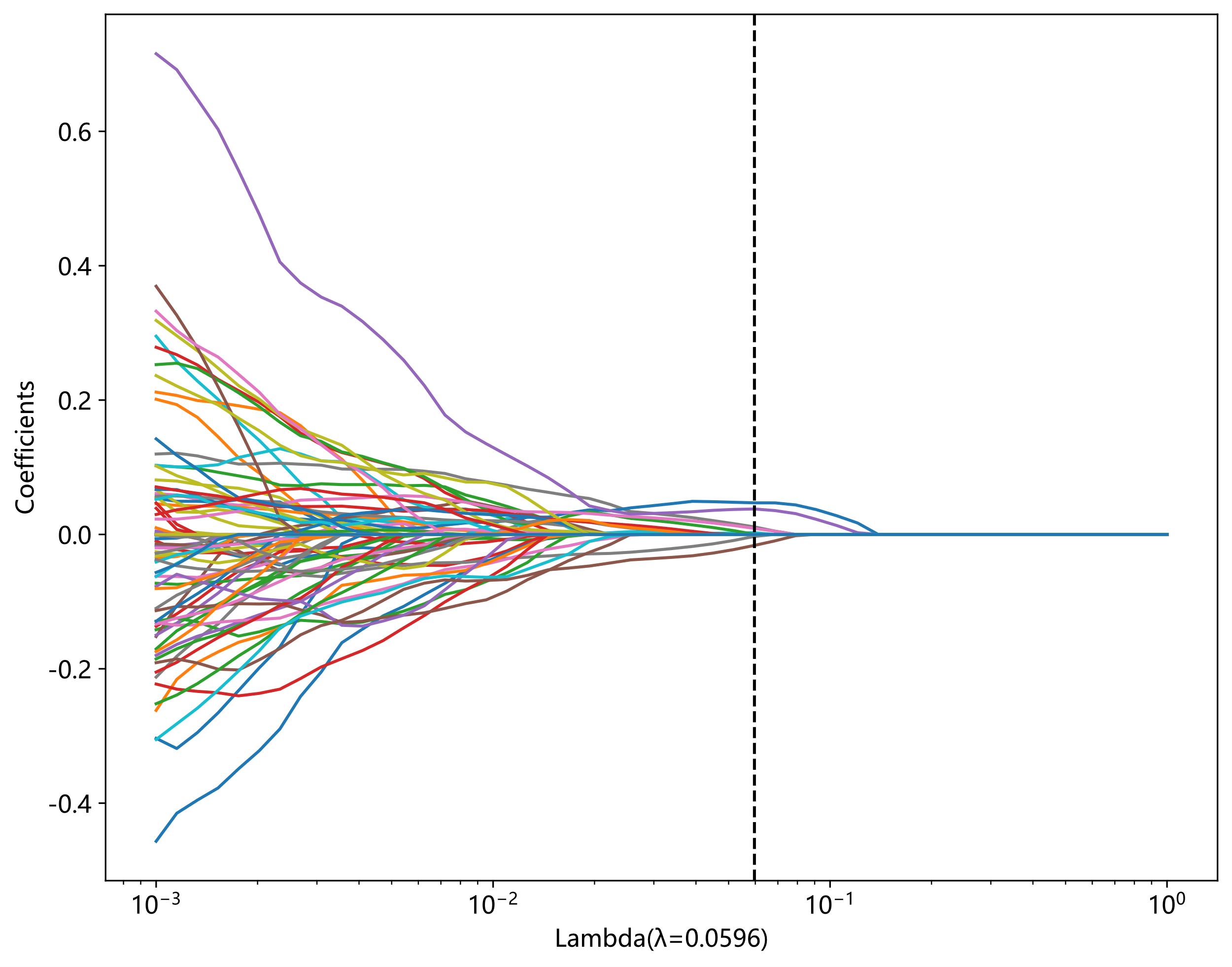


**A**


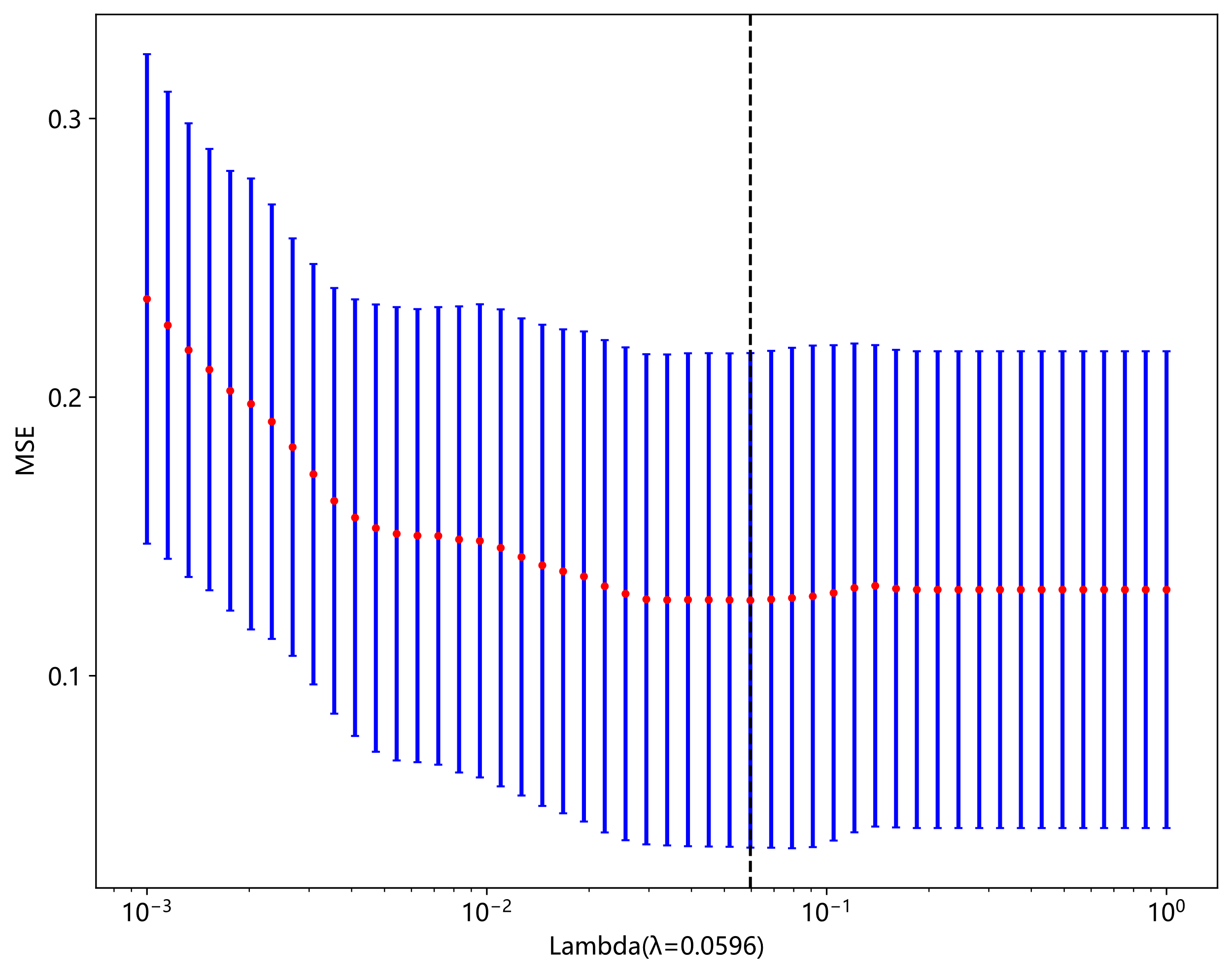


**B**


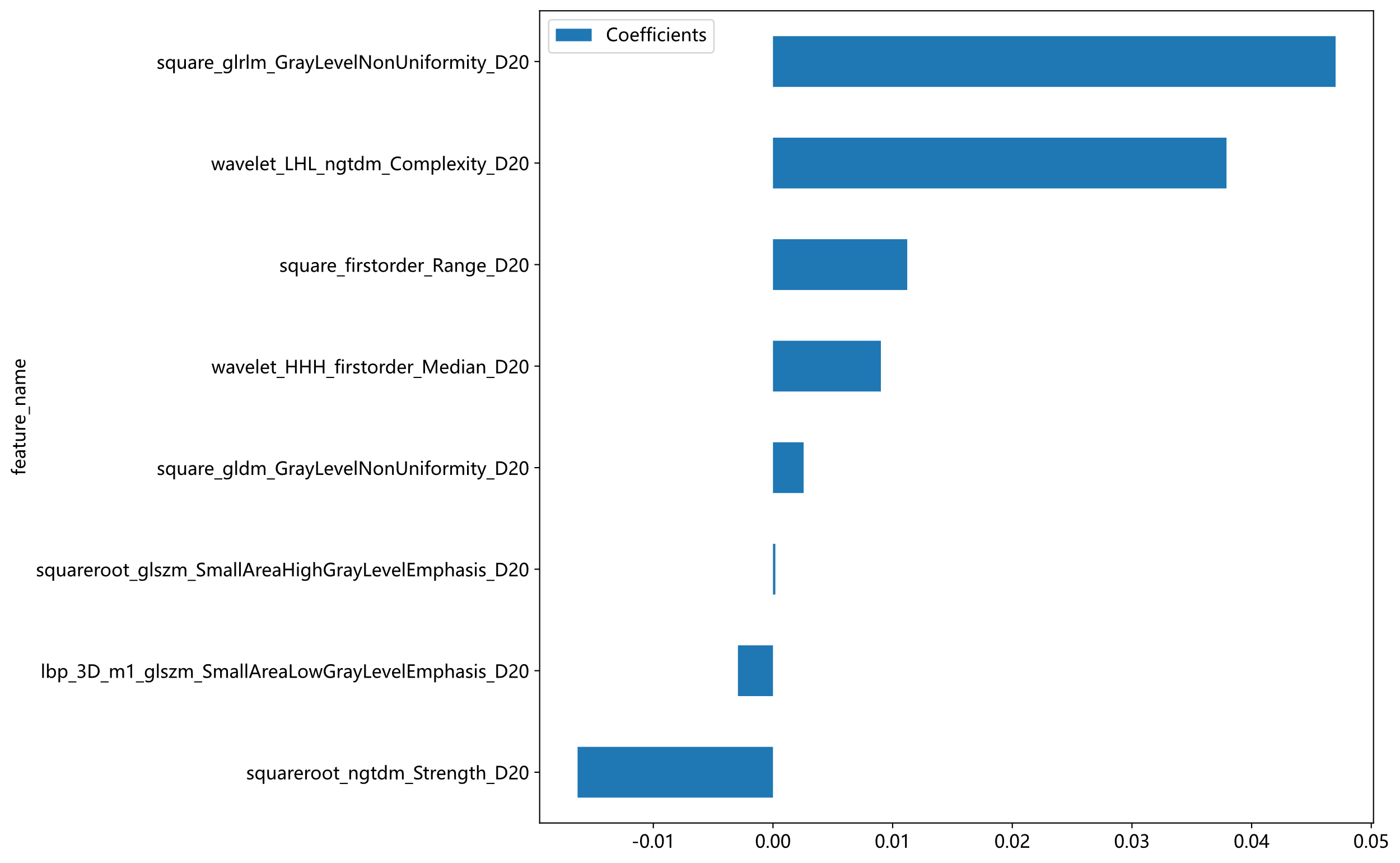


**C**


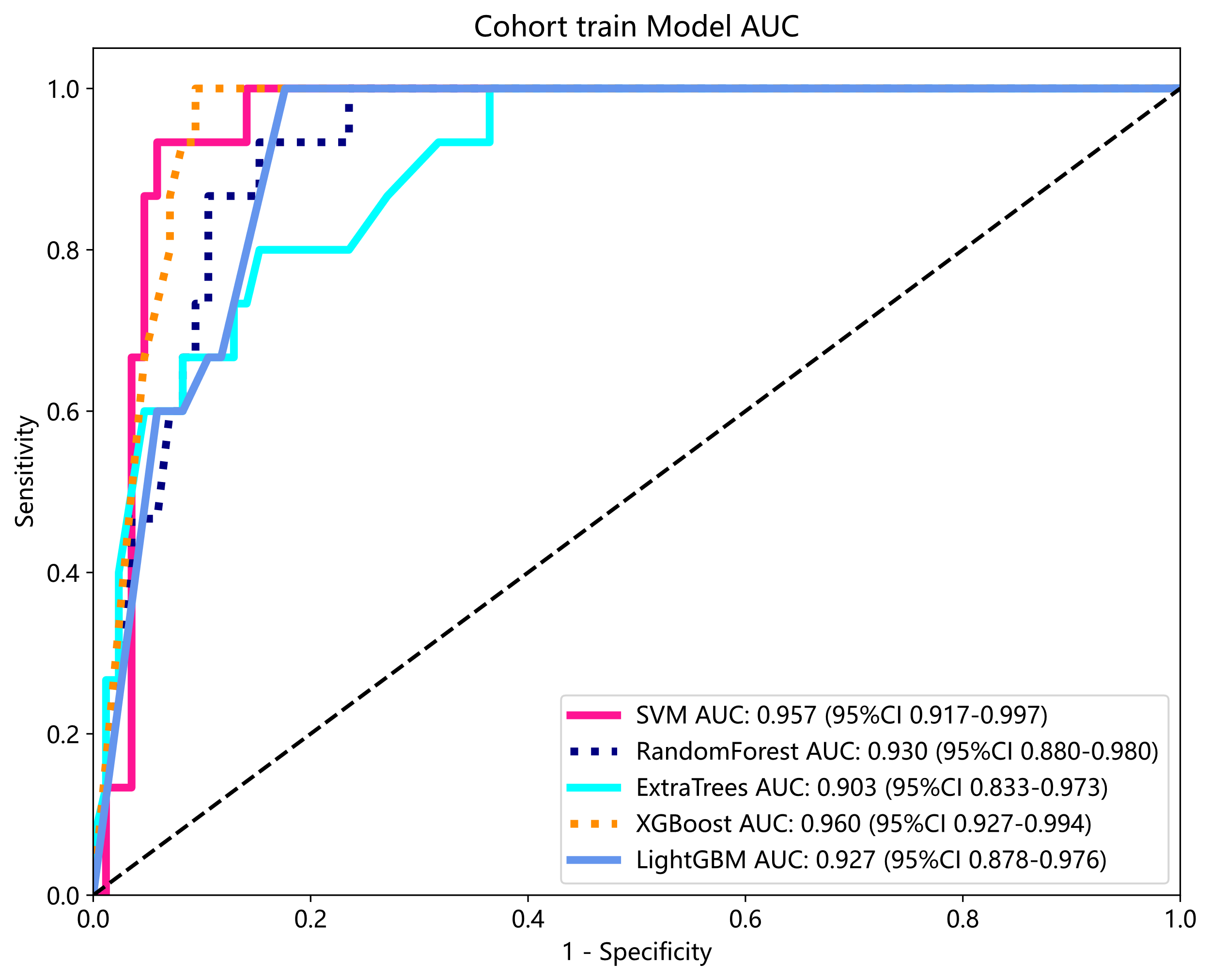


**D**


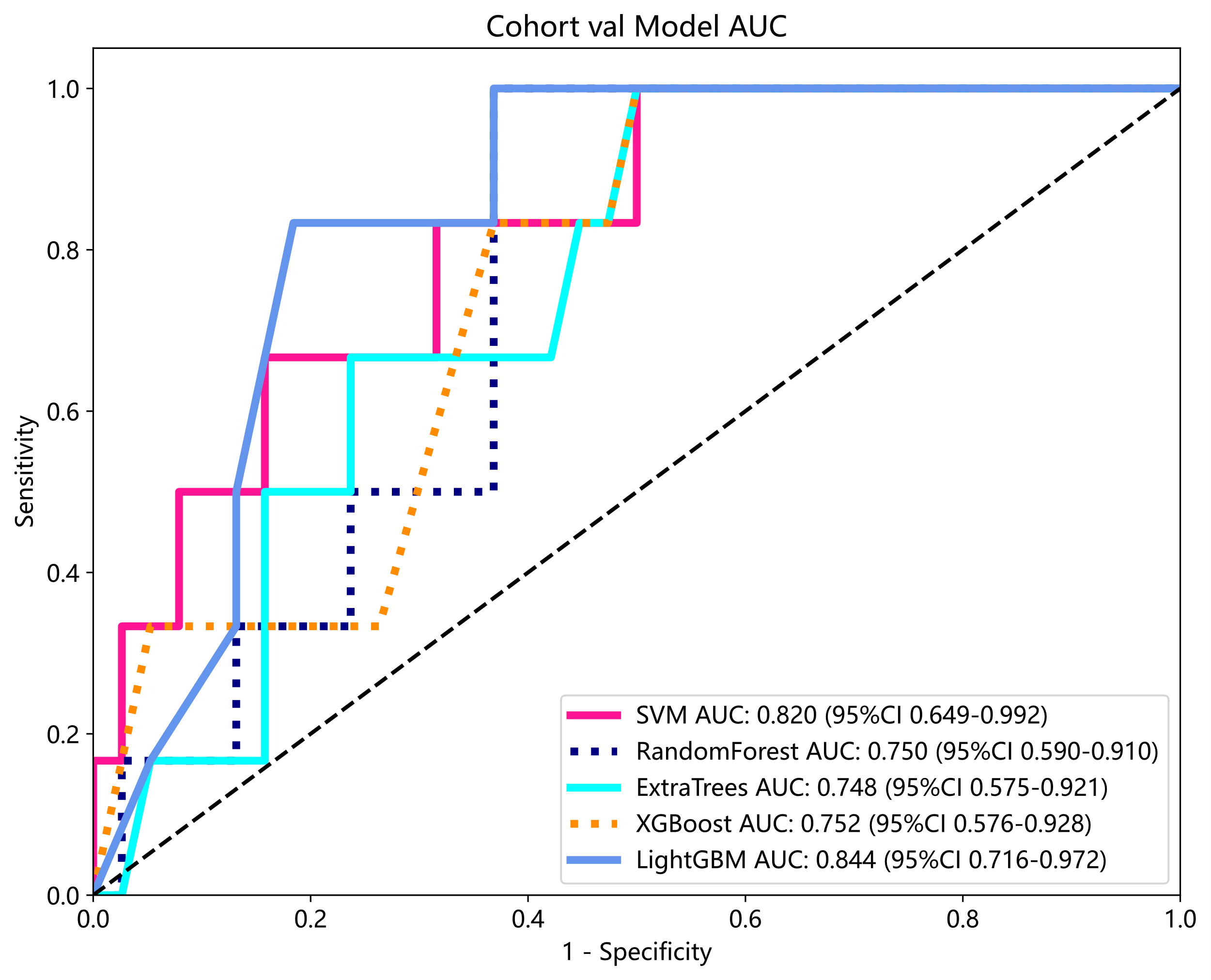


**E**


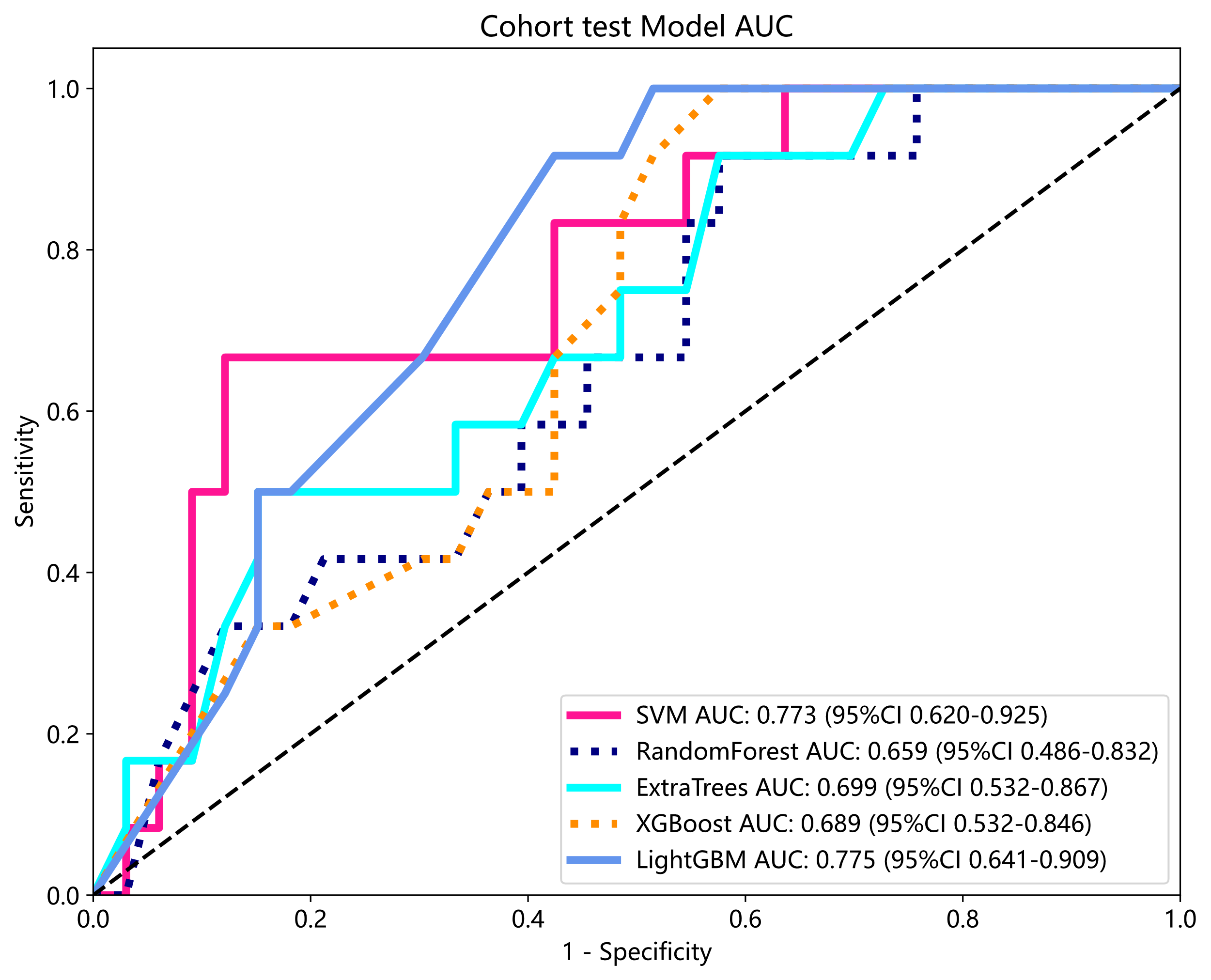


**F**

Figure S7. Coefficients(A), Mean standard error(B) and Weights(C) of 10 fold cross validation of D_20_ signatures, and ROC curves of machine learning models for D_20_ in train (D),validation(E), and testing cohort(F)

Table S7. Metrics of D_20_ in three different tasks

| Model_name | Accuracy | AUC | 95% CI | Sensitivity | Specificity | PPV | NPV | Task |
| --- | --- | --- | --- | --- | --- | --- | --- | --- |
| SVM | 0.930 | 0.957 | 0.9172 - 0.9965 | 0.867 | 0.941 | 0.722 | 0.976 | Train |
| SVM | 0.682 | 0.820 | 0.6487 - 0.9916 | 0.667 | 0.684 | 0.250 | 0.929 | Val |
| SVM | 0.800 | 0.773 | 0.6201 - 0.9253 | 0.583 | 0.879 | 0.636 | 0.853 | Test |
| RandomForest | 0.850 | 0.930 | 0.8804 - 0.9800 | 0.867 | 0.847 | 0.500 | 0.973 | Train |
| RandomForest | 0.659 | 0.750 | 0.5896 - 0.9104 | 0.833 | 0.632 | 0.263 | 0.960 | Val |
| RandomForest | 0.533 | 0.659 | 0.4858 - 0.8324 | 0.833 | 0.424 | 0.345 | 0.875 | Test |
| ExtraTrees | 0.840 | 0.903 | 0.8332 - 0.9731 | 0.733 | 0.859 | 0.478 | 0.948 | Train |
| ExtraTrees | 0.568 | 0.748 | 0.5750 - 0.9206 | 0.833 | 0.526 | 0.217 | 0.952 | Val |
| ExtraTrees | 0.733 | 0.699 | 0.5319 - 0.8671 | 0.417 | 0.848 | 0.500 | 0.800 | Test |
| XGBoost | 0.910 | 0.960 | 0.9268 - 0.9940 | 0.933 | 0.906 | 0.636 | 0.987 | Train |
| XGBoost | 0.568 | 0.752 | 0.5761 - 0.9283 | 0.833 | 0.526 | 0.217 | 0.952 | Val |
| XGBoost | 0.600 | 0.689 | 0.5324 - 0.8464 | 0.917 | 0.485 | 0.393 | 0.941 | Test |
| LightGBM | 0.850 | 0.927 | 0.8782 - 0.9759 | 0.800 | 0.859 | 0.500 | 0.961 | Train |
| LightGBM | 0.818 | 0.844 | 0.7164 - 0.9722 | 0.500 | 0.868 | 0.375 | 0.917 | Val |
| LightGBM | 0.689 | 0.775 | 0.6414 - 0.9091 | 0.667 | 0.697 | 0.444 | 0.852 | Test |

*Abbreviation: AUC:areas under the curve;CI:confidence interval;PPV:positive predictive value; NPV:negative predictive value;SVM:support vector machine..*


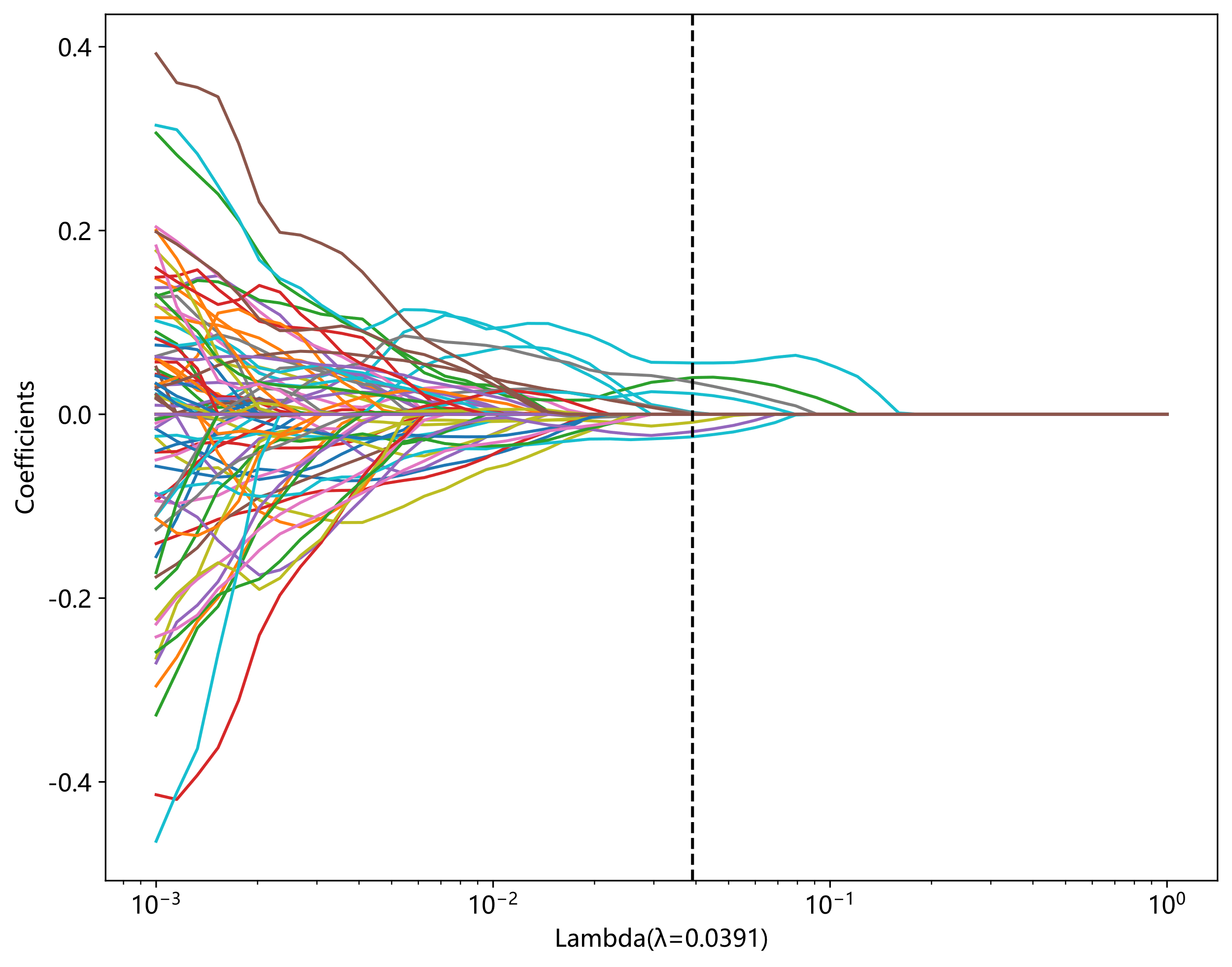


**A**


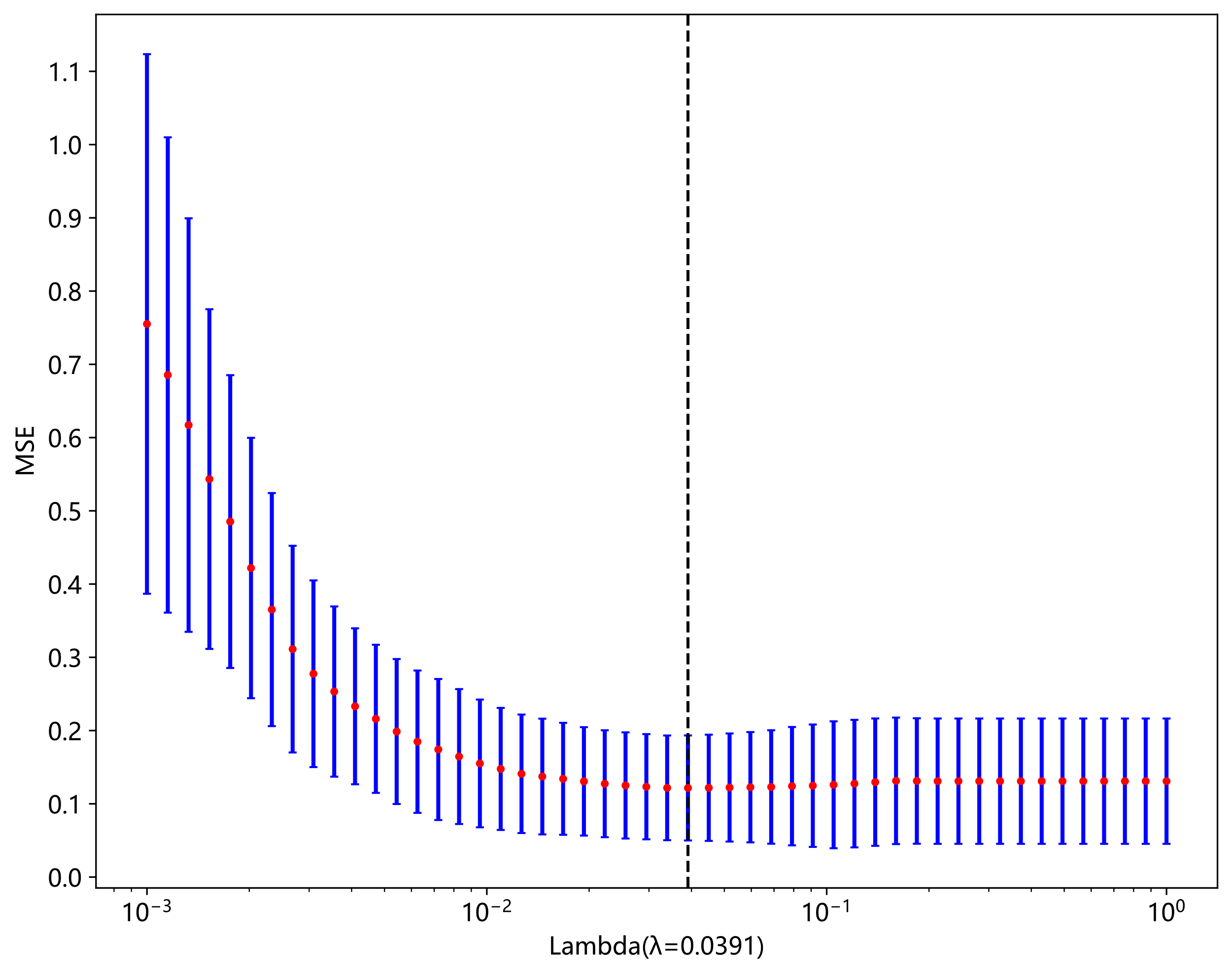


**B**


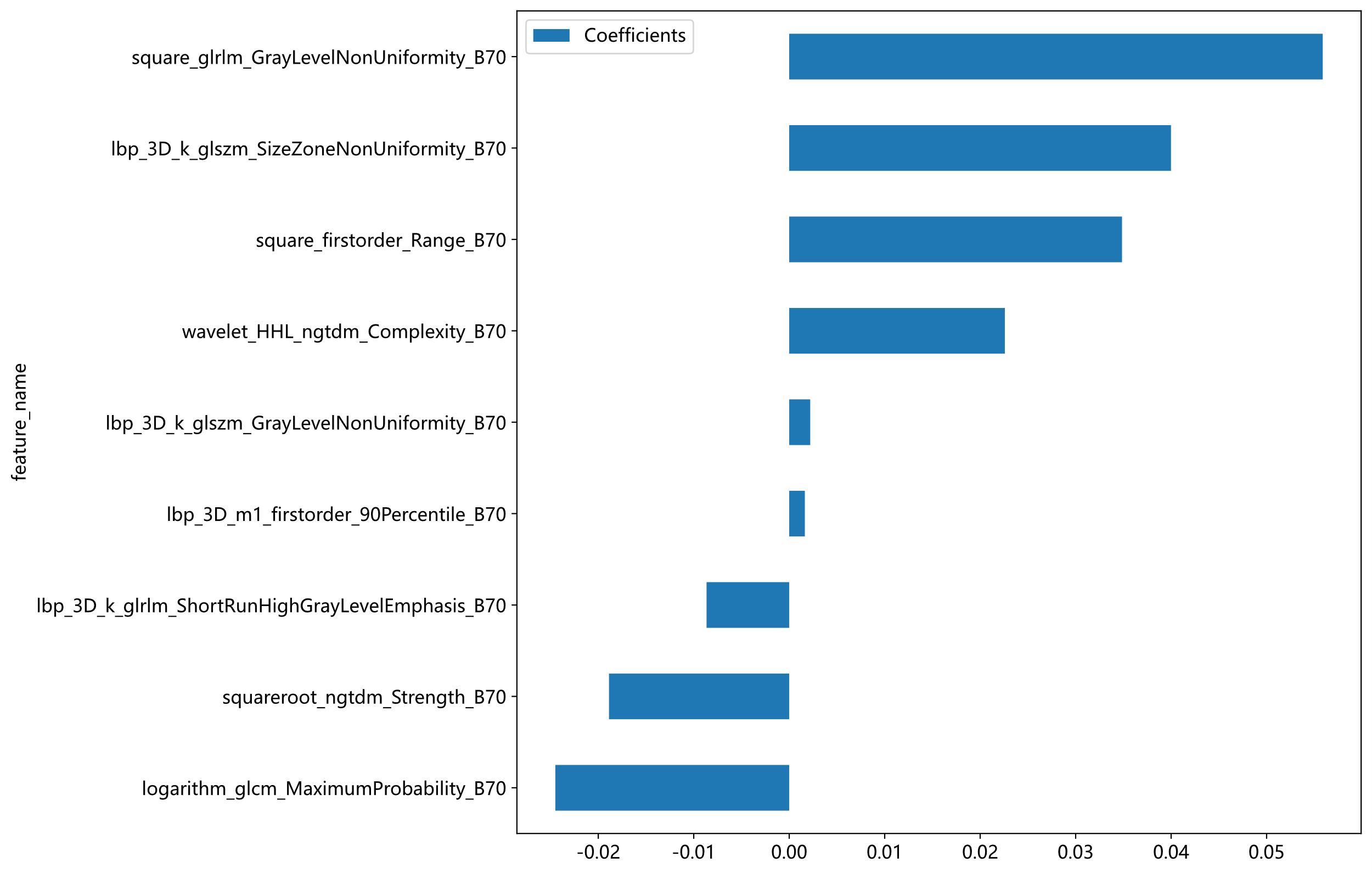


**C**


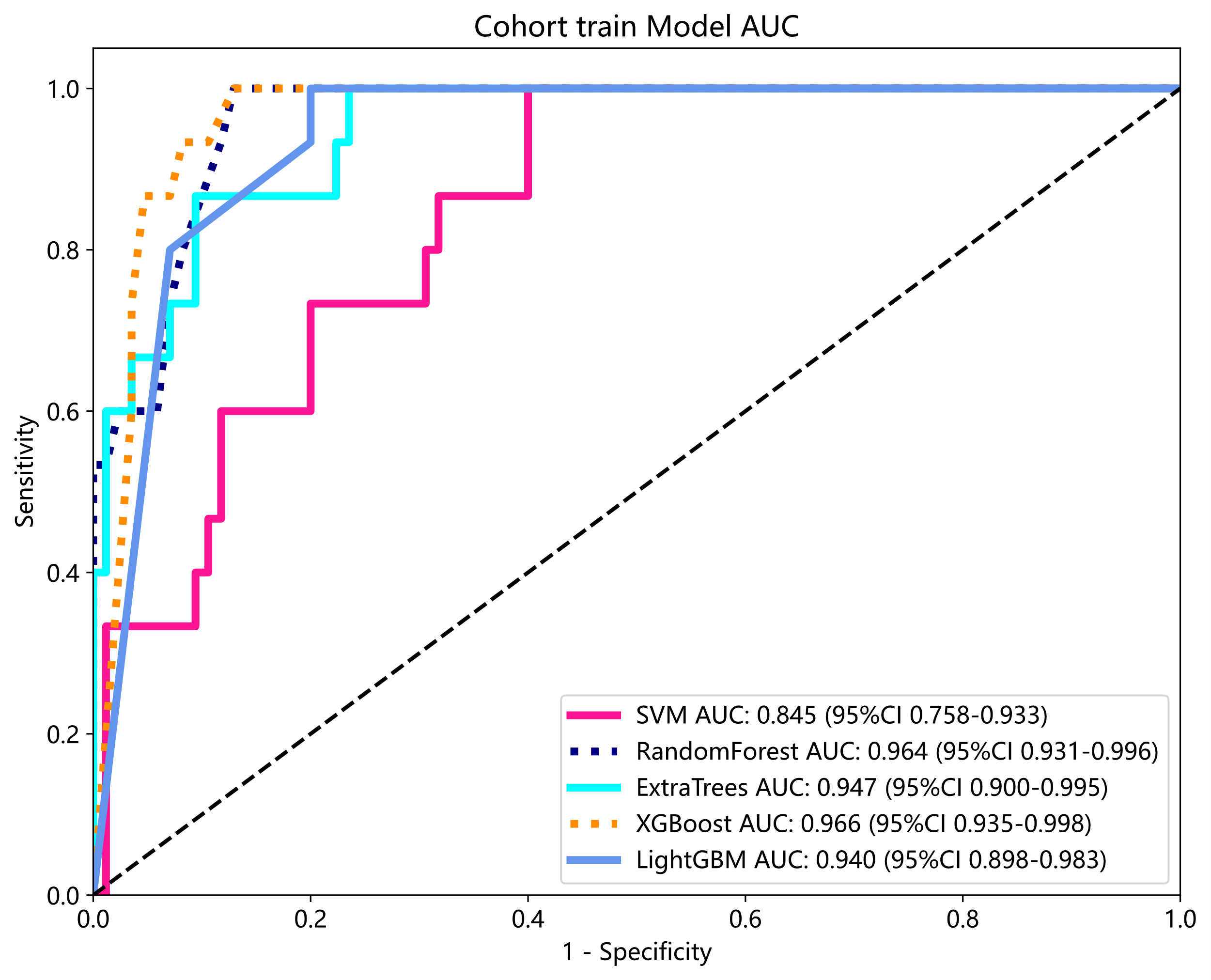


**D**


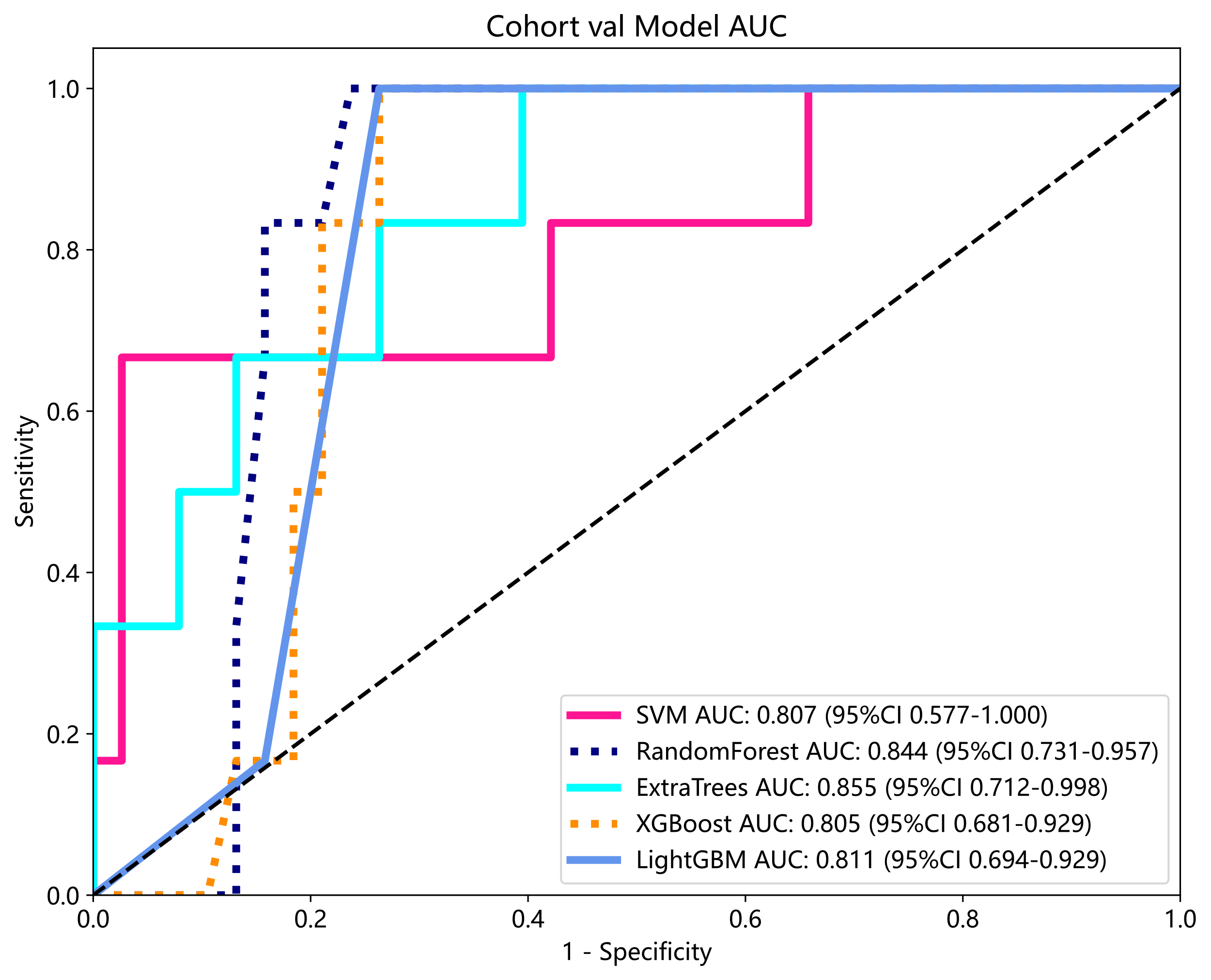


**E**


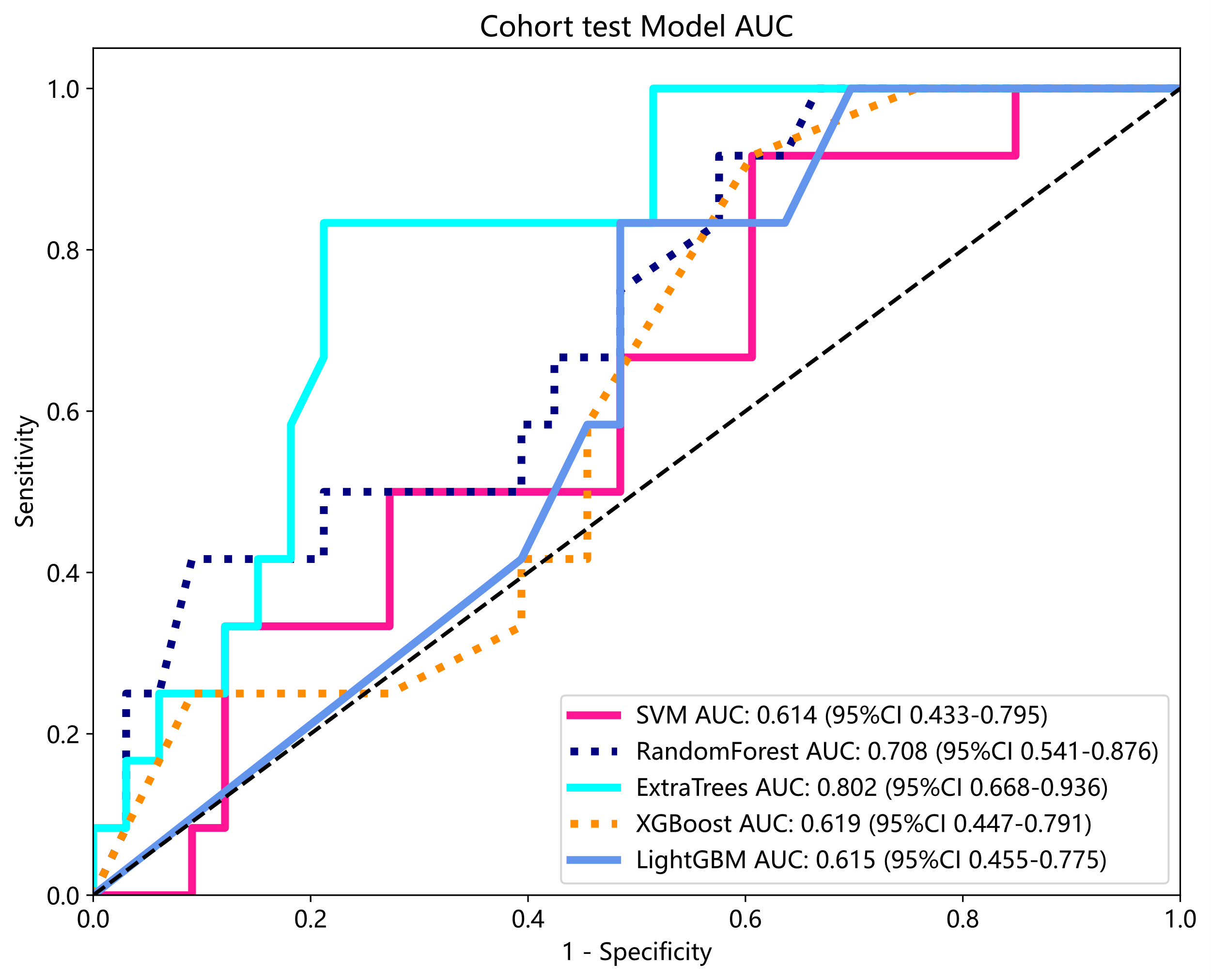


**F**

Figure S8. Coefficients(A), Mean standard error(B) and Weights(C) of 10 fold cross validation of B_70_ signatures, and ROC curves of machine learning models for B_70_ in train (D),validation(E), and testing cohort(F)

Table S8. Metrics of B70 in three different tasks

| Model_name | Accuracy | AUC | 95% CI | Sensitivity | Specificity | PPV | NPV | Task |
| --- | --- | --- | --- | --- | --- | --- | --- | --- |
| SVM | 0.650 | 0.845 | 0.7577 - 0.9333 | 0.933 | 0.600 | 0.292 | 0.981 | Train |
| SVM | 0.909 | 0.807 | 0.5767 - 1.0000 | 0.500 | 0.974 | 0.750 | 0.925 | Val |
| SVM | 0.511 | 0.614 | 0.4327 - 0.7946 | 0.833 | 0.394 | 0.333 | 0.867 | Test |
| RandomForest | 0.890 | 0.964 | 0.9307 - 0.9964 | 0.933 | 0.882 | 0.583 | 0.987 | Train |
| RandomForest | 0.795 | 0.844 | 0.7313 - 0.9573 | 0.833 | 0.789 | 0.385 | 0.968 | Val |
| RandomForest | 0.533 | 0.708 | 0.5412 - 0.8755 | 0.833 | 0.424 | 0.345 | 0.875 | Test |
| ExtraTrees | 0.890 | 0.947 | 0.9004 - 0.9945 | 0.800 | 0.906 | 0.600 | 0.962 | Train |
| ExtraTrees | 0.636 | 0.855 | 0.7122 - 0.9983 | 0.833 | 0.605 | 0.250 | 0.958 | Val |
| ExtraTrees | 0.778 | 0.802 | 0.6678 - 0.9358 | 0.750 | 0.788 | 0.562 | 0.897 | Test |
| XGBoost | 0.900 | 0.966 | 0.9347 - 0.9979 | 0.933 | 0.894 | 0.609 | 0.987 | Train |
| XGBoost | 0.750 | 0.805 | 0.6810 - 0.9287 | 0.833 | 0.737 | 0.333 | 0.966 | Val |
| XGBoost | 0.556 | 0.619 | 0.4467 - 0.7906 | 0.583 | 0.545 | 0.318 | 0.783 | Test |
| LightGBM | 0.820 | 0.940 | 0.8979 - 0.9829 | 0.933 | 0.800 | 0.452 | 0.986 | Train |
| LightGBM | 0.750 | 0.811 | 0.6938 - 0.9290 | 0.167 | 0.842 | 0.143 | 0.865 | Val |
| LightGBM | 0.533 | 0.615 | 0.4550 - 0.7748 | 0.583 | 0.515 | 0.304 | 0.773 | Test |

*Abbreviation: AUC:areas under the curve;CI:confidence interval;PPV:positive predictive value; NPV:negative predictive value;SVM:support vector machine.*


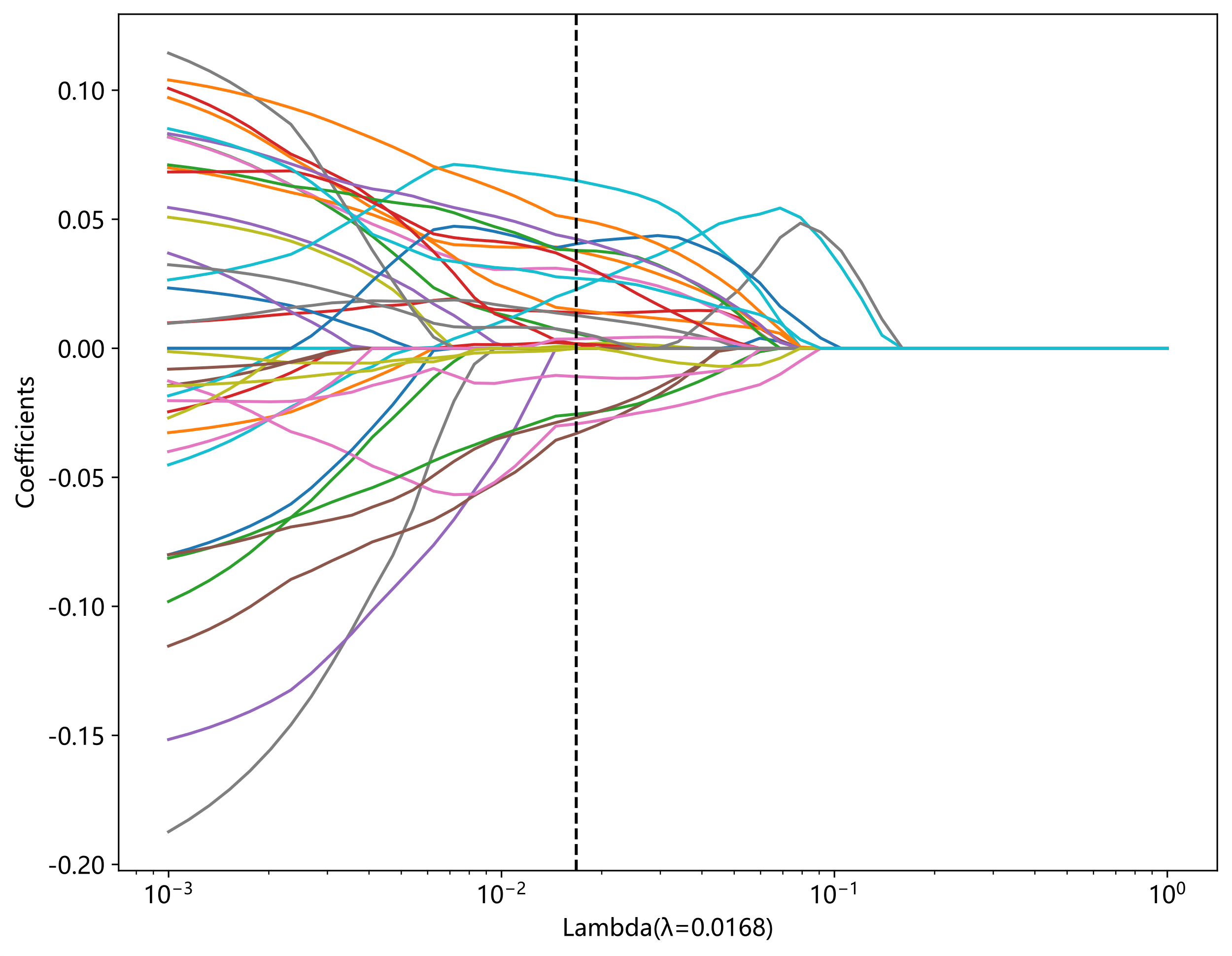


**A**


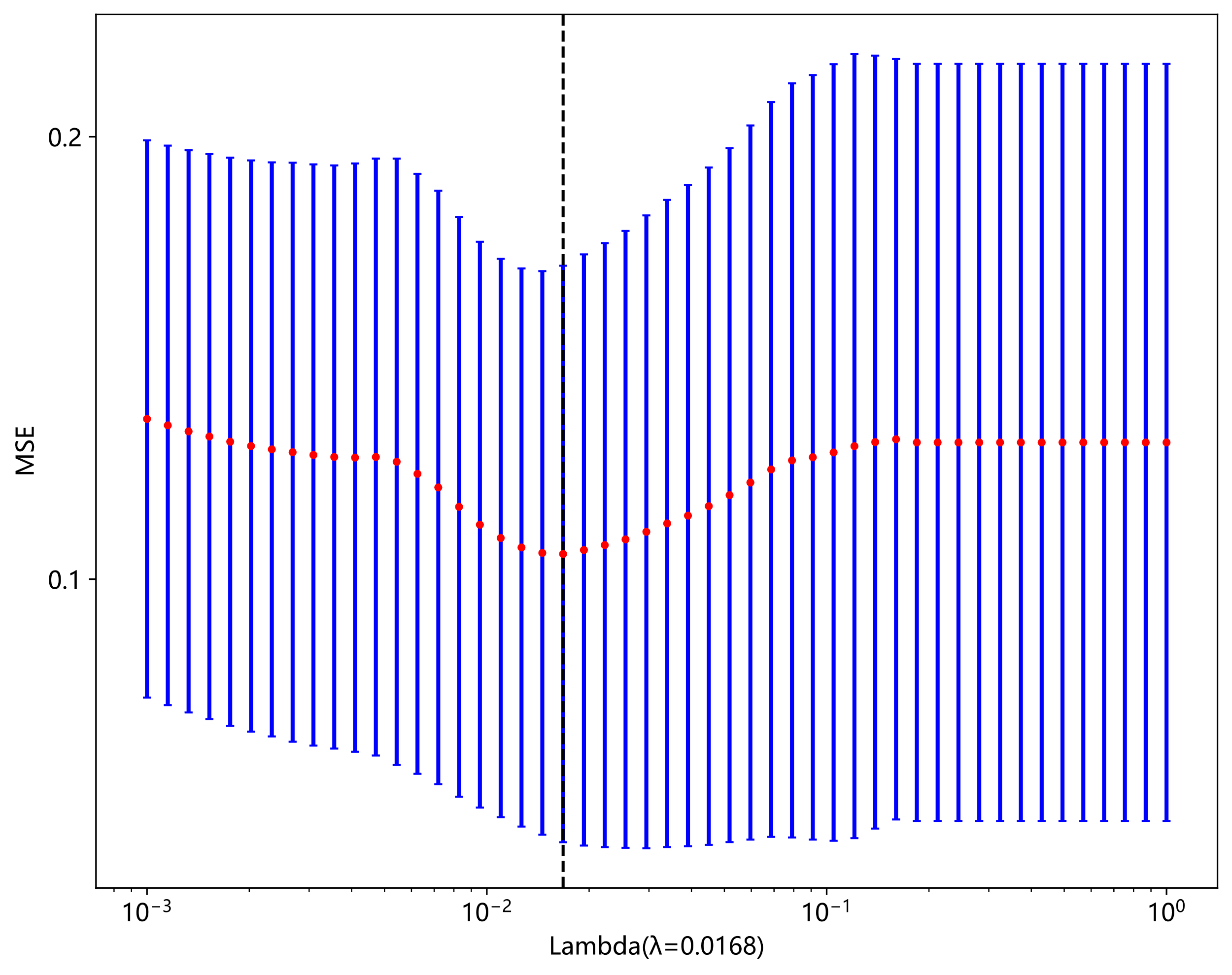


**B**


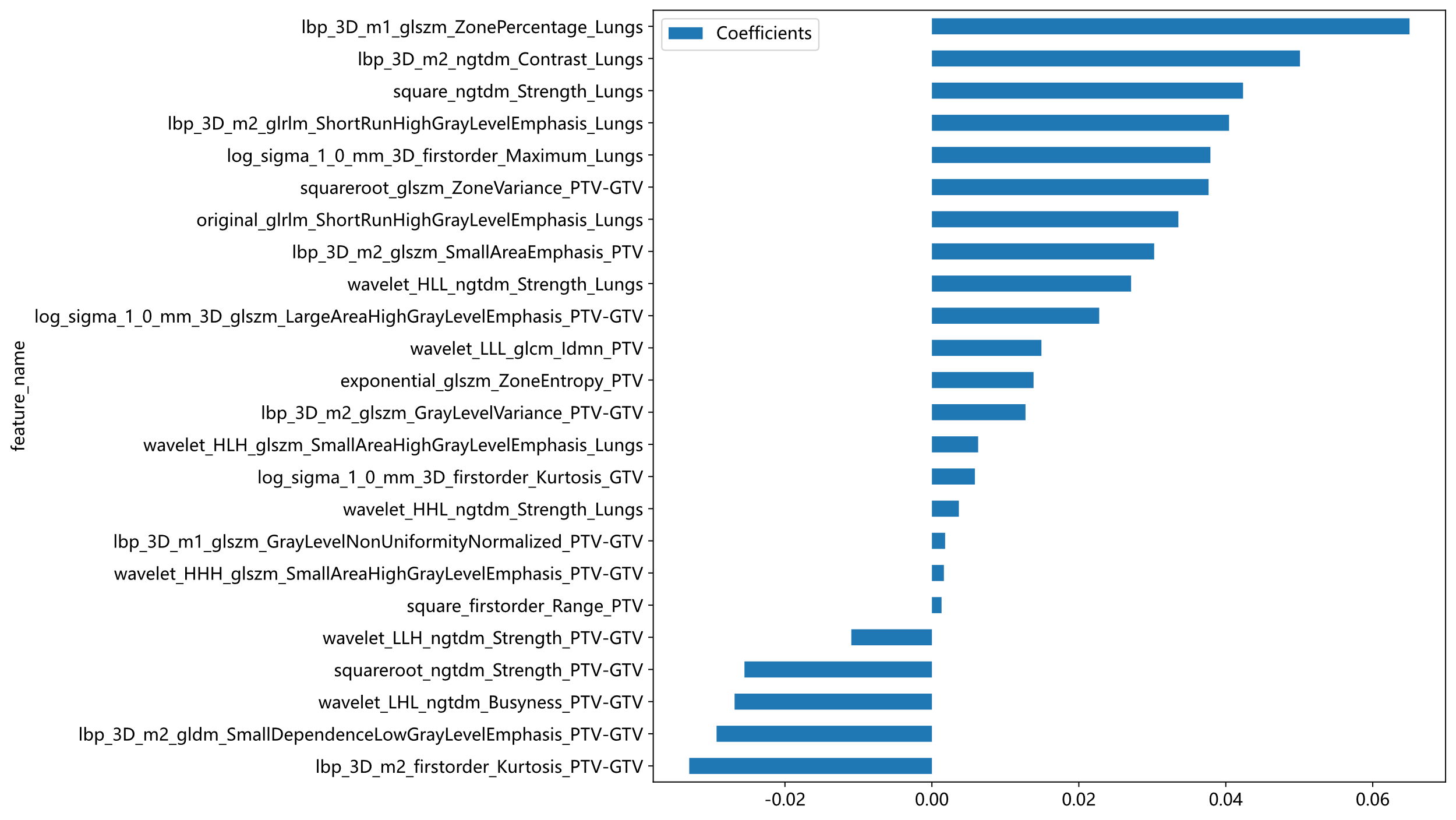


**C**


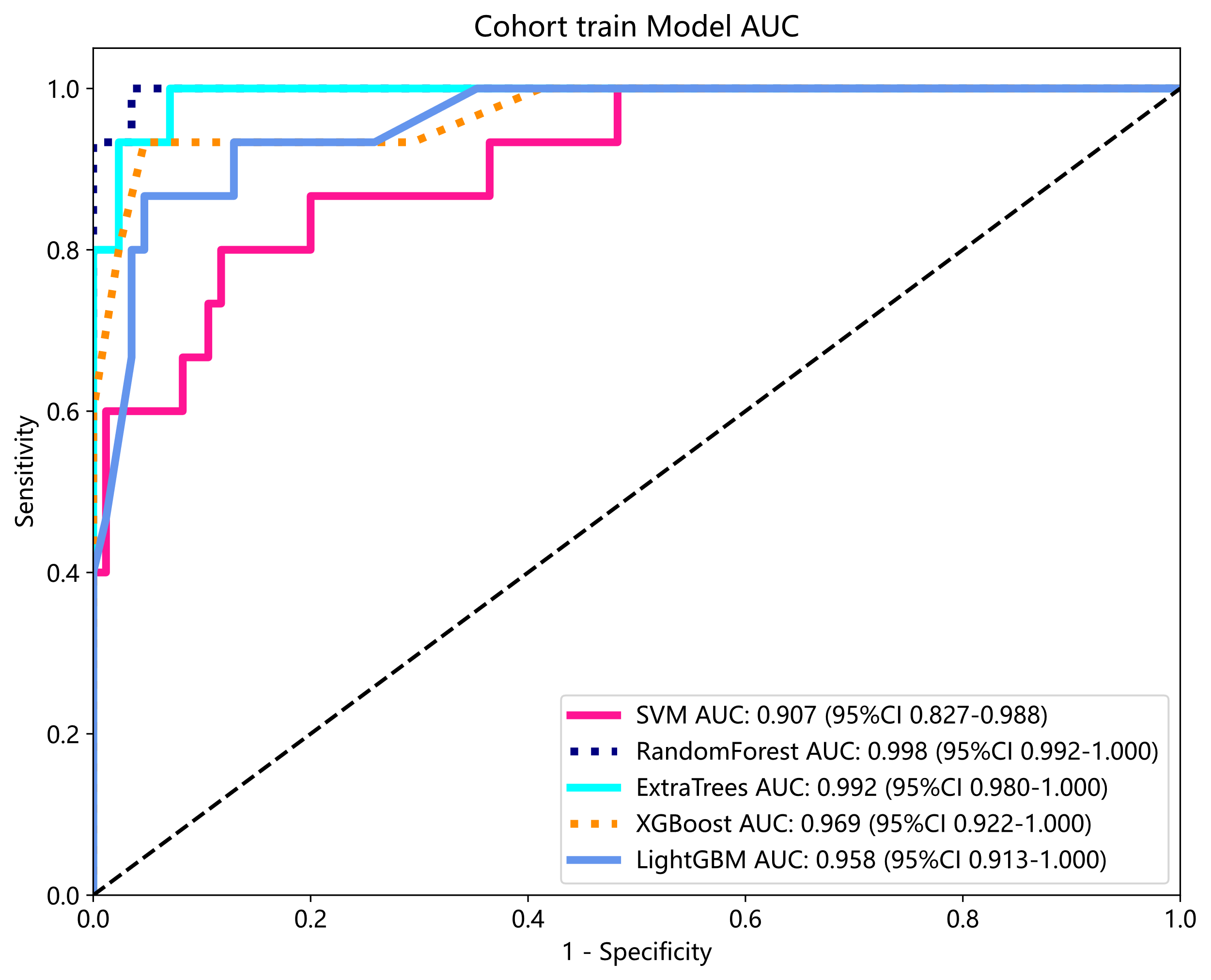


**D**


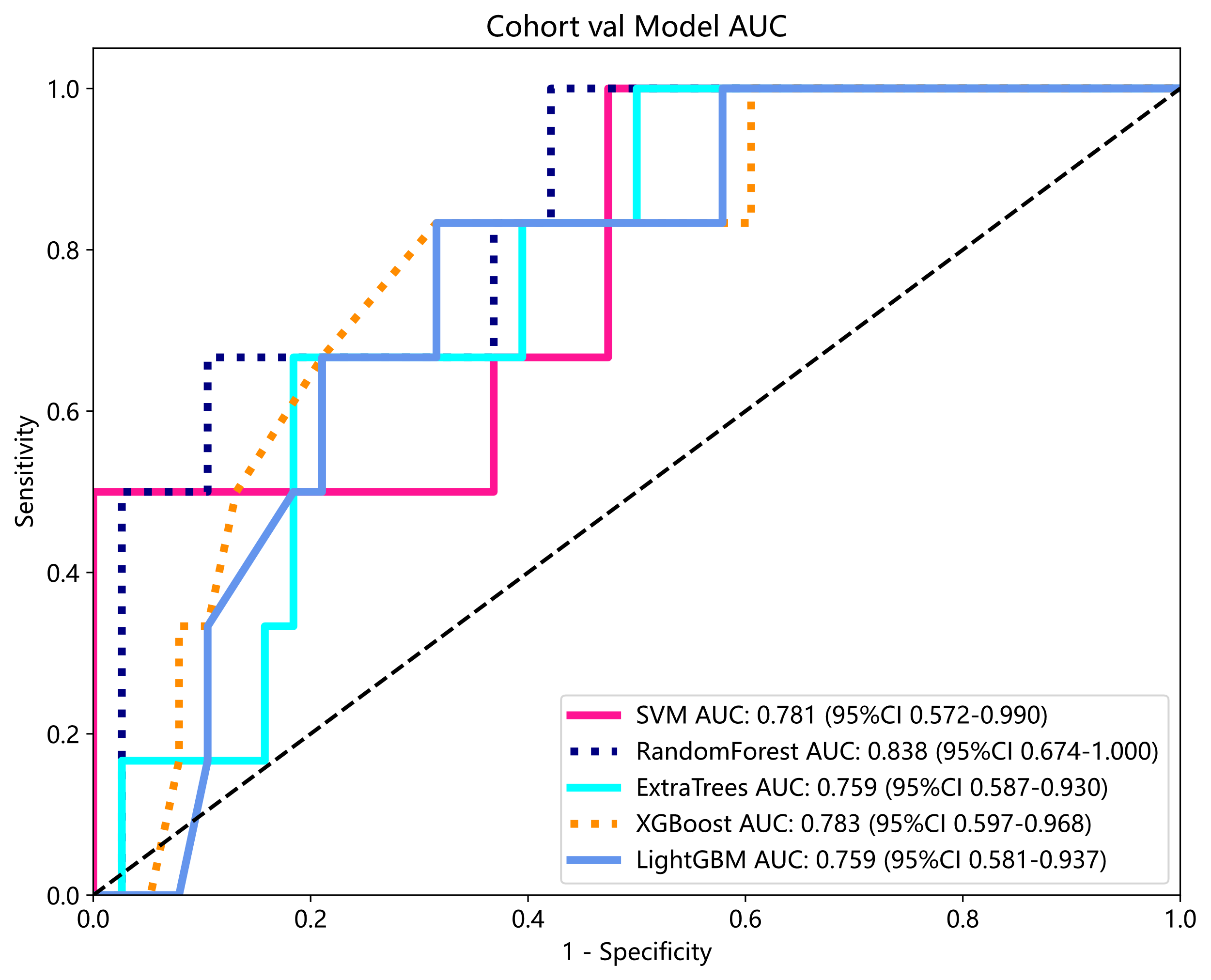


**E**


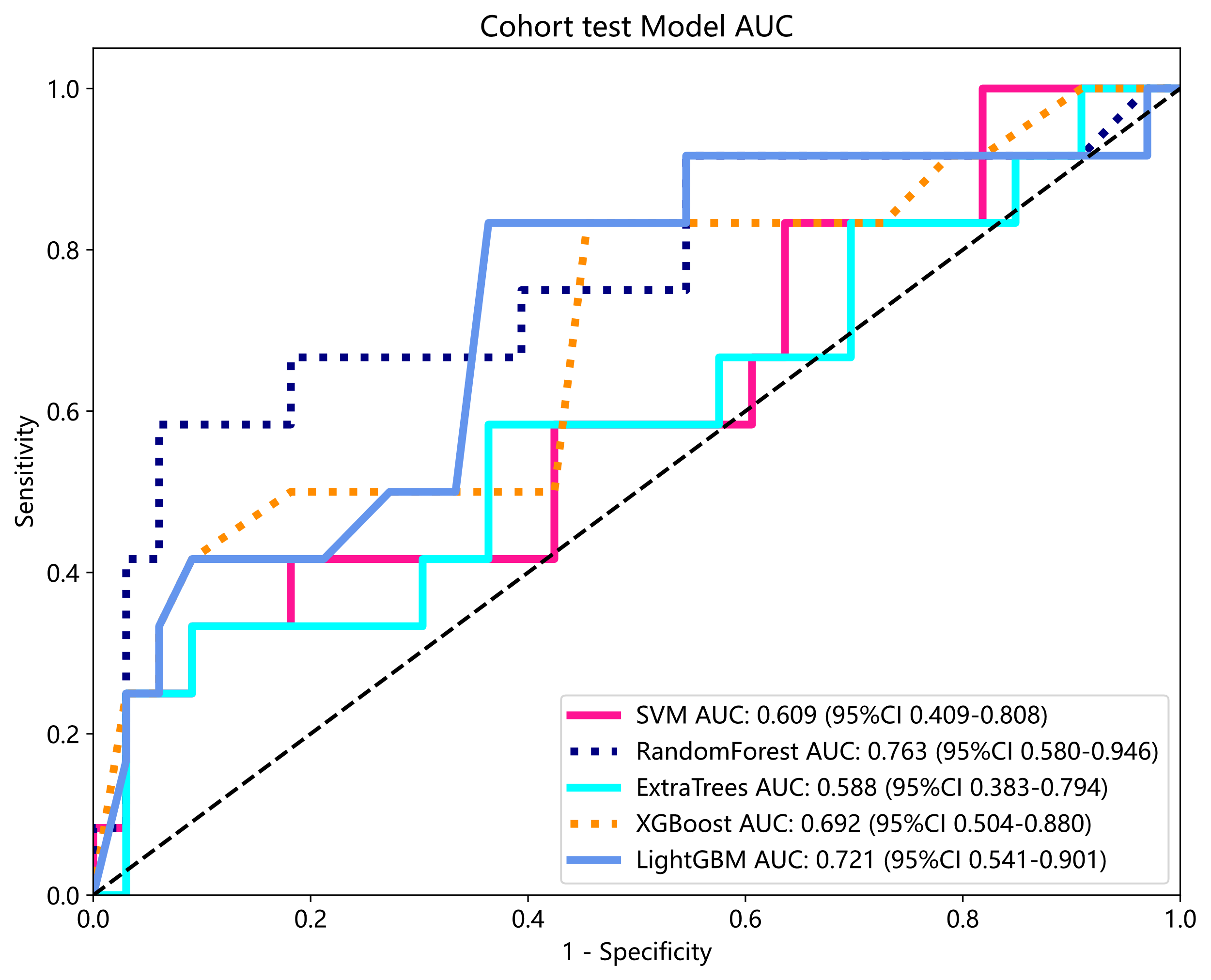


**F**

Figure S9. Coefficients(A), Mean standard error(B) and Weights(C) of 10 fold cross validation of RA signatures, and ROC curves of machine learning models for RA in train (D),validation(E), and testing cohort(F)

*Abbreviation: RA:regions of anatomical demension*

Table S9. Metrics of RA in three different tasks

| Model_name | Accuracy | AUC | 95% CI | Sensitivity | Specificity | PPV | NPV | Task |
| --- | --- | --- | --- | --- | --- | --- | --- | --- |
| SVM | 0.860 | 0.907 | 0.8272 - 0.9877 | 0.733 | 0.882 | 0.524 | 0.949 | Train |
| SVM | 0.568 | 0.781 | 0.5717 - 0.9897 | 0.833 | 0.526 | 0.217 | 0.952 | Val |
| SVM | 0.733 | 0.609 | 0.4092 - 0.8080 | 0.250 | 0.909 | 0.500 | 0.769 | Test |
| RandomForest | 0.960 | 0.998 | 0.9923 - 1.0000 | 0.933 | 0.965 | 0.824 | 0.988 | Train |
| RandomForest | 0.614 | 0.838 | 0.6741 - 1.0000 | 0.833 | 0.579 | 0.238 | 0.957 | Val |
| RandomForest | 0.822 | 0.763 | 0.5797 - 0.9455 | 0.500 | 0.939 | 0.750 | 0.838 | Test |
| ExtraTrees | 0.930 | 0.992 | 0.9801 - 1.0000 | 0.933 | 0.929 | 0.700 | 0.987 | Train |
| ExtraTrees | 0.545 | 0.759 | 0.5874 - 0.9301 | 0.833 | 0.500 | 0.208 | 0.950 | Val |
| ExtraTrees | 0.733 | 0.588 | 0.3829 - 0.7939 | 0.250 | 0.909 | 0.500 | 0.769 | Test |
| XGBoost | 0.950 | 0.969 | 0.9225 - 1.0000 | 0.800 | 0.976 | 0.857 | 0.965 | Train |
| XGBoost | 0.773 | 0.783 | 0.5974 - 0.9684 | 0.667 | 0.789 | 0.333 | 0.937 | Val |
| XGBoost | 0.556 | 0.692 | 0.5038 - 0.8800 | 0.500 | 0.576 | 0.300 | 0.760 | Test |
| LightGBM | 0.930 | 0.958 | 0.9126 - 1.0000 | 0.800 | 0.953 | 0.750 | 0.964 | Train |
| LightGBM | 0.682 | 0.759 | 0.5806 - 0.9369 | 0.667 | 0.684 | 0.250 | 0.929 | Val |
| LightGBM | 0.622 | 0.721 | 0.5409 - 0.9011 | 0.500 | 0.667 | 0.353 | 0.786 | Test |

*Abbreviation: RA:regions of anatomical demension; AUC:areas under the curve;CI:confidence interval;PPV:positive predictive value; NPV:negative predictive value;SVM:support vector machine.*


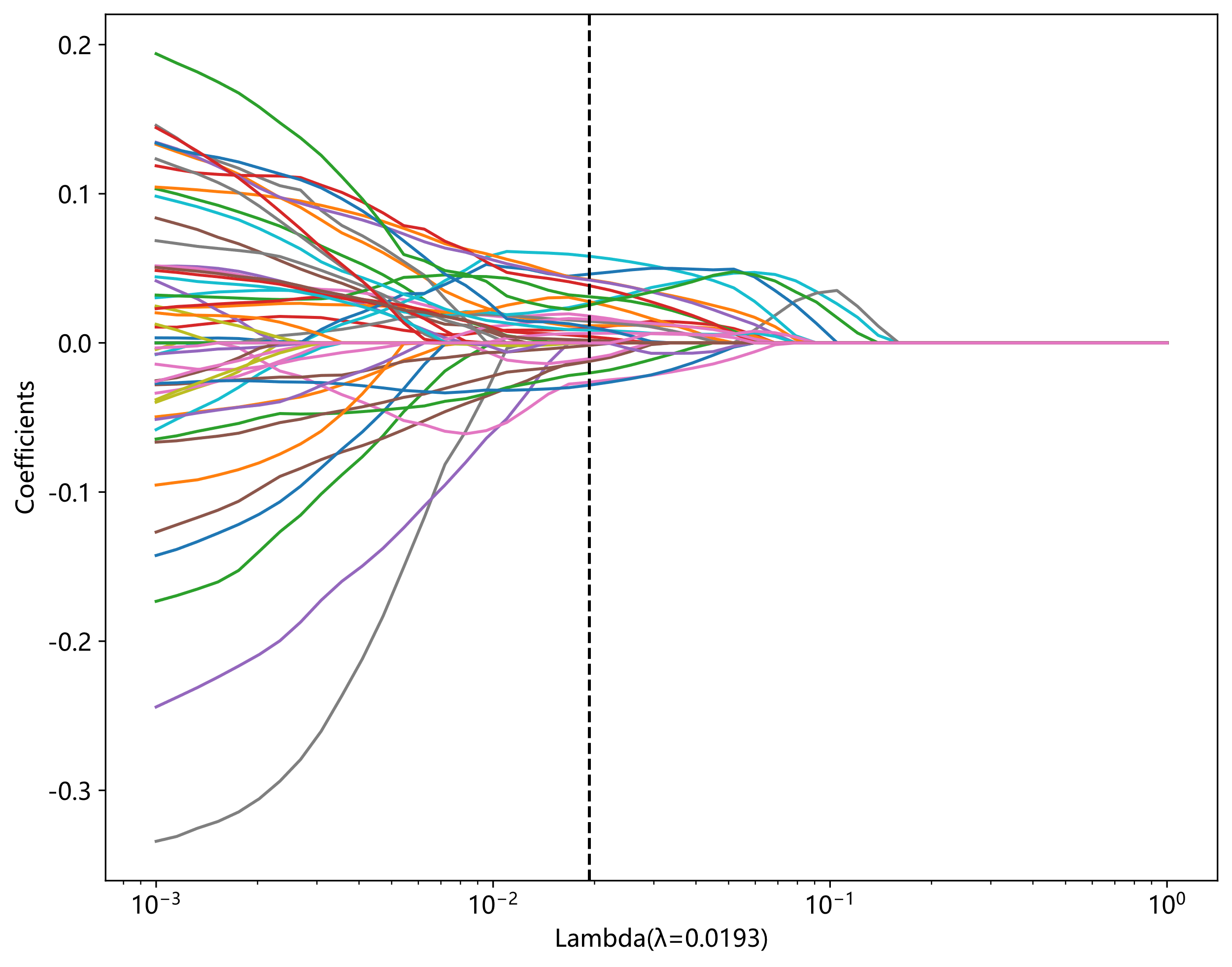


**A**


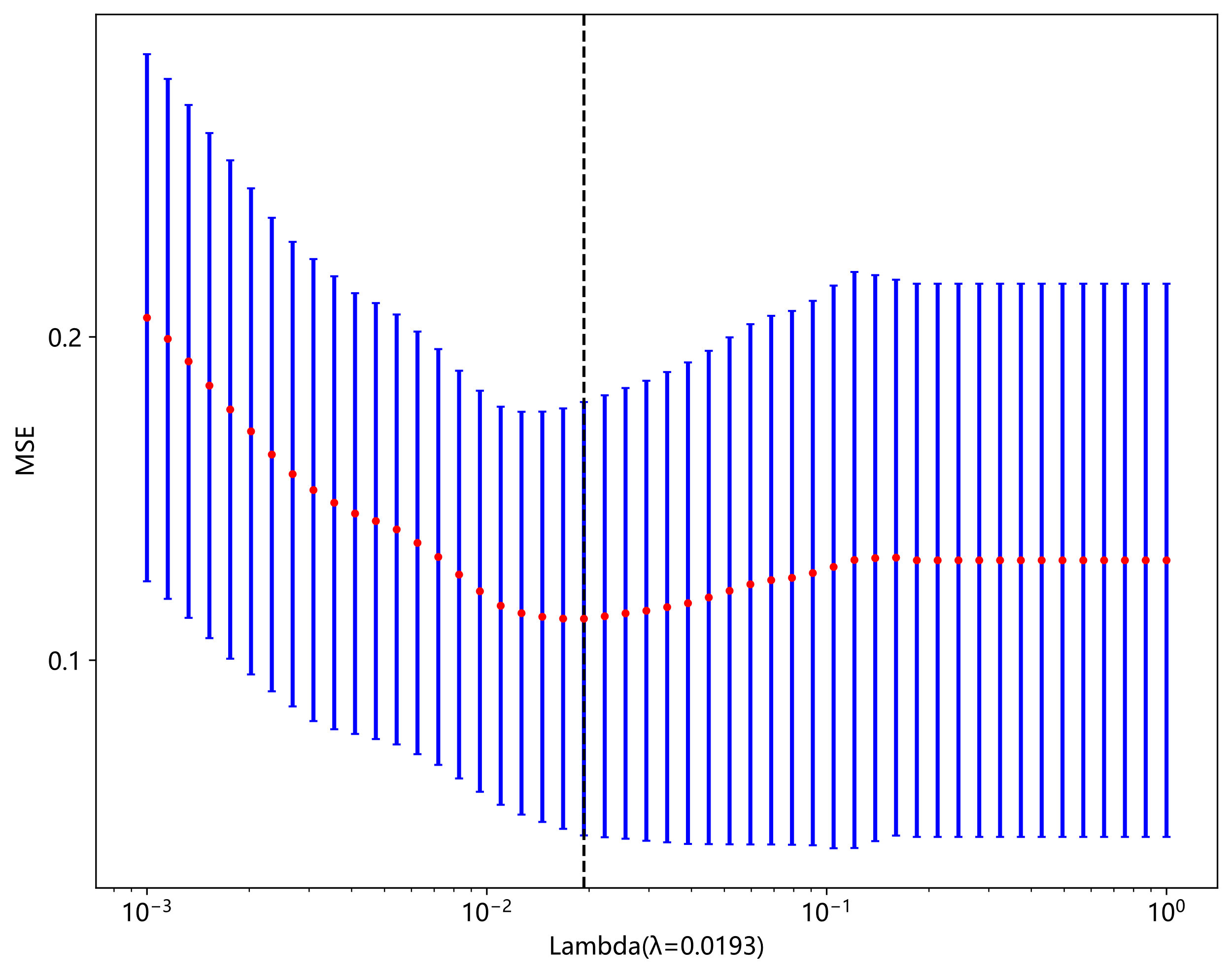


**B**


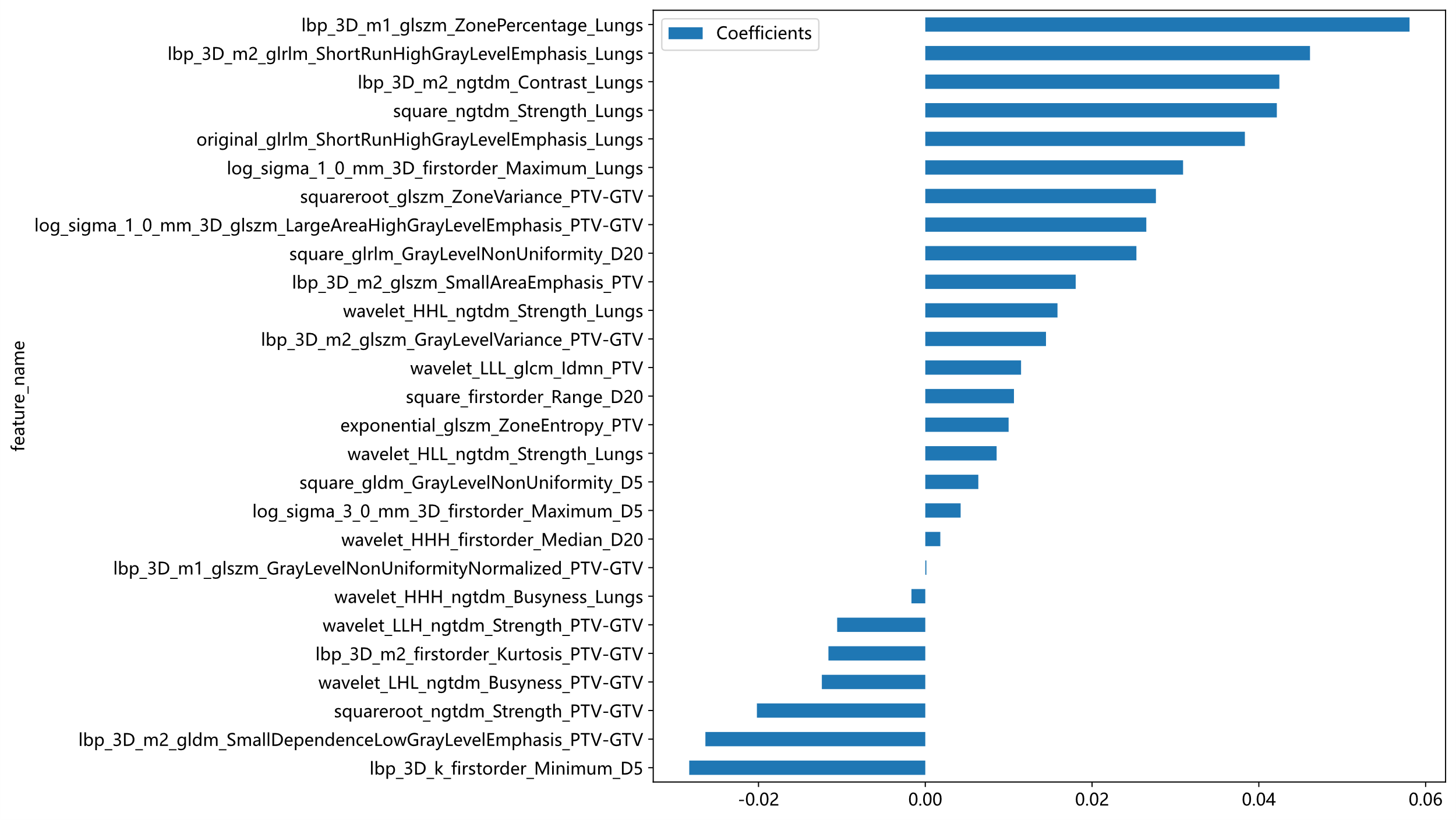


**C**


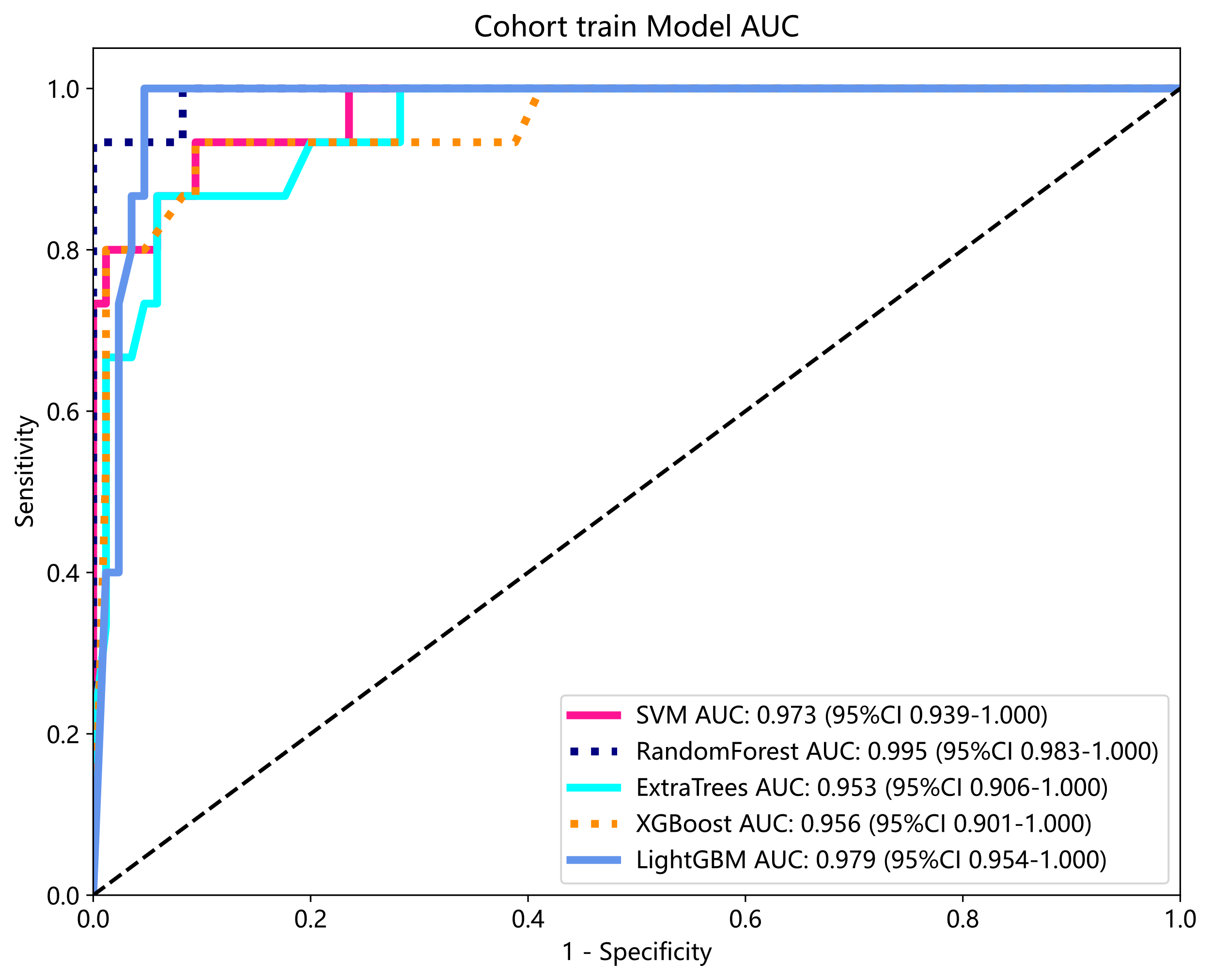


**D**


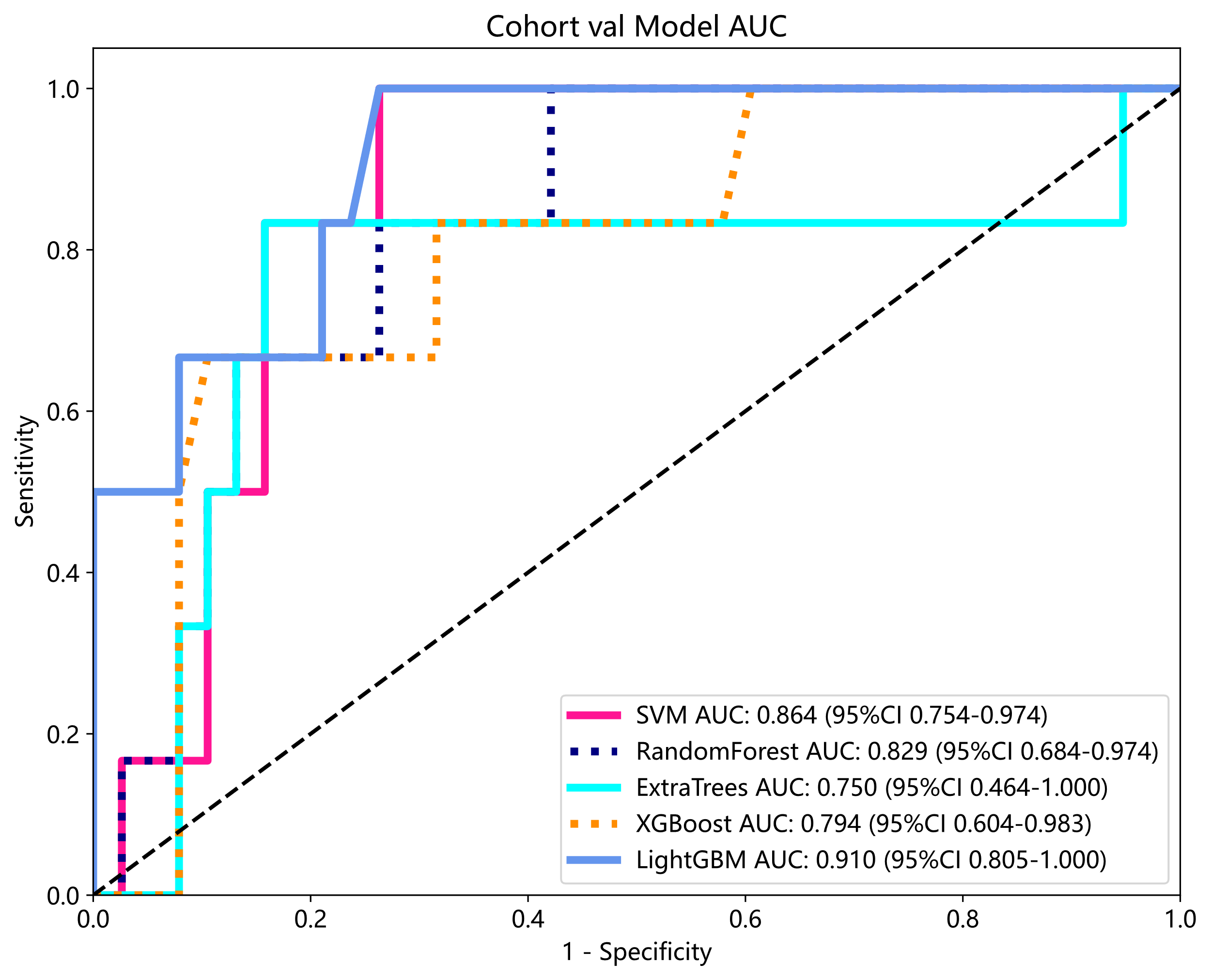


**E**


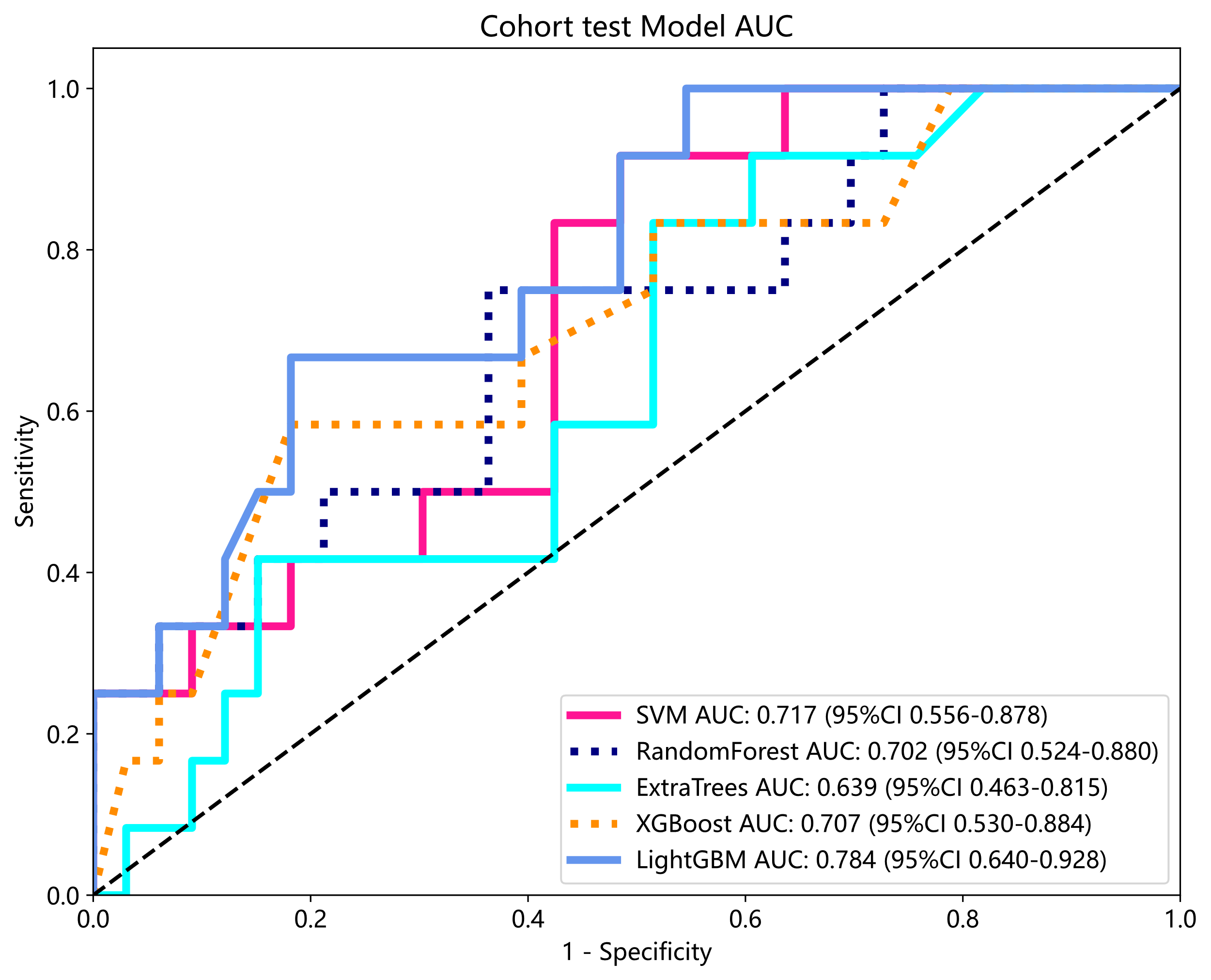


**F**

Figure S10. Coefficients(A), Mean standard error(B) and Weights(C) of 10 fold cross validation of RAP signatures, and ROC curves of machine learning models for RAP in train (D),validation(E), and testing cohort(F)

*Abbreviation: RAP:regions of anatomical dimension combined with physical dosimetric dimension*

Table S10. Metrics of RAP in three different tasks

| Model_name | Accuracy | AUC | 95% CI | Sensitivity | Specificity | PPV | NPV | Task |
| --- | --- | --- | --- | --- | --- | --- | --- | --- |
| SVM | 0.900 | 0.973 | 0.9388 - 1.0000 | 0.867 | 0.906 | 0.619 | 0.975 | Train |
| SVM | 0.750 | 0.864 | 0.7538 - 0.9743 | 0.833 | 0.737 | 0.333 | 0.966 | Val |
| SVM | 0.600 | 0.717 | 0.5565 - 0.8779 | 0.833 | 0.515 | 0.385 | 0.895 | Test |
| RandomForest | 0.980 | 0.995 | 0.9831 - 1.0000 | 0.867 | 1.000 | 1.000 | 0.977 | Train |
| RandomForest | 0.614 | 0.829 | 0.6841 - 0.9738 | 0.833 | 0.579 | 0.238 | 0.957 | Val |
| RandomForest | 0.644 | 0.702 | 0.5245 - 0.8795 | 0.667 | 0.636 | 0.400 | 0.840 | Test |
| ExtraTrees | 0.920 | 0.953 | 0.9064 - 1.0000 | 0.800 | 0.941 | 0.706 | 0.964 | Train |
| ExtraTrees | 0.818 | 0.750 | 0.4645 - 1.0000 | 0.667 | 0.842 | 0.400 | 0.941 | Val |
| ExtraTrees | 0.556 | 0.639 | 0.4627 - 0.8151 | 0.750 | 0.485 | 0.346 | 0.842 | Test |
| XGBoost | 0.900 | 0.956 | 0.9013 - 1.0000 | 0.867 | 0.906 | 0.619 | 0.975 | Train |
| XGBoost | 0.864 | 0.794 | 0.6042 - 0.9835 | 0.500 | 0.921 | 0.500 | 0.921 | Val |
| XGBoost | 0.733 | 0.707 | 0.5297 - 0.8844 | 0.250 | 0.909 | 0.500 | 0.769 | Test |
| LightGBM | 0.940 | 0.979 | 0.9545 - 1.0000 | 0.867 | 0.953 | 0.765 | 0.976 | Train |
| LightGBM | 0.773 | 0.910 | 0.8048 - 1.0000 | 0.833 | 0.763 | 0.357 | 0.967 | Val |
| LightGBM | 0.756 | 0.784 | 0.6397 - 0.9285 | 0.583 | 0.818 | 0.538 | 0.844 | Test |

*Abbreviation: RAP: regions of anatomical dimension combined with physical dosimetric dimension; AUC:areas under the curve;CI:confidence interval;PPV:positive predictive value; NPV:negative predictive value;SVM:support vector machine.*


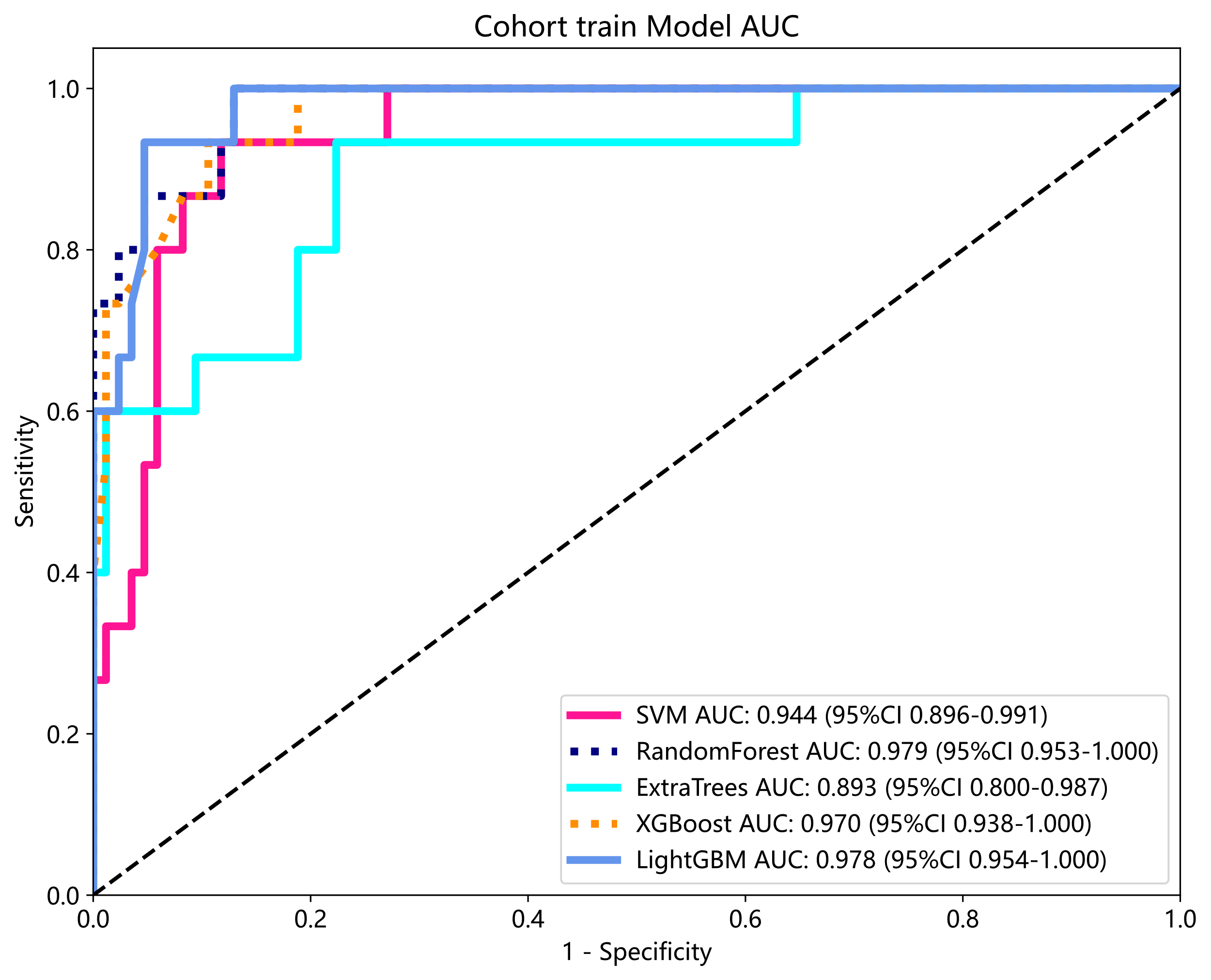


**A**


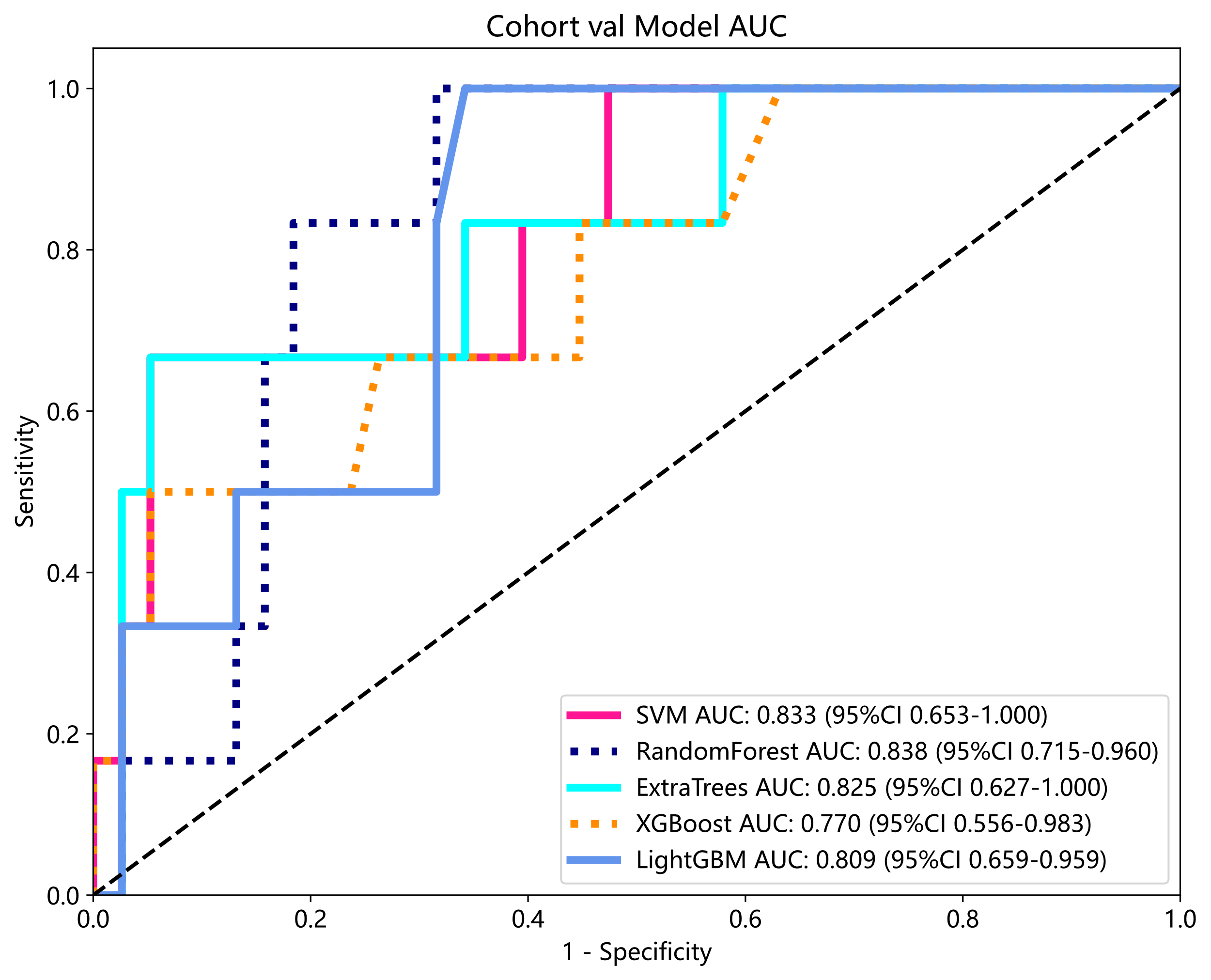


**B**


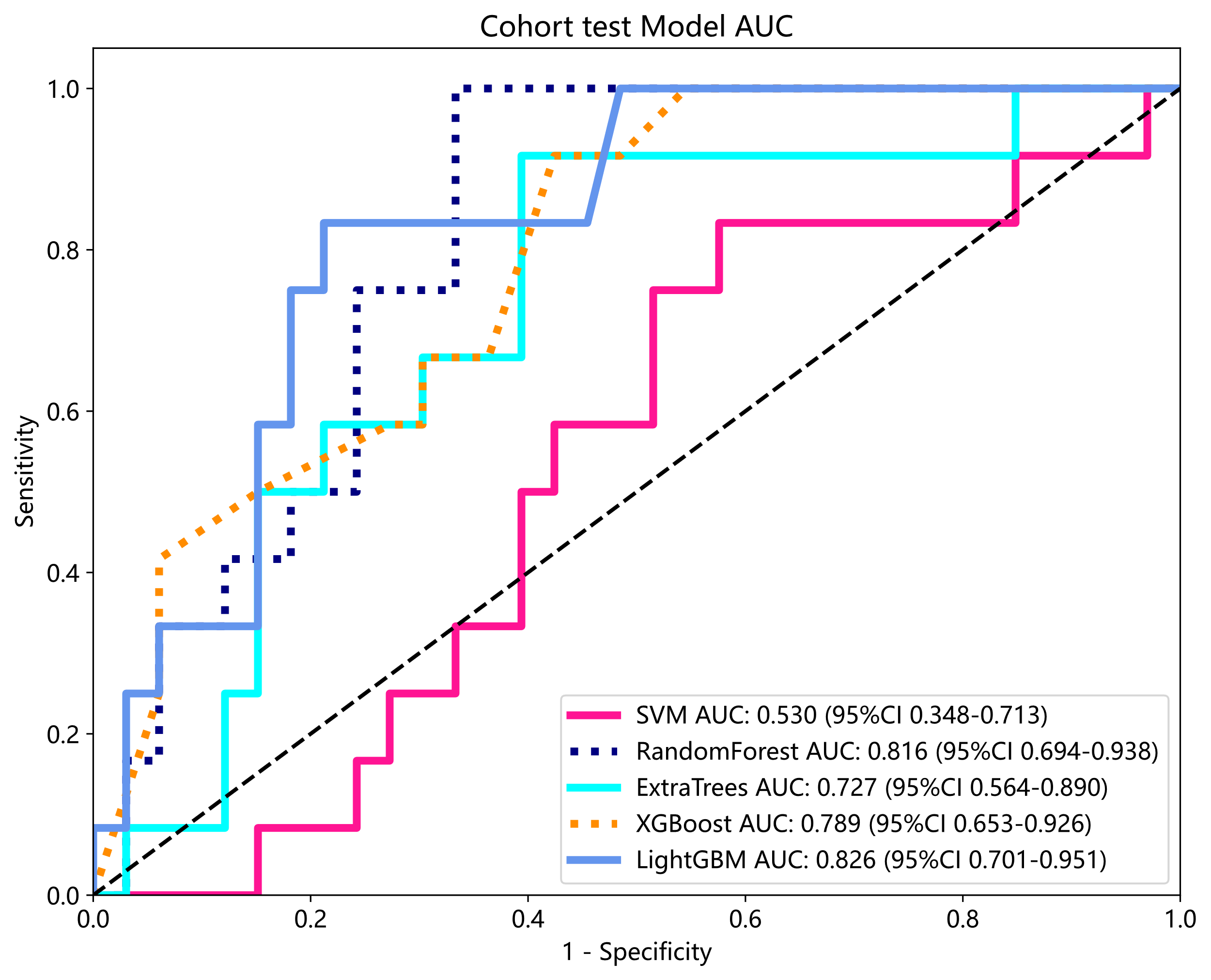


**C**

Figure S11. ROC curves of machine learning models for RAPB in train (A),validation(B), and testing cohort(C)

*Abbreviation:RAPB: regions of anatomical dimension combined with physical dosimetric and BED dimension.*

Table S11. Comparative performance metrics of different machine learning models for

clinical tasks in the RAPB model.

| Model_name | Accuracy | AUC | 95% CI | Sensitivity | Specificity | PPV | NPV | Threshold | Task |
| --- | --- | --- | --- | --- | --- | --- | --- | --- | --- |
| SVM | 0.880 | 0.944 | 0.8962 - 0.9908 | 0.867 | 0.882 | 0.565 | 0.974 | 0.500 | Train |
| SVM | 0.886 | 0.833 | 0.6529 - 1.0000 | 0.500 | 0.947 | 0.600 | 0.923 | 0.735 | Val |
| SVM | 0.511 | 0.530 | 0.3475 - 0.7131 | 0.750 | 0.424 | 0.321 | 0.824 | 0.056 | Test |
| RandomForest | 0.880 | 0.979 | 0.9534 - 1.0000 | 0.933 | 0.871 | 0.560 | 0.987 | 0.393 | Train |
| RandomForest | 0.705 | 0.838 | 0.7150 - 0.9604 | 0.833 | 0.684 | 0.294 | 0.963 | 0.296 | Val |
| RandomForest | 0.733 | 0.816 | 0.6935 - 0.9378 | 0.917 | 0.667 | 0.500 | 0.957 | 0.268 | Test |
| ExtraTrees | 0.790 | 0.893 | 0.7999 - 0.9868 | 0.867 | 0.776 | 0.406 | 0.971 | 0.443 | Train |
| ExtraTrees | 0.886 | 0.825 | 0.6268 - 1.0000 | 0.500 | 0.947 | 0.600 | 0.923 | 0.528 | Val |
| ExtraTrees | 0.667 | 0.727 | 0.5641 - 0.8904 | 0.833 | 0.606 | 0.435 | 0.909 | 0.368 | Test |
| XGBoost | 0.890 | 0.970 | 0.9376 - 1.0000 | 0.867 | 0.894 | 0.591 | 0.974 | 0.453 | Train |
| XGBoost | 0.864 | 0.770 | 0.5561 - 0.9834 | 0.333 | 0.947 | 0.500 | 0.900 | 0.603 | Val |
| XGBoost | 0.644 | 0.789 | 0.6525 - 0.9257 | 0.667 | 0.636 | 0.400 | 0.840 | 0.406 | Test |
| LightGBM | 0.940 | 0.978 | 0.9543 - 1.0000 | 0.867 | 0.953 | 0.765 | 0.976 | 0.556 | Train |
| LightGBM | 0.705 | 0.809 | 0.6594 - 0.9590 | 0.833 | 0.684 | 0.294 | 0.963 | 0.396 | Val |
| LightGBM | 0.778 | 0.826 | 0.7007 - 0.9508 | 0.750 | 0.788 | 0.562 | 0.897 | 0.458 | Test |

*Abbreviation: RAPB: regions of anatomical dimension combined with physical dosimetric and biologically effective dose; AUC:areas under the curve;CI:confidence interval;PPV:positive predictive value; NPV:negative predictive value;SVM:support vector machine.*


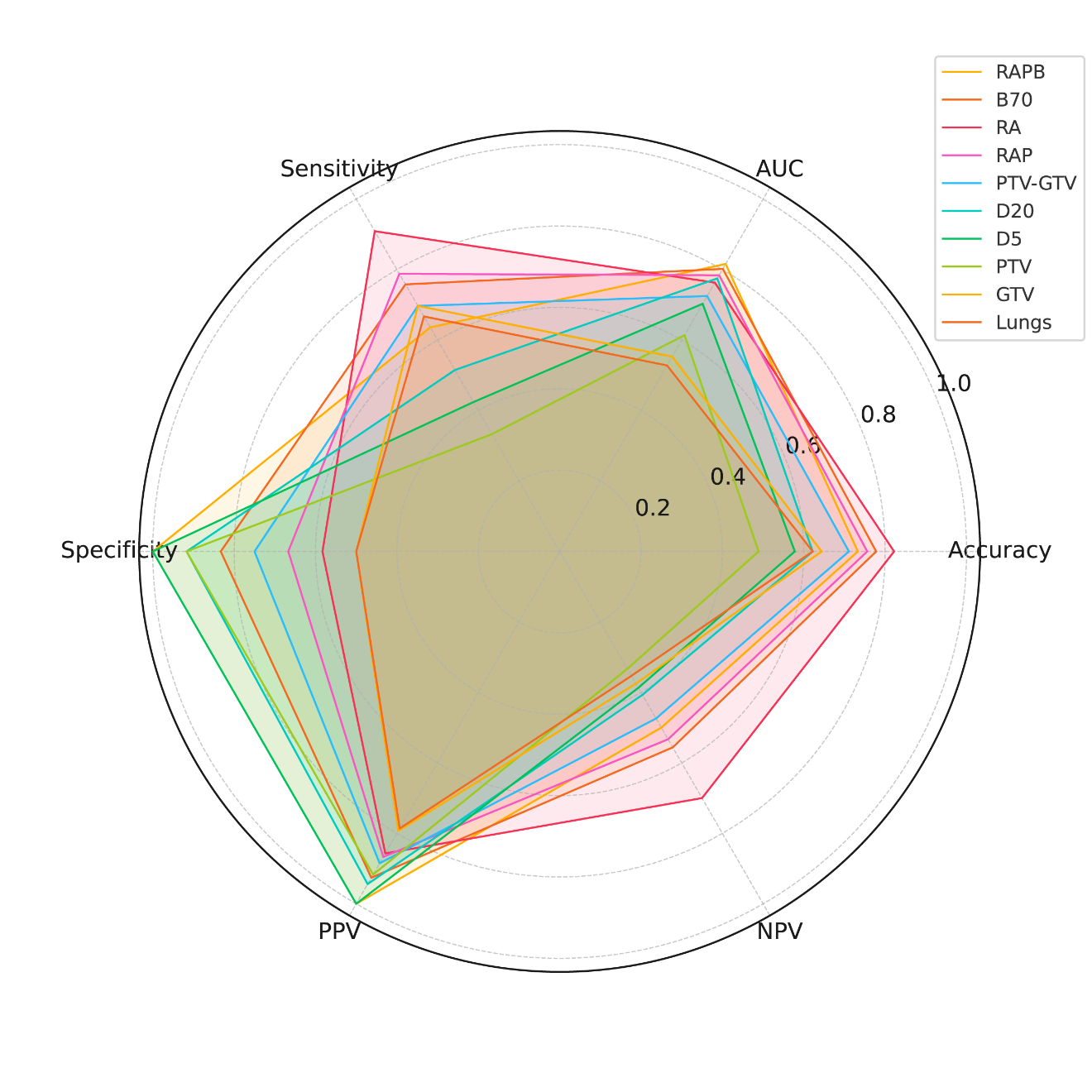
 Figure S12. Prediction performance of different radiomics models for predicting SRP in the testing cohort.

*Abbreviation: AUC: areas under the curve; PPV:positive predictive value; NPV: negative predictive value; GTV: gross tumor volume; PTV: planning target volume; RA:regions of anatomical demension; RAP: regions of anatomical dimension combined with physical dosimetric dimension; RAPB: regions of anatomical dimension combined with physical dosimetric and biologically effective dose.*


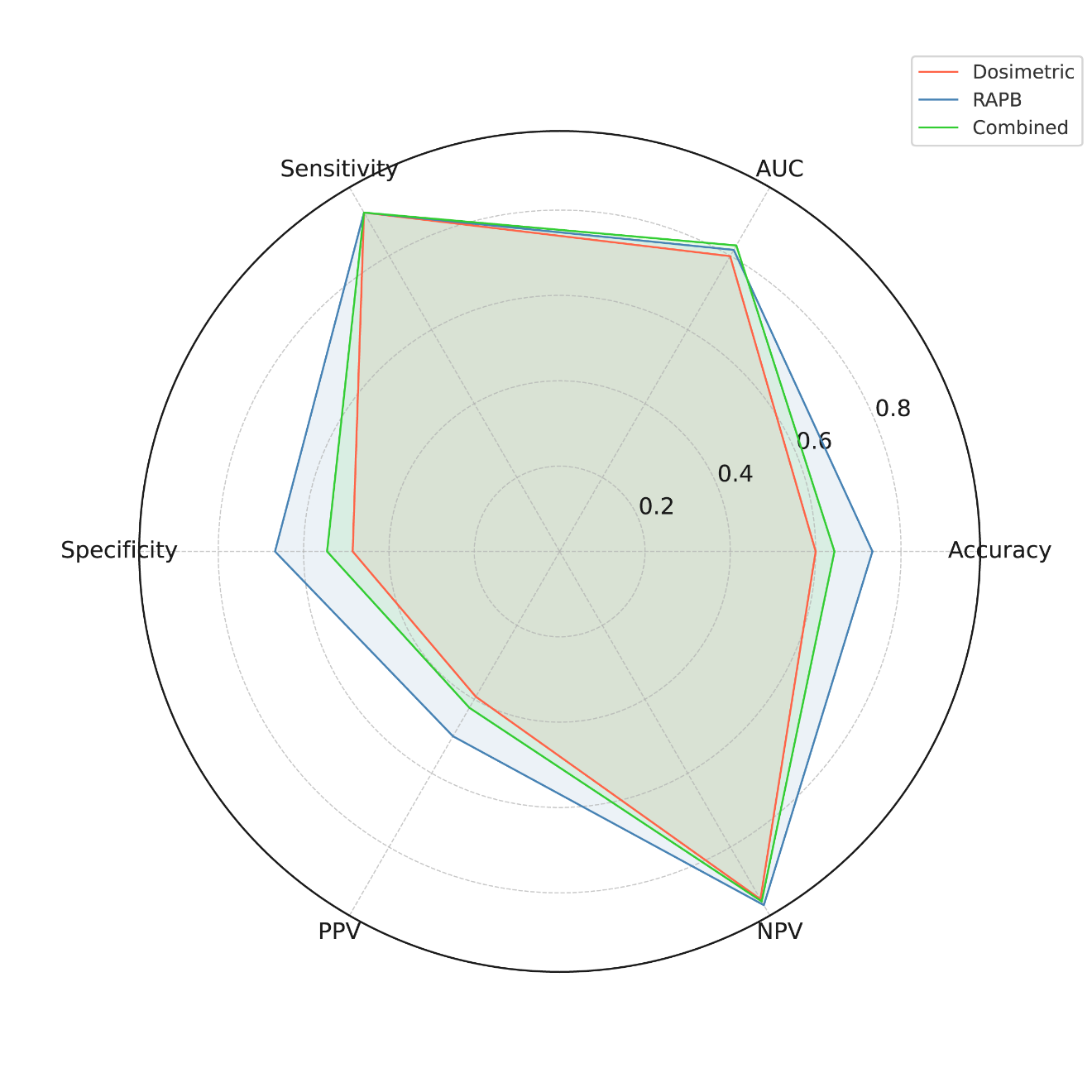
 Figure S13. Prediction performance of Dosimetric model, RAPB model, and Combined model for predicting SRP in the testing cohort.

*Abbreviation: AUC: areas under the curve; PPV:positive predictive value; NPV: negative predictive value; RAPB: regions of anatomical dimension combined with physical dosimetric and biologically effective dose.*
